# Loss of cellular RNA homeostasis contributes to MDA5 activation during virus infection

**DOI:** 10.1038/s41590-026-02614-3

**Published:** 2026-08-11

**Authors:** Natália G. Sampaio, Tanja Davis, Linden J. Gearing, Antonio G. Dias Junior, Lise Chauveau, Georgie Wray-McCann, Valerie Odon, Vladyslava Liudkovska, Alexandra L. McAllan, Chiara Cursi, Alice Mayer, Madara Ratnadiwakara, Minna-Liisa Änkö, Maciej Cieśla, Paul J. Hertzog, Jan Rehwinkel

**Affiliations:** 1 Medical Research Council Translational Immune Discovery Unit, Medical Research Council Weatherall Institute of Molecular Medicine, Radcliffe Department of Medicine, University of Oxford, Oxford, UK; 2 Centre for Innate Immunity and Infectious Diseases, Hudson Institute of Medical Research, Clayton, Victoria Australia; 3 Department of Molecular and Translational Sciences, School of Clinical Sciences, Monash University, Clayton, Victoria Australia; 4 RNA viruses and metabolism team, Institut de Recherche en Infectiologie de Montpellier (IRIM), CNRS, University of Montpellier, Montpellier, France; 5 IMol Polish Academy of Sciences, Warsaw, Poland; 6 Centre for Cancer Research, Hudson Institute of Medical Research, Clayton, Victoria Australia; 7 Faculty of Science and Engineering, Åbo Akademi University, Turku, Finland

**Keywords:** RIG-I-like receptors, Viral infection

## Abstract

MDA5 is an innate immune RNA sensor that senses infection with a range of viruses and other pathogens. MDA5’s RNA agonists are not well defined. Here we used single-nucleotide resolution crosslinking and immunoprecipitation (iCLIP) to study its ligands. Of note, upon infection with SARS-CoV-2 or encephalomyocarditis virus, MDA5 bound overwhelmingly to cellular RNAs. Many binding sites were intronic and proximal to *Alu* elements and to potentially base-paired structures. Concomitantly, cytoplasmic levels of aberrant transcripts and intron-containing unspliced transcripts increased in infected cells and displayed enrichment of MDA5 iCLIP peaks. Moreover, overexpression of the splicing factor SRSF3 reduced aberrant transcription and abrogated MDA5 activation. Taken together, we propose that MDA5 surveys RNA processing fidelity and can detect infections by sensing perturbations of post-transcriptional events such as splicing.

## Abstract (web summary)

MDA5 detects double-stranded RNA during viral infections. This work shows that MDA5 binds aberrant host RNA induced during viral infection. Rescue of aberrant cytoplasmic RNA during infection abrogated MDA5 activation.

## Main

Pathogens are first detected by pattern recognition receptors (PRRs) that activate immune signal transduction pathways and thereby initiate the host response to infection. Canonically, pathogen-derived molecules known as pathogen-associated molecular patterns (PAMPs) activate PRRs^1^. Viral infections are recognized by a class of PRRs that detect unusual nucleic acids^2^. A crucial response activated by nucleic acid sensors is the production of type I interferons (T1-IFNs)^3^. These cytokines are essential for protection against all viruses. This system is fine-tuned to differentiate infection from the homeostatic state, to elicit protective immunity followed by return to homeostasis. Excessive or uncontrolled T1-IFN responses fail to protect against infection and lead to long-term damage and disease^4,5^. Characterizing the initiation of T1-IFN responses is therefore fundamental to our ability to understand infectious and other diseases, and to design antiviral therapies.

Among the PRRs that detect viral infection are the retinoic acid-inducible gene I (RIG-I)-like receptors (RLRs)^6^. This family of RNA sensors includes RIG-I, melanoma differentiation-associated protein 5 (MDA5) and laboratory of genetics and physiology 2 (LGP2). RLRs are primarily located in the cytoplasm. All RLRs have a central helicase domain and a carboxy-terminal domain, which together detect unusual, immunostimulatory RNA molecules. MDA5 and RIG-I also contain two tandem amino-terminal caspase activation and recruitment domains (CARDs), which mediate downstream signaling through interaction with the adaptor mitochondrial antiviral-signaling protein (MAVS). Activated MAVS oligomerizes and recruits other factors including the kinase TBK1, that in turn activate IFN regulatory factors (IRFs) and the NF-κB pathway. Ultimately, expression of T1-IFNs and other immune response genes is induced. LGP2 lacks CARDs and modulates MDA5 and RIG-I signaling.

RIG-I and MDA5 are activated by different viral infections^6^. For example, influenza A virus (IAV) infection is sensed by RIG-I; conversely, picornaviruses such as encephalomyocarditis virus (EMCV) or rhinovirus are detected by MDA5^7^. Viruses from other families are recognized by both RIG-I and MDA5, including important human pathogens such as members of the *Flaviviridae* (for example Zika virus, hepatitis C virus), *Paramyxoviridae* (for example measles virus) and *Coronaviridae* (for example SARS-CoV)^8^. SARS-CoV-2 infection has been reported to be partially or exclusively sensed by MDA5 (refs. ^9–12^). Additionally, MDA5 can be activated by infection with non-RNA viruses^13–15^ and other pathogens such as *Plasmodium* sp.^16,17^, *Mycobacterium* *tuberculosis*^18^ or *Aspergillus* *fumigatus*^19^. The noteworthy role of MDA5 in the human immune response is highlighted by cases of inherited MDA5 deficiency leading to increased and/or life-threatening susceptibility to viral infections, for example with Rhinovirus^20–23^.

An important aspect of RLR signaling is the ability of these receptors to distinguish immunostimulatory RNAs accumulating in infected cells from the RNA content of cells during homeostasis. RIG-I is activated by RNAs with triphosphate or diphosphate groups at the 5′ end^24–26^. Additional features of RIG-I-stimulatory RNAs include base-pairing and the lack of methylation marks^27,28^. Such RNA species do not occur abundantly in cells, as most cellular RNAs are processed at the 5′ end; for example, mRNAs are capped and methylated. In contrast, some viral RNAs such as the genomes of IAV have those features, allowing them to be recognized by RIG-I^29^. As such, RNA sensing by RIG-I can be conceptualized by the paradigm of PAMP detection by PRRs. It is noteworthy that some viruses, including DNA viruses and retroviruses that do not produce viral 5′-(P)PP-RNAs, activate RIG-I indirectly by causing the accumulation of unprocessed and/or mislocalized cellular noncoding RNAs with 5′-PPP moieties^30–33^.

The RNA species that activate MDA5 are less well defined^8^. Early work showed that MDA5 recognizes infection with picornaviruses and the synthetic double-stranded (ds) RNA mimic polyriboinosinic:polyribocytidylic acid (Poly(I:C))^7,34^. In particular, MDA5 is essential for innate immune sensing of long Poly(I:C)^35^. Moreover, purified MDA5 forms multimeric filaments on long strands of dsRNA^36^. RNA viruses with positive-sense genomes, including picornaviruses, replicate by producing a negative-sense copy of their genome that serves as a template for synthesis of progeny positive-sense genomes. Annealing of positive and negative-sense RNAs can form long dsRNA, known as replicative form dsRNA, which was reported to activate MDA5 (refs. ^37,38^). Together, these results indicate that, in virally infected cells, MDA5 is activated by long viral dsRNA.

However, other findings contradict this view, suggestive of a more complex mechanism. Indeed, we previously demonstrated that higher-order structured RNA produced during infection, which may contain single-stranded RNA and dsRNA, rather than merely dsRNA, activates MDA5 (ref. ^39^). Moreover, studies analyzing the RNAs bound by MDA5 or LGP2 during viral infections suggested that single-stranded sections of viral genomes, rather than dsRNA forms, are detected^40–42^; however, these studies are constrained by methodological factors, including nonstringent purification of MDA5–RNA complexes, lack of precision in determining MDA5 binding sites, and/or exogenous protein overexpression. Given that MDA5 can sense infection with many different virus families and other pathogens, understanding the nature of MDA5’s RNA agonists is crucial for our comprehension of innate immune responses.

## Results

### Dual IP of endogenous MDA5 isolates MDA5-bound RNA

**Extended Data Fig. 1:**
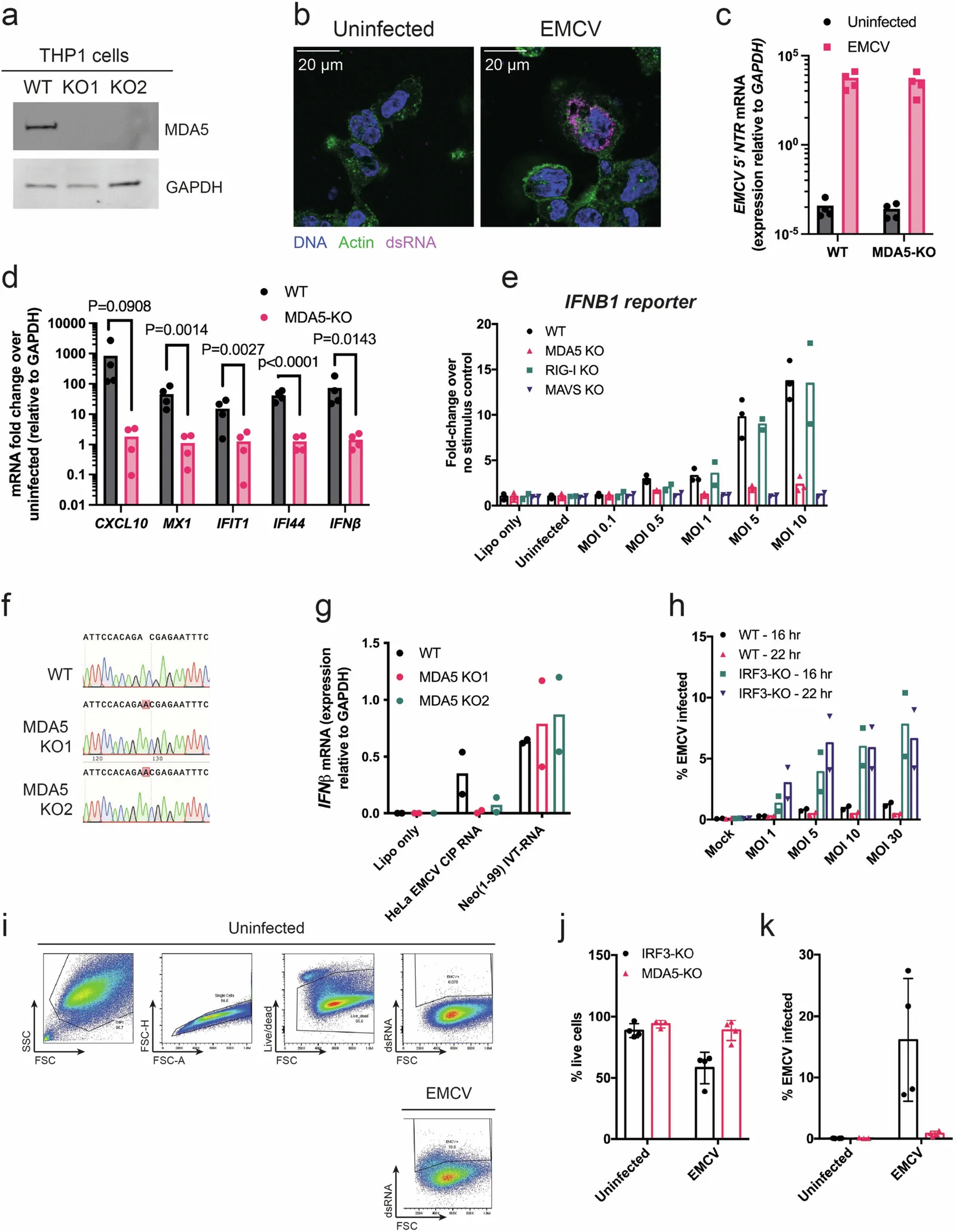
EMCV infects THP1 cells and induces an MDA5-dependent T1-IFN response. **a**, MDA5 protein levels in wild-type (WT) THP1 cells and in two independent clones of MDA5 knockout THP1 cells (KO1 and KO2) were analyzed by western blot. GAPDH served as a loading control. Representative of two independent experiments. **b**, THP1 cells were left uninfected or were infected with EMCV (MOI = 10) for 16 h, fixed and stained for dsRNA (J2 antibody; magenta), DNA (DAPI; blue) and actin (phalloidin; green). Images acquired by confocal microscopy are shown. **c-d**, WT and MDA5 knockout THP1 cells were infected with EMCV (MOI = 5) for 22 h. RNA was extracted and RT-qPCR performed for the EMCV 5’ non-translated region (NTR) (c) and the indicated mRNAs (d). Data are shown relative to GAPDH. **e**, RNA extracted from uninfected THP1 cells or from THP1 cells infected with EMCV for 24 h was transfected into HEK293 cells of the indicated genotypes stably expressing firefly luciferase under control of the IFNB promoter (p125HEK). As negative controls, p125HEK cells were treated with transfection reagent alone (Lipo only). After overnight incubation, luciferase activity in cell lysates was measured. Data from untreated cells were set to 1. **f**, Sanger sequencing of WT and MDA5-KO (KO1 and KO2) THP1 cells showed a 1 nucleotide insertion at position 23 in exon 1 of the IFIH1 gene. **g**, WT and MDA5 knockout THP1 cells were transfected with CIP-treated RNA from EMCV-infected HeLa cells (HeLa-EMCV CIP RNA) or with Neo1–99 IVT-RNA for 24 h. As a control, cells were also treated with Lipofectamine alone (Lipo only). IFNB1 mRNA levels were measured by RT-qPCR. Data are shown relative to GAPDH. **h**, WT and IRF3-KO THP1 cells were infected with EMCV at the indicated MOIs for 16 or 22 h. Cells were stained for dsRNA (J2) and analyzed by flow cytometry. **i**, Gating strategy for identification of EMCV-infected cells by flow cytometry using staining for dsRNA with J2 antibody. IRF3-KO THP1 cells uninfected or infected with EMCV (MOI = 5) for 22 hr are shown. FSC, forward scatter; SSC, side scatter. **j-k**, Proportion of live (**j**) and EMCV-infected (**k**) cells in samples used for iCLIP experiments (MOI = 5), measured by flow cytometry. Data in (**b**) are representative of three independent experiments. Data points in (**c**-**d**) represent four independent biological repeats, and bars represent mean. Data in (**e**) are technical replicates from one experiment, and bars represent the mean. Data points in (**g**-**h**) represent two independent biological repeats, and bars represent mean. Data in (**i**) are representative of four biological repeats. Data points in (**j**-**k**) represent four independent biological repeats, bars represent the mean, and error bars represent the standard error of the means from technical duplicates. Source data

Previous studies of RNAs binding to and/or activating MDA5 relied on recombinant or overexpressed protein^40,42,43^. To identify RNA ligands of endogenous MDA5, we screened cell lines for expression of MDA5 and found that monocytic THP1 cells expressed MDA5 protein at baseline (Extended Data Fig. 1a). EMCV specifically activates MDA5 (refs. ^7,44^) and infects THP1 cells, evident from staining with the J2 monoclonal antibody that detects dsRNA (Extended Data Fig. 1b), a signature of viral replication^39,45,46^, and from accumulation of viral RNA (Extended Data Fig. 1c). Infection induced *IFNB1* mRNA and IFN-stimulated genes (ISGs) (Extended Data Fig. 1d). Transfection of total cellular RNA extracted from EMCV-infected THP1 cells activated *IFNB1* promoter-driven luciferase expression in HEK293 reporter cells^47^ in an MDA5-dependent manner (Extended Data Fig. 1e). Together, these data show that MDA5-stimulatory RNA was present in EMCV-infected THP1 cells.

To obtain an in-depth view of RNAs binding MDA5 during viral infection, we employed iCLIP^48^. This technique allows deep sequencing of RNAs bound by a protein of interest, with resolution of the nucleotide site(s) where the protein binds, and has been successfully used for other dsRNA binding proteins including Dicer^49^ and DDX17 (ref. ^50^). As a control, MDA5 knockout (KO) THP1 cells were generated using CRISPR/Cas9 (Extended Data Fig. 1a,f). MDA5-KO THP1 did not induce *IFNB1* mRNA in response to transfection with total RNA extracted from EMCV-infected HeLa cells, but responded normally to RIG-I stimulation with in vitro transcribed RNA^29^ (Extended Data Fig. 1g). In MDA5-KO cells, T1-IFN responses to EMCV infection were undetectable (Extended Data Fig. 1d). Furthermore, we surmised that high infectivity levels would be required to detect RNA bound to MDA5; however, EMCV infectivity was low in wild-type (WT) THP1 cells (Extended Data Fig. 1h). IRF3 signals downstream of multiple nucleic acid sensors, including cGAS, which is required for baseline T1-IFN responses in THP1 cells^51^. Loss of IRF3 may therefore render cells more susceptible to EMCV without impairing MDA5–RNA interactions. Indeed, infectivity levels increased tenfold in IRF3-KO THP1 cells^47^ (Extended Data Fig. 1h–k). We therefore used IRF3-KO THP1 cells to investigate MDA5 ligands generated during EMCV infection.

**Fig. 1:**
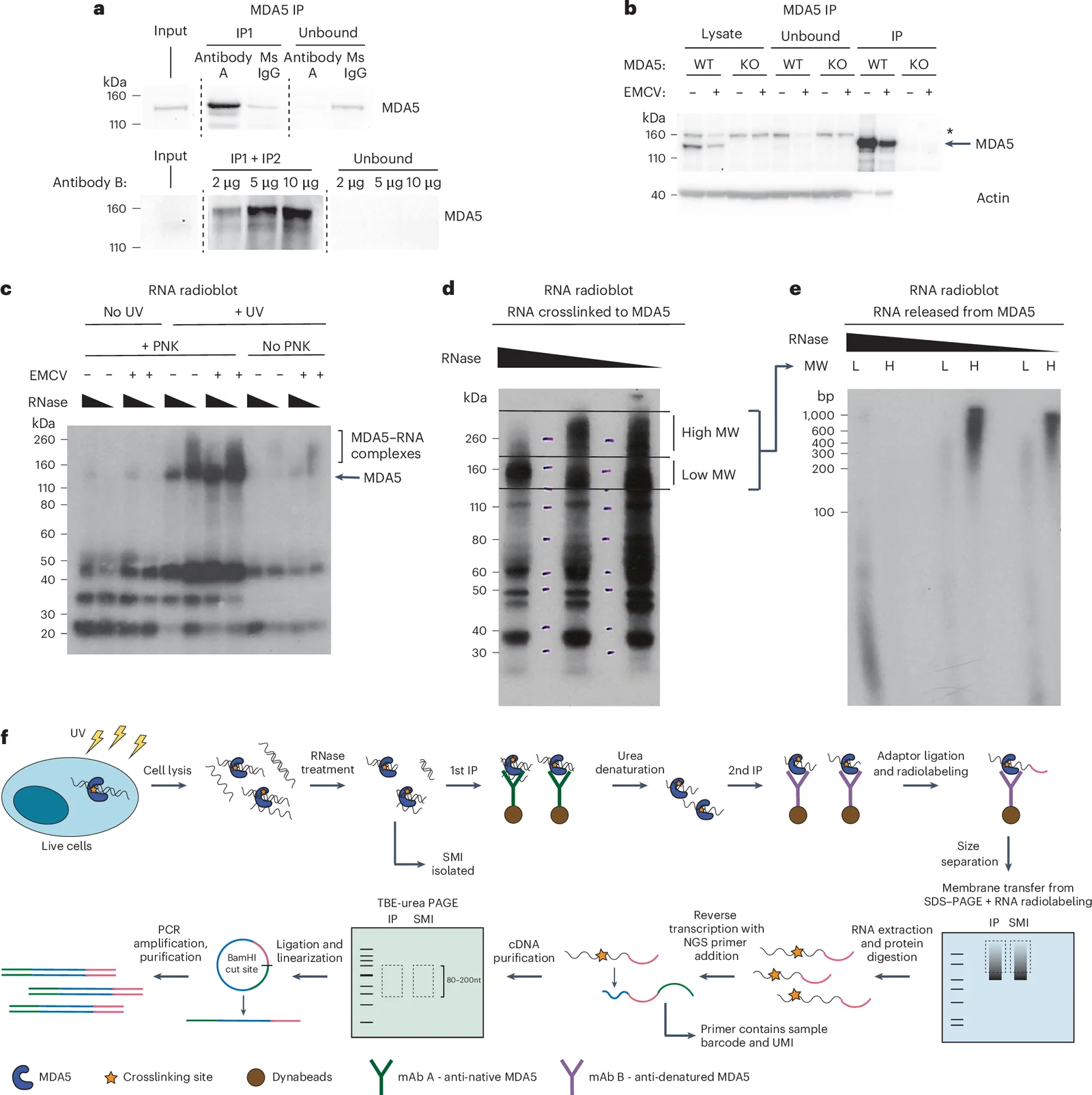
iCLIP isolates RNA bound by endogenous MDA5 in live cells. **a**, MDA5 IP was performed on WT THP1 cell lysate using antibody A or IgG control (IP1), or eluted under denaturing conditions and the eluted fraction was used for a second IP with antibody B using the indicated amounts of antibody (IP1 + IP2). Equivalent volumes of unbound fractions from the first and second IPs were collected. Cell lysate (input), IP and unbound fractions were analyzed by western blot for MDA5. **b**, MDA5-WT or MDA5-KO THP1 cells were infected with EMCV (MOI = 5) or left uninfected for 22 hr. Cells were lysed, and MDA5 dual IP with antibody A then B was performed. Cell lysates, unbound fractions and IP fractions were analyzed by western blot for MDA5. Actin was used as a loading control. The asterisk indicates a nonspecific band. **c**, Lysates from mock-infected or EMCV-infected (MOI = 10) WT THP1 cells, with or without UV crosslinking at 150 mJ cm^−2^, were treated with high or low concentrations of RNase A. MDA5 dual IP was performed (using Ab A and pooled ascites fluids from a selection of hybridoma clones including Ab B), followed by RNA radiolabeling using PNK enzyme, or no PNK as a negative control. Samples were resolved by SDS-PAGE, transferred to nitrocellulose membranes and exposed to radiofilm. **d**,**e**, Lysates from UV-crosslinked, EMCV-infected (MOI = 10) WT THP1 cells were treated with increasing concentrations of RNase A, followed by MDA5 dual IP (using Ab A and pooled ascites fluids from a selection of hybridoma clones including Ab B) and RNA radiolabeling. Samples were resolved by SDS–PAGE, transferred to nitrocellulose and exposed to radiofilm (**d**). High or low molecular weight (MW) sections of membrane from (**d**) were cut as indicated, and RNA extracted by proteinase K digestion, followed by TBE Urea gel electrophoresis and radioblot (**e**). **f**, Diagram of the MDA5 iCLIP method. SMI, size-matched input; input material run alongside IP sample and excised from membrane at same MW range. UMI, five bases added at random to allow removal of PCR duplicates during data processing. NGS, next-generation sequencing. All data are representative of at least two independent experiments. Source data

**Extended Data Fig. 2:**
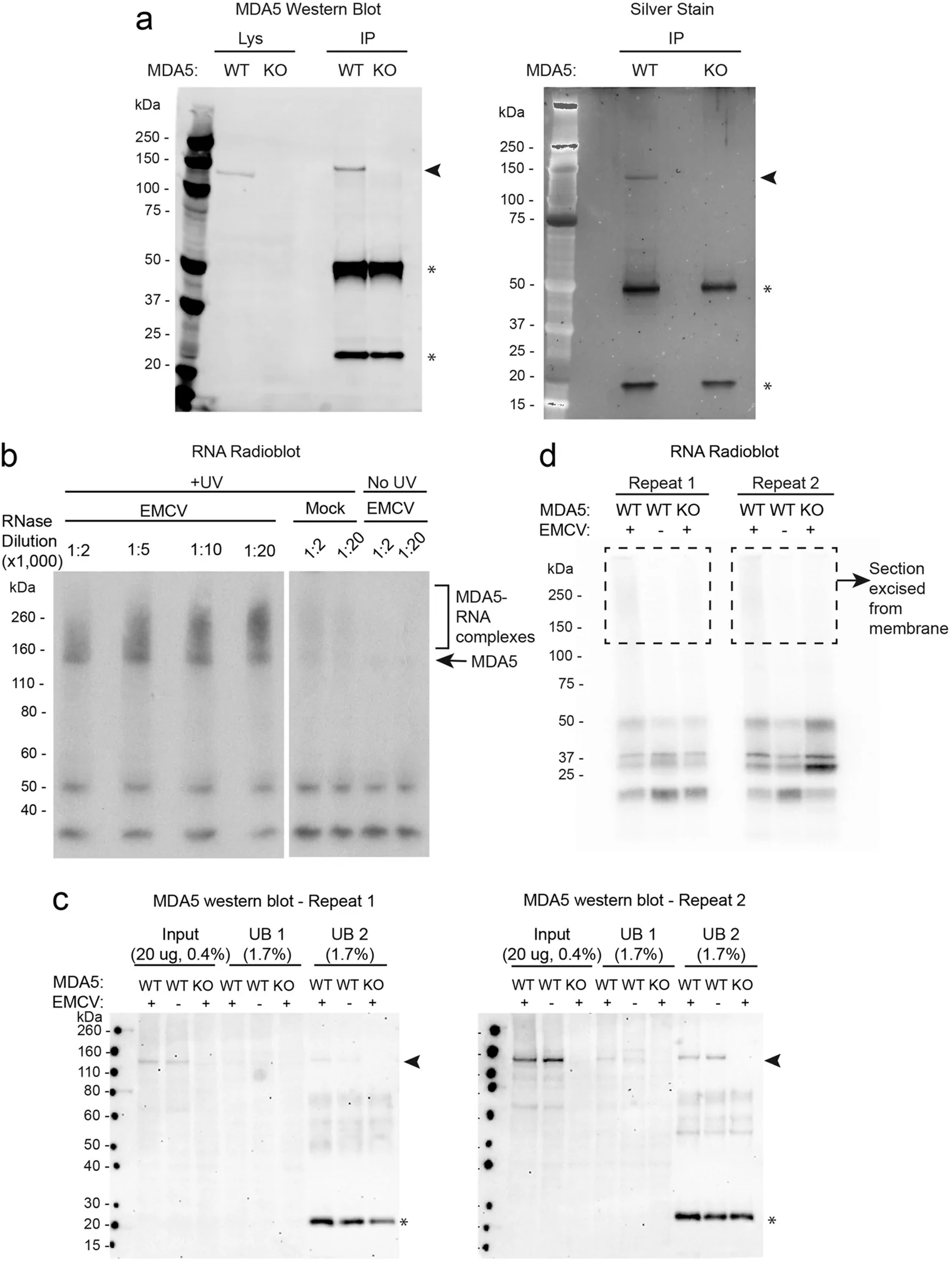
MDA5 immunoprecipitation and RNA radiolabeling. **a**, THP1 MDA5-WT or MDA5-KO cells were lysed and MDA5 IP was performed. Total cell lysate and IP were analyzed by SDS-PAGE followed by western blot against MDA5, or MDA5 IP was analyzed by SDS-PAGE and silver stain for total protein. MDA5 is indicated by black arrow. * = nonspecific IgG bands. Representative of two independent biological experiments. **b**, THP1 MDA5-WT cells were infected with EMCV (MOI = 10), or mock-infected, and UV-crosslinked ( + UV) or not crosslinked (No UV). Cell lysates were treated with RNase at indicated dilutions, prior to MDA5 IP (using Ab A and pooled ascites fluids from a selection of hybridoma clones including Ab B) and RNA radiolabeling. Samples were resolved by SDS-PAGE, transferred to nitrocellulose membrane and exposed to film. Data is from a single experiment. **c**, Example cell lysates and unbound fractions from MDA5 iCLIP IP from THP1 MDA5-WT or MDA5-KO cells, uninfected or infected with EMCV, resolved by SDS-PAGE followed by western blot against MDA5. MDA5 is indicated by black arrow. All bound material was used in (**d**). Data from two independent biological repeats are shown. **d**, Example RNA radioblot from radiolabelled MDA5 iCLIP samples as in (**c**), resolved by SDS-PAGE, transferred to nitrocellulose membrane and exposed to UV film. Sections excised from membrane and used for RNA extraction and iCLIP library generation are indicated. Data from two independent biological repeats are shown. Source data

To isolate endogenous MDA5, we raised a panel of monoclonal antibodies. We identified two antibodies that precipitated native and denatured MDA5, termed antibody A (clone 16) and antibody B (clone 22), respectively. To increase the stringency and specificity of MDA5 isolation, we used a dual immunoprecipitation (IP) method. Native MDA5 was first isolated using antibody A, and then eluted with a denaturing solution containing high urea and detergent concentrations. Next, the eluate containing denatured protein was used for a second IP with antibody B, which was specific for the denatured protein (Fig. 1a). This dual IP successfully pulled down MDA5 from THP1 cells (Fig. 1b). Silver staining confirmed the specificity of the MDA5 IP (Extended Data Fig. 2a)

Next, we performed MDA5 dual IP on lysates from UV-crosslinked cells, and radioactively labeled the bound RNA using polynucleotide kinase (PNK). Gel electrophoresis and radioblot analysis showed a high molecular weight (MW) smear migrating more slowly than the MW of MDA5 (135 kDa), which was dependent on both UV crosslinking and PNK treatment (Fig. 1c). The intensity of this smear was enhanced by virus infection, indicating increased RNA binding to MDA5 in infected cells (Fig. 1c and Extended Data Fig. 2b). RNase A treatment reduced the smear, presumably due to the bound RNA being partially degraded to smaller oligonucleotides protected by the protein (Fig. 1c,d). Extraction and proteinase K digestion of either high MW or low MW sections from the membrane produced RNA of varying lengths, with intermediate and low RNase A concentrations yielding RNA of more than 100 nt (Fig. 1e). We selected the low RNase A treatment condition to capture MDA5-bound RNA in the 100–500-nt-length range, which is suitable for iCLIP and sequencing^48^.

We then applied our dual IP approach to a modified iCLIP protocol^48^ (Fig. 1f). An adaptor for reverse transcription was added to RNA at the 3′ end, and the RNA was radiolabelled at the 5′ end, followed by isolation of RNA by gel electrophoresis and radioblot, where the total sample above the MW of MDA5 was isolated (Extended Data Fig. 2d). An aliquot of pre-IP input material was processed alongside the IP samples and served as a ‘size-matched input’ (SMI) control for comparison to IP samples^52^. Next, the RNA was reverse transcribed into cDNA using primers containing a sample-specific barcode for multiplexing and a unique molecular identifier (UMI) for identification of PCR duplicates. Of note, reverse transcription is halted at the RNA–protein crosslink site, which allows identification of nucleotide positions bound by MDA5. The cDNA was purified and size-selected by electrophoresis, circularized and cleaved to produce cDNA containing forward and reverse PCR sites for amplification and sequencing (Fig. 1f).

### MDA5 binds host-derived RNA during EMCV infection

**Extended Data Fig. 3:**
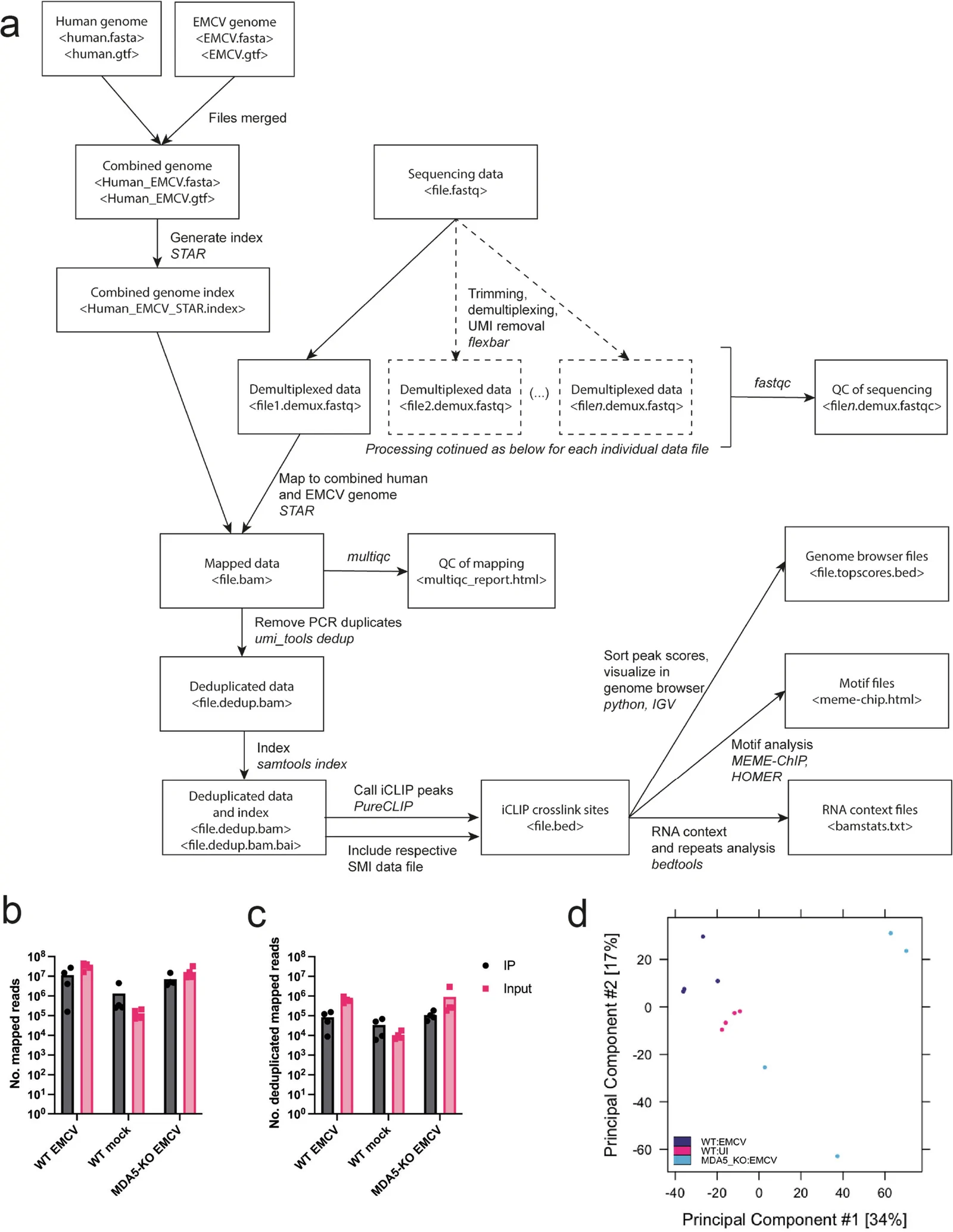
Workflow of iCLIP computational data analysis and mapping rates of MDA5 iCLIP sequencing reads. **a**, Input files are represented within boxes, with sample file names and extensions in angle brackets. Arrows indicate file transformations, and computational tools used are shown in italics. **b-c**, Number of iCLIP sequencing reads that mapped to the combined human and EMCV genome before (**b**) and after (**c**) removal of PCR duplicates. Data points in (**b**-**c**) are from four independent biological repeats, and bars represent the mean. **d**, PCA of MDA5 iCLIP binding sites from THP1 MDA5-WT and MDA5-KO cells, uninfected or infected with EMCV. Data points are from four independent biological repeats.

We employed our endogenous MDA5 iCLIP method to identify RNAs specifically bound by MDA5 during virus infection (Extended Data Fig. 2c,d). IRF3-KO THP1 cells were left uninfected or were infected with EMCV. As a negative control for IP specificity, MDA5-KO THP1 cells were used. EMCV infectivity was lower in MDA5-KO cells compared to IRF3-KO cells (henceforth referred to as MDA5-WT) (Extended Data Fig. 1i–k), likely due to baseline T1-IFN production via cGAS-IRF3 (ref. ^51^), priming cells against infection. Nevertheless, these cells lacked MDA5 and thus served as a control for MDA5 IP specificity. Sequencing reads were processed and mapped to combined human and virus genomes to simultaneously identify both viral and host sequences, followed by removal of PCR duplicates and extraction of crosslink sites (Extended Data Fig. 3a). We obtained 10^4^–10^6^ mapped, deduplicated reads per sample with four independent biological repeats (Extended Data Fig. 3b,c). Principal- component analysis (PCA) demonstrated that replicate samples separated according to treatment and genotype (Extended Data Fig. 3d).

**Fig. 2:**
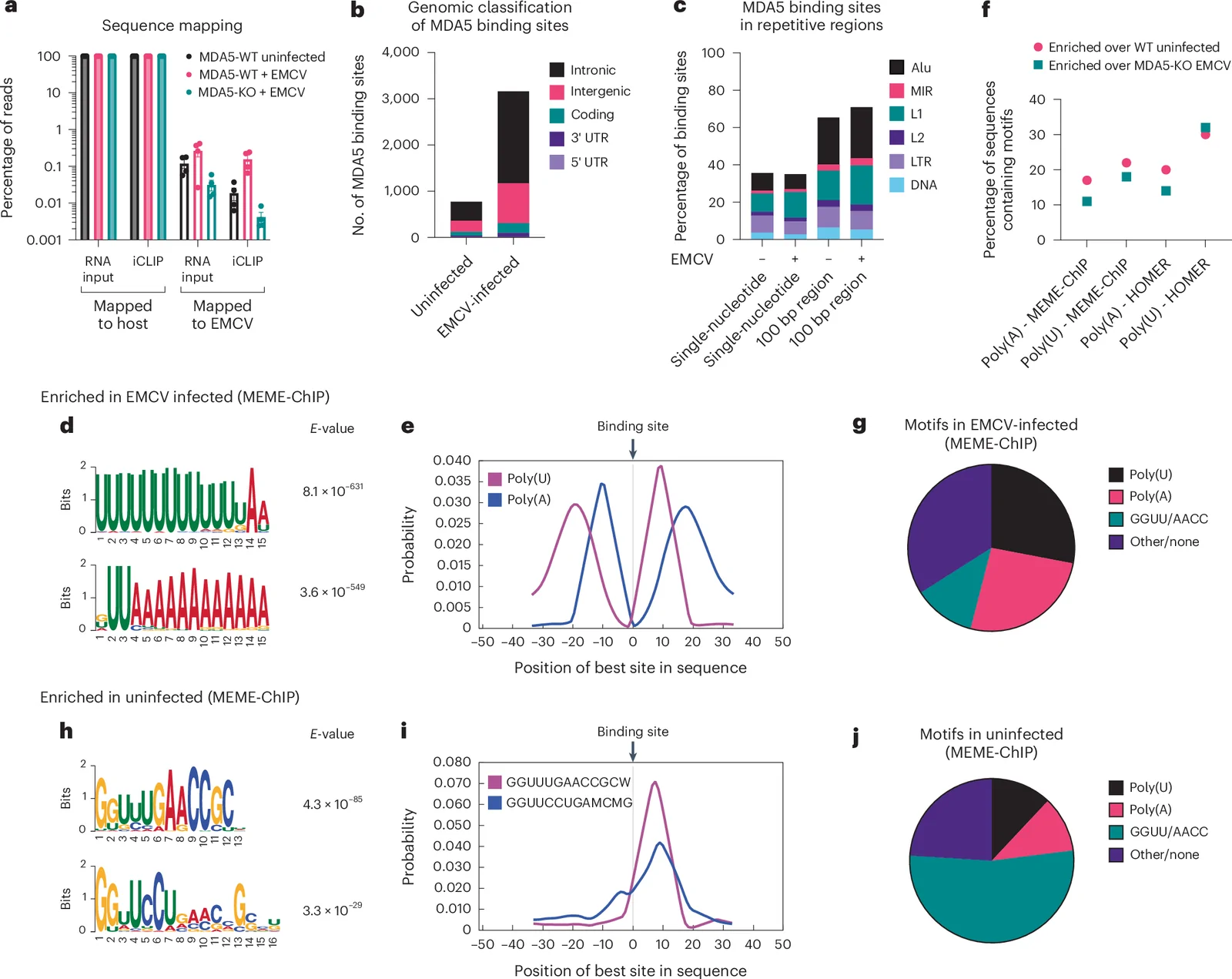
MDA5 binds cellular RNAs during EMCV infection. **a**, Percentage of RNA sequences in RNA input or MDA5 iCLIP samples mapping to the host or EMCV genome. **b**, Genomic classification of single-nucleotide MDA5 binding sites detected by iCLIP. **c**, Proportion of single-nucleotide MDA5 binding sites, or 101-nt regions (50 nt upstream and downstream of single-nucleotide binding site) that are located within repetitive regions of the human genome. **d–f**, Sequences consisting of 50 nt upstream and downstream of each MDA5 binding site (total length 101 nt) from MDA5-WT EMCV-infected samples were analyzed for de novo RNA motifs using MEME-ChIP. Specific motifs enriched in MDA5-WT-EMCV-infected samples compared to MDA5-WT uninfected samples, and associated *E*-values (adjusted *P* value multiplied by the number of motifs in the input file) are shown. Localization of the motifs discovered by MEME-ChIP relative to the crosslink site (position 0, black arrow) (**e**). The proportion of binding sites containing Poly(A) and Poly(U) motifs in MDA5-WT EMCV-infected samples when analyzed with either MEME-ChIP or HOMER algorithms as above (**f**). **g**, Proportion of motifs in MDA5 binding sites from EMCV-infected cells. **h–j**, Motifs enriched in MDA5-WT uninfected samples compared to the MDA5-KO-EMCV-infected negative control were analyzed as in **c–e**. M = A or C; W = A or U. Proportion of motifs in MDA5 binding sites from uninfected cells (**j**). Data points in **a** represent four independent biological repeats, and bars/error bars represent the mean and s.d. Analyses in **b–j** were conducted on combined data from four independent biological repeats. Source data

To our surprise, we found that the majority (>99%) of RNA sequences detected in iCLIP samples mapped to the human rather than the viral genome (Fig. 2a). There was no enrichment of EMCV RNA in the MDA5 IP compared to the RNA input control samples. We next identified MDA5-RNA crosslink sites, herein referred to as MDA5 binding sites, using PureCLIP^53^ that compares input and IP sequencing reads. MDA5 binding sites were only identified in host RNA, and not in viral RNA. The distribution of MDA5 binding sites between intergenic regions, introns, untranslated regions and coding sequences was similar between the samples, and more than half of all binding sites were in introns (Fig. 2b). Given that MDA5 has been previously suggested to bind repetitive RNA^43^, we determined the proportion of binding sites present in repetitive regions. Around 35% of binding sites were located within repetitive regions, which included SINEs (*Alu*, mammalian-wide interspersed repeat (MIR)), LINEs (L1 and L2) and other repeat categories (Fig. 2c). We then extended our analysis to 101-nt regions spanning binding sites (50 nt upstream and downstream of binding site) and found that ~65–71% of these regions overlapped with repetitive elements, indicating that a majority of RNAs bound by MDA5 contain repetitive elements.

**Extended Data Fig. 4:**
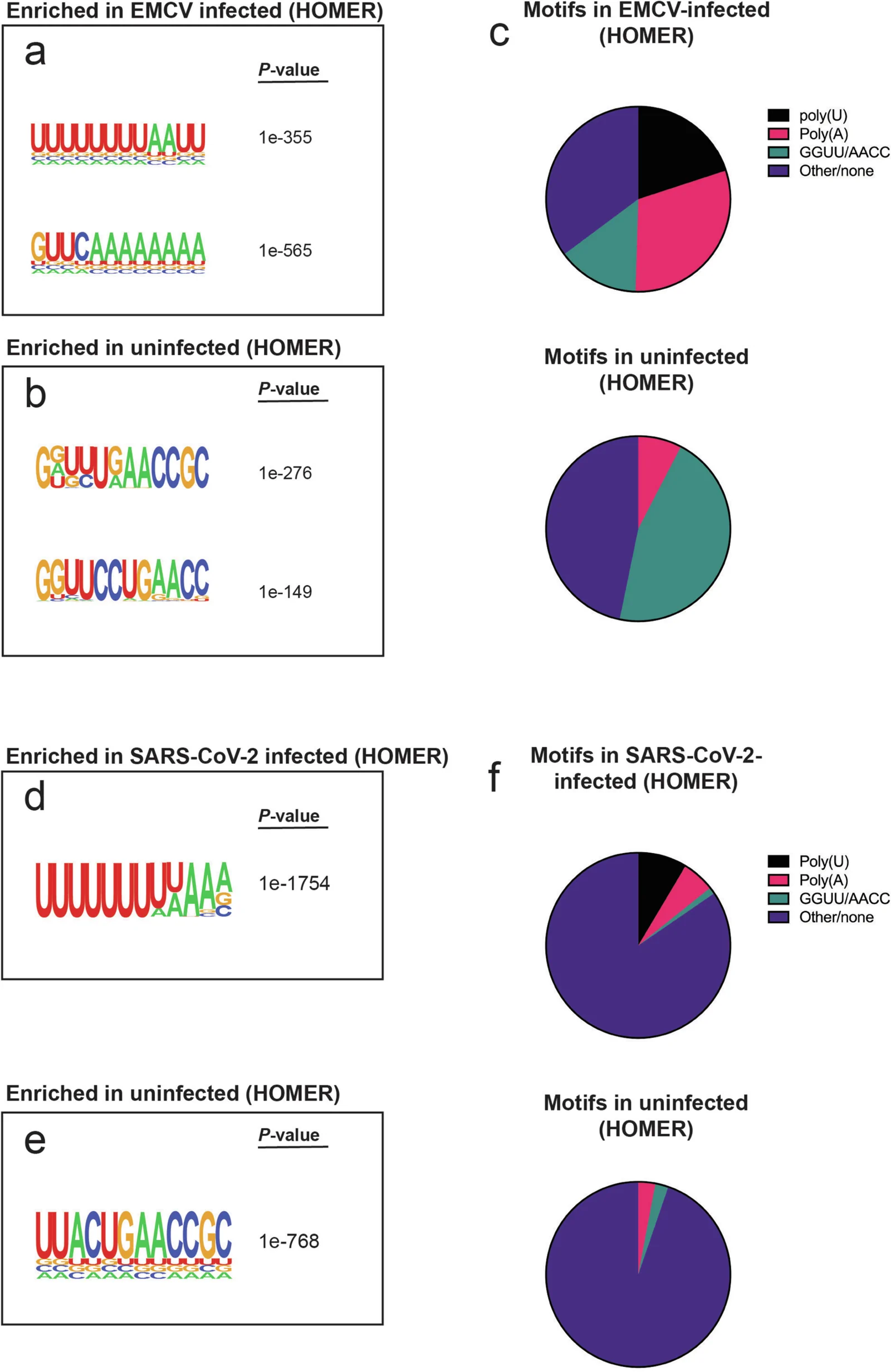
HOMER motif analysis of MDA5 binding sites from uninfected and virus-infected cells. 101 nt regions (50 nt upstream and downstream of single-nucleotide binding site) around MDA5 binding sites were analyzed for de novo RNA motifs using HOMER. **a-c**, Specific motifs enriched in EMCV-infected samples compared to uninfected samples (**a**), and in uninfected samples (**b**). Associated P-values are shown. Proportion of motifs in MDA5 binding sites from uninfected or EMCV-infected cells is shown in **c**. **d-f**, As above, but with SARS-CoV-2-infected cells. Source data

We then employed HOMER^54^ and MEME-ChIP^55^ to identify motifs enriched in MDA5-bound RNA. Based on MDA5’s footprint on dsRNA of 14–15 nt (ref. ^56^) and the notion of MDA5 filament formation^36^, we focused on the 101-nt-length regions containing MDA5 binding sites centrally. Both algorithms identified an enrichment of Poly(A) and Poly(U) sequences in MDA5 binding sequences in EMCV-infected cells (Fig. 2d and Extended Data Fig. 4a). These Poly(A)/(U) motifs were positioned near, but not directly overlapping, the MDA5 crosslinked nucleotide (Fig. 2e). Between 30% and 50% of all MDA5 binding sequences contained either a Poly(A) or a Poly(U) motif, depending on the control and motif algorithm applied (Fig. 2f,g). Of note, these motifs were enriched compared to uninfected, MDA5-sufficient cells and to infected, MDA5-deficient cells, indicative of specificity. Additionally, motifs that contained GGUU and AACC sequences were specifically enriched in MDA5 binding sites from uninfected MDA5-WT cells when compared to the infected MDA5-KO control (Fig. 2h,i and Extended Data Fig. 4b). These motifs were detected in 40–50% of binding sites in uninfected samples (Fig. 2j and Extended Data Fig. 4c). The GGUU/AACC motifs were also detected in MDA5 binding sites from EMCV-infected MDA5-WT samples (Fig. 2g and Extended Data Fig. 4c), but at a lower rate than uninfected MDA5-WT cells. Given that EMCV infection rate was ~10–25% (Extended Data Fig. 1k), infected samples contained both infected and uninfected cells. This suggests that the GGUU/AACC motifs may represent binding sites derived from uninfected cells within the infected cell population.

### Synthetic host RNAs encompassing MDA5 binding sites activate MDA5 in cellulo

**Fig. 3:**
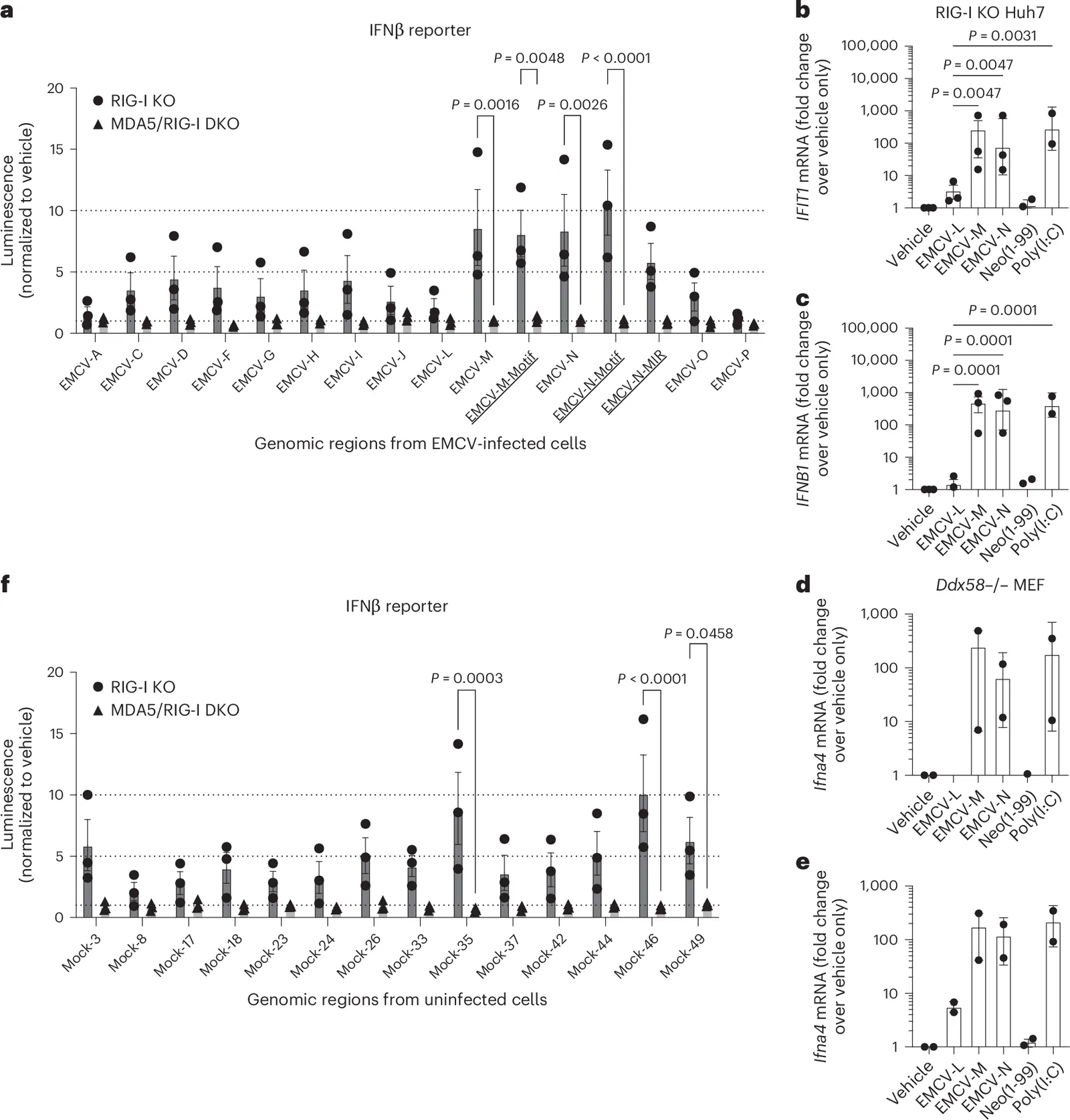
MDA5 is activated by host RNA during EMCV infection. **a**, IVT-RNAs from host genomic regions that were bound by MDA5 during EMCV infection were transfected into RIG-I-KO or RIG-I/MDA5-DKO p125HEK *IFNB1* reporter cells. As negative controls, cells were treated with transfection reagent alone (vehicle only). After overnight incubation, luciferase activity in cell lysates was measured. Data from vehicle only were set to 1. **b–e**, RIG-I-KO Huh7 cells (**b**,**c**) and *Ddx58*^*−/−*^ MEFs (**d**,**e**) were transfected with the indicated IVT-dsRNAs, Poly(I:C) or Neo^1–99^ IVT-RNA, or were treated with transfection reagent alone (vehicle only). After overnight incubation, RNA was extracted and RT–qPCR performed for *IFNB1* and *IFIT1* (Huh7), or *Ifna4* and *Ifi44* (MEFs). Data are shown relative to *GAPDH*. **f**, IVT-RNAs from host genomic regions that were bound by MDA5 in uninfected cells were transfected into RIG-I-KO or RIG-I/MDA5-DKO p125HEK *IFNB1* reporter cells as in **a**. Data points in (**a**–**c**,**f**) are from three independent experiments, data points in **d**,**e** and from two independent experiments. All data are presented as mean ± s.e.m. Statistical analysis for **a**,**f** was by two-way ANOVA with Šídák multiple comparisons test, and for **b**,**c**, was a one-way ANOVA with Holm–Šídák multiple comparisons test. Source data

**Extended Data Fig. 5:**
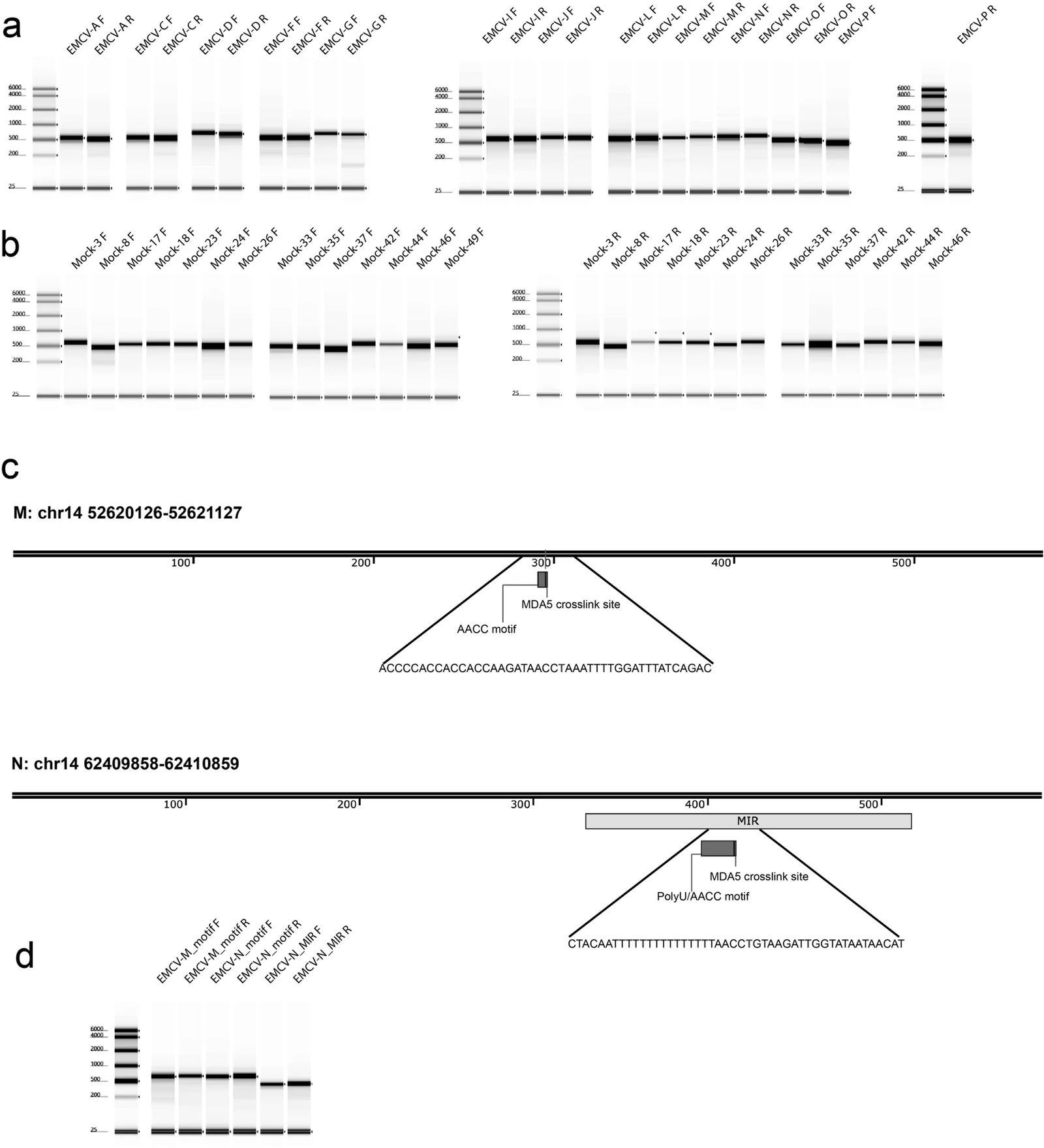
Preparation of IVT-RNAs from genomic sequences containing MDA5 iCLIP binding sites. **a**-**b**, Forward (F) and reverse (R) IVT-RNAs were generated from the selected regions from high-scoring MDA5 iCLIP crosslink sites from (**a**) EMCV-infected cells or (**b**) uninfected cells, and size and purity was assessed on Tapestation. **c**, IVT-RNAs from genomic regions ‘M’ and ‘N’ are depicted, with motifs or MIR regions outlined. The MDA5 crosslinked nucleotide is highlighted in dark gray. Either the motif only, or the MIR were removed from the sequence, and (**d**) forward (F) and reverse (R) IVT-RNAs were generated and assessed on Tapestation. Data is from one experiment. Source data

**Extended Data Fig. 6:**
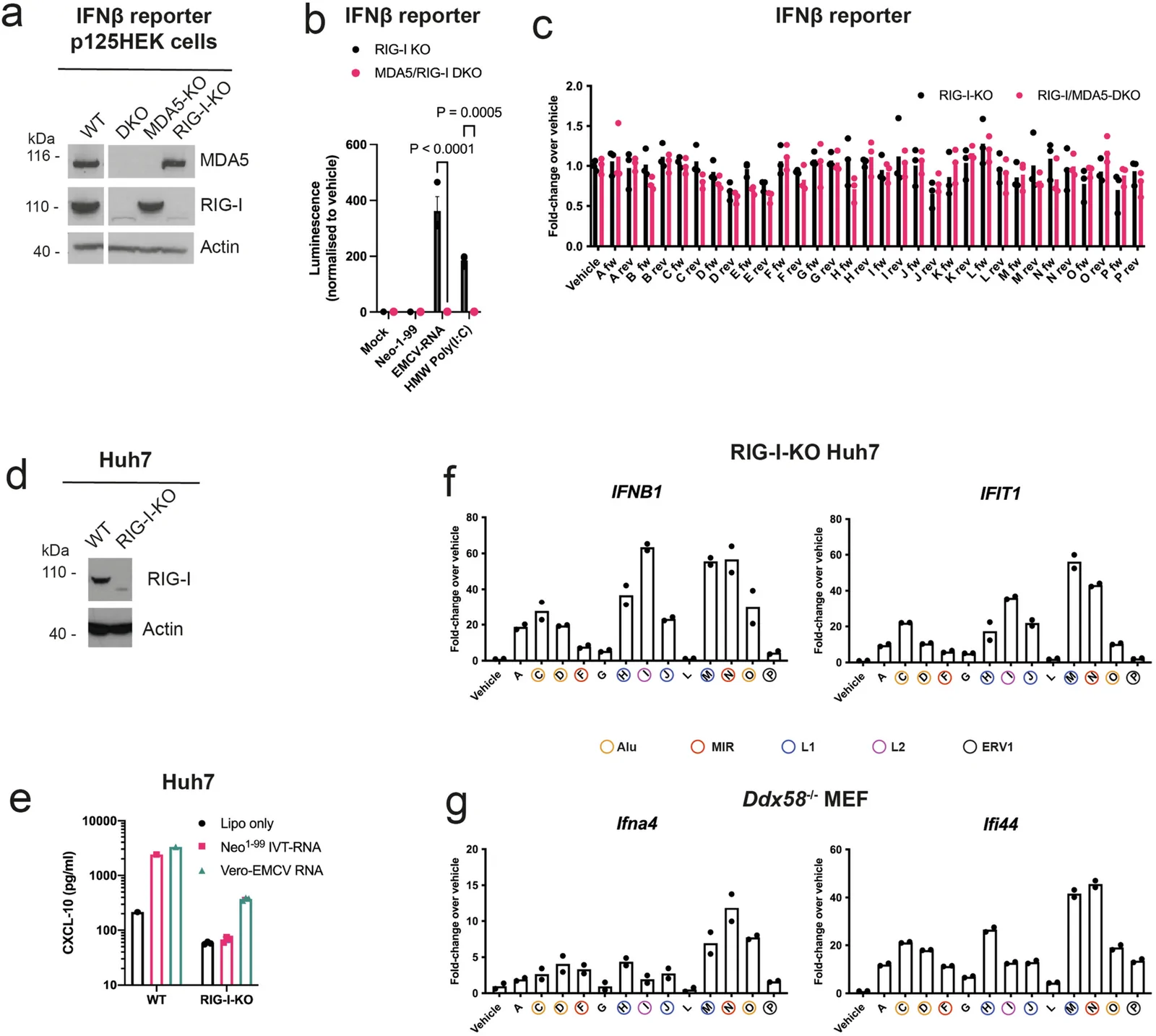
Analysis of IFN stimulation by MDA5 binding regions from cellular RNAs. **a**, p125HEK MDA5 and RIG-I double knockout (DKO) cells were generated using CRISPR/Cas9 gene targeting. Cells were treated with 100 U ml^−1^ IFN-A/D for 24 hr. Western blot indicates absence of both RIG-I and MDA5 in DKO cells. **b**, p125HEK RIG-I-KO and RIG-I/MDA5-DKO cells were transfected with Neo1–99 IVT-RNA, CIP-treated RNA from EMCV-infected HeLa cells (HeLa-EMCV-CIP RNA), HMW Poly(I:C) or were treated with transfection reagent alone (mock). After overnight incubation, luciferase activity in cell lysates was measured. Data are from three independent experimental repeats, and represent the mean +/- s.e.m. **c**, Single-stranded IVT-RNAs were transfected into RIG-I-KO or RIG-I-KO/MDA5-KO p125HEK IFNB1 reporter cells. As negative controls, p125HEK cells were treated with transfection reagent alone (Vehicle). After overnight incubation, luciferase activity in cell lysates was measured. Data from untreated cells were set to 1. **d**, RIG-I-KO Huh7 cells were generated using CRISPR/Cas9 gene targeting. Western blot indicated absence of RIG-I protein. **e**, RIG-I-KO Huh7 cells were transfected with Neo1–99 IVT-RNA, RNA from EMCV-infected Vero cells (Vero-EMCV RNA), or were treated with transfection reagent alone (Vehicle). After 24 hr, CXCL-10 in the cell culture supernatant was measured by ELISA. RIG-I-KO Huh7 cells (**f**) and *Ddx58-/-* MEFs (**g**) were transfected with the indicated IVT-dsRNAs or were treated with transfection reagent alone (Vehicle). After overnight incubation, RNA was extracted and RT-qPCR analysis was performed for *IFNB1* and *IFIT1* (Huh7), or *Ifna4* and *Ifi44* (MEF). Data are shown as fold change over vehicle relative to *GAPDH*. IVT-dsRNAs containing repeat elements are indicated (see also Tab. S1). All data except (**b**) are from single experiments. Data points in (**c**) and (**e**-**g**) represent technical replicates. Source data

To validate that host RNAs containing MDA5 binding sites can activate MDA5 in cells, we generated by in vitro transcription (IVT) ~500-nt-long RNAs from forward and reverse genomic sequences containing MDA5 binding sites (Extended Data Fig. 5a,b). High-scoring binding sites from EMCV-infected cells were selected (Tab S1). The ~500-nt length was chosen based on previous reports of the ideal dsRNA lengths for MDA5 activation^36,57^. These IVT-RNAs contained the MDA5 binding sites centrally, were annealed to form dsRNAs and were then transfected into RIG-I-deficient cells to prevent RIG-I stimulation by 5′-triphosphate groups on RNAs produced by IVT^29^. Some of the dsRNA containing MDA5 binding sites induced activation in IFN-reporter cells that were RIG-I-KO, but not in RIG-I/MDA5-double KO (DKO) cells, indicating that these regions induced an MDA5-specific activation (Fig. 3a). Comparatively, RIG-I-KO cells responded strongly to RNA from EMCV-infected cells and to Poly(I:C), but not to the RIG-I ligand Neo^(1-99)^ single-stranded IVT-RNA (Extended Data Fig. 6a,b). Transfection of single-stranded IVT-RNAs did not stimulate cells (Extended Data Fig. 6c). In both RIG-I-KO Huh7 cells (Extended Data Fig. 6d,e) and RIG-I-KO mouse embryonic fibroblasts (MEFs), a range of T1-IFN and ISG mRNA induction was observed, suggesting that some regions were more stimulatory than others (Extended Data Fig. 6f,g). For example, region L showed low stimulation, whereas regions M and N induced strong T1-IFN and ISG responses (Fig. 3a–e). Notably, unlike region L, regions M and N encompassed repetitive sequences (LINE1 and MIR, respectively). Of the 13 regions tested, ten contained repetitive sequences (Supplementary Table 1) and the corresponding IVT-dsRNAs tended to induce stronger T1-IFN responses than the remaining IVT-dsRNAs (Extended Data Fig. 6f,g and Fig. 3a–e). To test the contribution of repetitive sequences and the discovered motifs to activation of MDA5, we generated additional dsRNAs from IVT sequences M and N. The MIR repetitive region or the PolyU+GGUU/AACC motif in region N were removed, and the GGUU/AACC motif in region M was removed (Extended Data Fig. 5c,d). There was a trend toward reduced stimulatory activity when the MIR was removed from N, but not when the motifs were removed from M or N (Fig. 3a). We also selected MDA5 binding sites identified in uninfected cells and generated and tested IVT-dsRNAs (Extended Data Fig. 5b). These showed varying potency to activate MDA5 (Fig. 3e) that were similar to IVT-dsRNAs representing binding sites from EMCV-infected cells. Taken together, some of the host RNA regions that bound MDA5 induced an MDA5-dependent T1-IFN response when re-introduced as dsRNAs into uninfected cells.

### Cellular RNAs associate with MDA5 during SARS-CoV-2 infection

**Extended Data Fig. 7:**
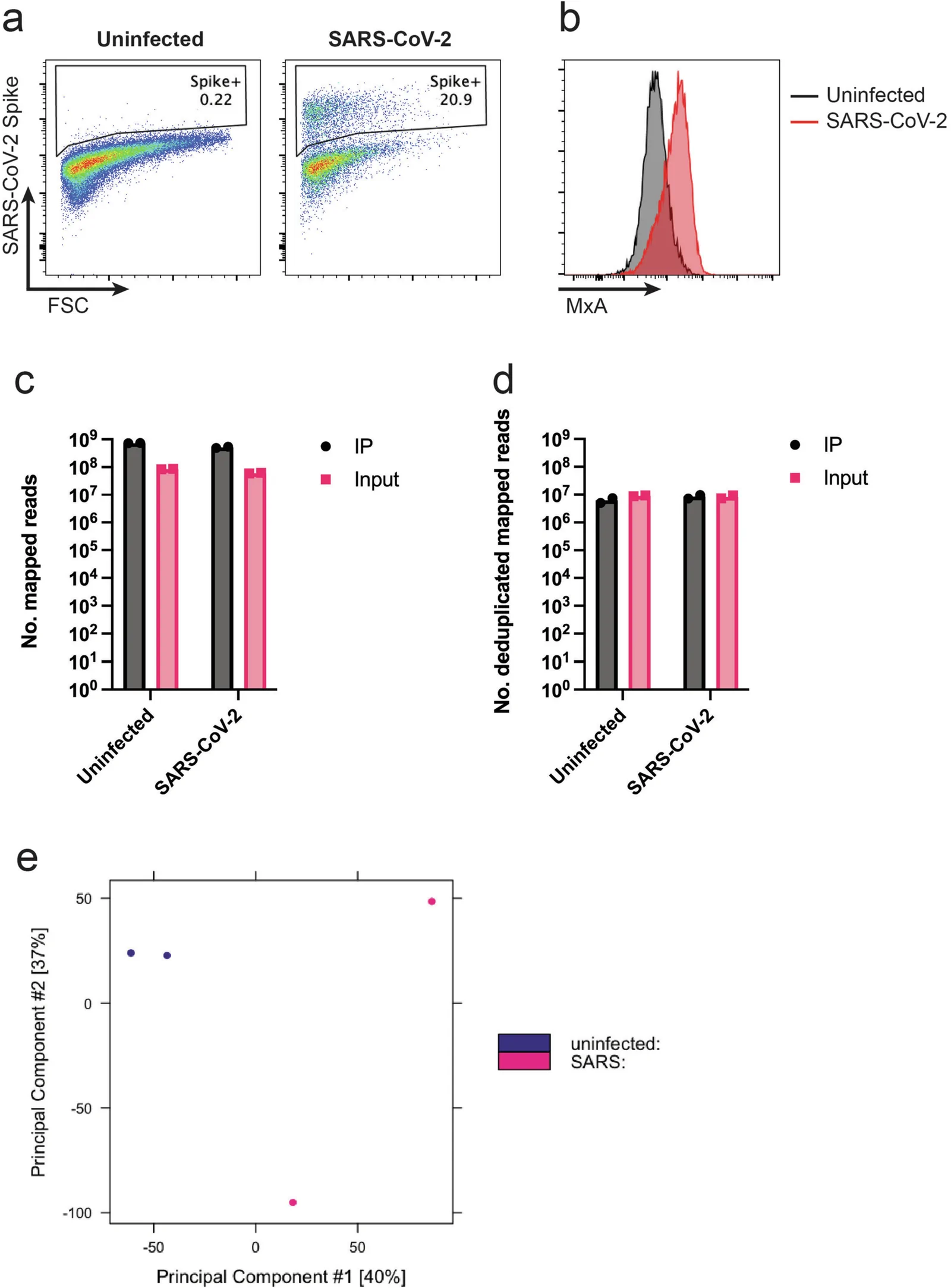
SARS-CoV-2 infectivity in Calu-3 cells and mapping rates of MDA5 iCLIP sequencing reads. **a-b**, Number of iCLIP sequencing reads that mapped to the combined human and SARS-CoV-2 genome before (**a**) and after (**b**) removal of PCR duplicates. Data points represent two independent biological repeats and bars represent the mean. **c-d**, Calu-3 cells uninfected or infected for 48 h (MOI = 0.1) with SARS-CoV-2. Cells were stained for SARS-CoV-2 Spike (**c**) and MxA (**d**) and analyzed by flow cytometry. Data shown are representative of two independent experiments. FSC = forward scatter. **e**, Principal component analysis (PCA) of MDA5 iCLIP binding sites from uninfected or SARS-CoV-2-infected Calu-3 cells. Data points are from two independent biological repeats. Source data

We and others previously reported that SARS-CoV-2 infection activates MDA5 (refs. ^9–12^). To identify MDA5 agonists during SARS-CoV-2 infection, we used Calu-3 cells, an adenocarcinoma-derived lung epithelial cell line (Extended Data Fig. 7a,b). We performed iCLIP with dual MDA5 IP as described above with some methodological improvements to enrich for bound RNA (detailed in methods). We obtained ~10^7^ uniquely mapped, deduplicated reads per sample (SARS-CoV-2-infected or uninfected cells; two biological replicates; Extended Data Fig. 7c–e).

**Fig. 4:**
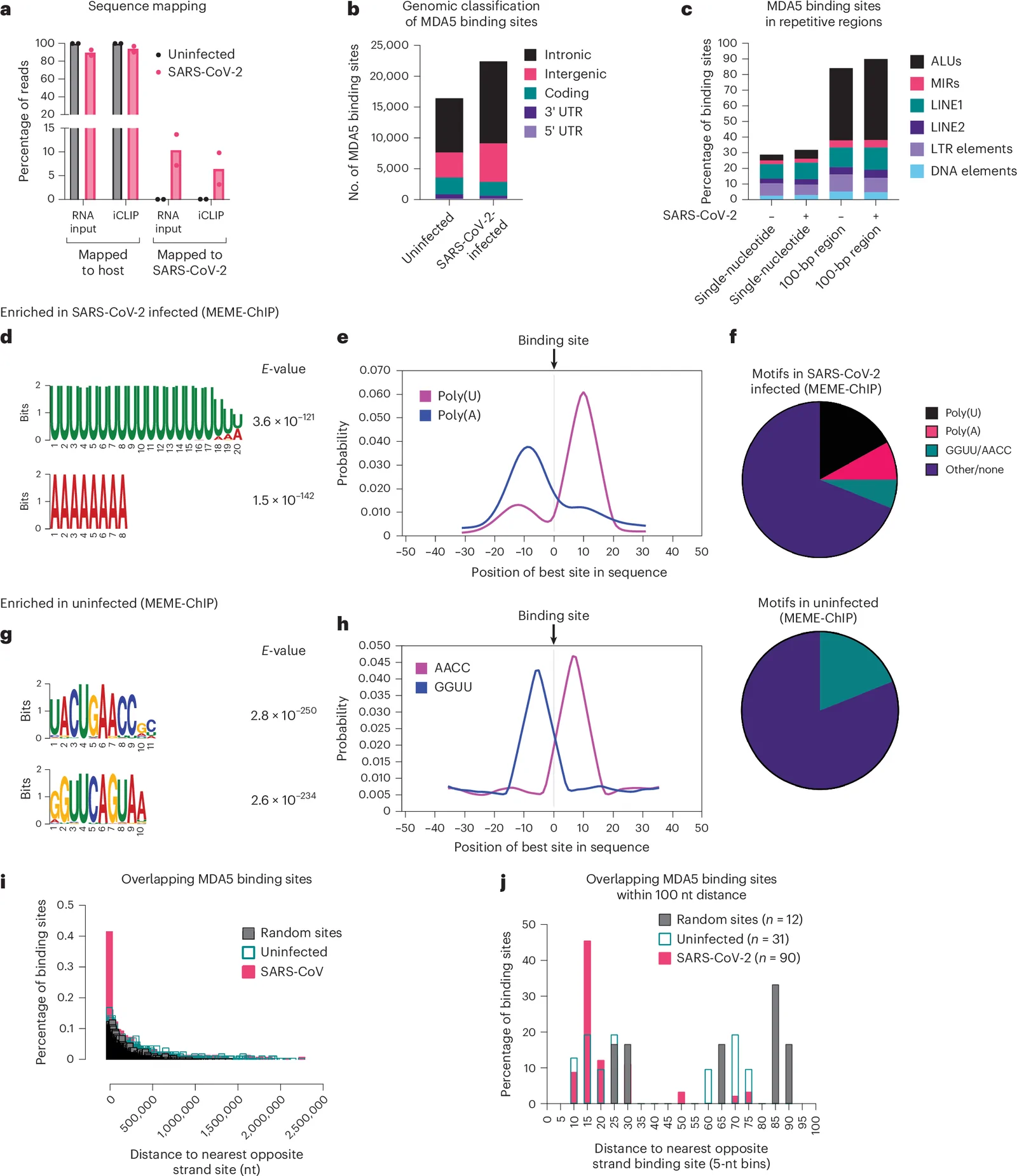
MDA5 binds to host RNA during SARS-CoV-2 virus infection. **a**, Proportion of RNA input or MDA5 iCLIP samples mapping to the host or the SARS-CoV-2 genome. Symbols represent data from two independent biological repeats. **b**, Genomic classification of single-nucleotide MDA5 binding sites detected by iCLIP. **c**, Proportion of single-nucleotide MDA5 binding sites, or 101-nt regions (50 nt upstream and downstream of single-nucleotide binding site) that are located within repetitive regions of the human genome. **d**–**h**,101-nt regions around MDA5 binding sites, as described in **c**, were analyzed for de novo RNA motifs using MEME-ChIP. Specific motifs enriched in SARS-CoV-2-infected samples compared to uninfected samples (**d**,**e**), and in uninfected samples (**g**,**h**), as detected by MEME-ChIP. Associated *E*-values (adjusted *P* value multiplied by the number of motifs in the input file) are shown. Localization of the motifs discovered by MEME-ChIP relative to the crosslink site (position 0, black arrow) are shown in **e**,**h**. Proportion of motifs in MDA5 binding sites from uninfected or SARS-CoV-2-infected cells is shown in **f**. **i**, The distance between all MDA5 binding sites from SARS-CoV-2-infected samples or uninfected samples to their nearest binding site in the opposite strand were calculated and compared to randomly generated sites across the genome. **j**, As **i** but depicting the number of sites within 100 nt of each other. Data points in **a** represent two independent biological repeats, and bars/error bars represent the mean ± s.d. Analyses in **b**–**j** were conducted on combined data from two independent biological repeats. Source data

Approximately 10% of RNA sequences in the SARS-CoV-2-infected RNA input samples mapped to the SARS-CoV-2 genome (Fig. 4a); however, there was no enrichment in SARS-CoV-2 RNA in the MDA5 IP samples (Fig. 4a). We did not detect MDA5 binding sites in viral RNA; instead, MDA5 bound only host RNA. Over 50% of binding sites were found in introns (Fig. 4b), followed by intergenic regions (~25%) and protein coding regions (~10%). Furthermore, around 30% of binding sites were located within repetitive regions (Fig. 4c). When we extended our analysis to 101-nt regions spanning binding sites, ~50% of these regions overlapped with an *Alu* repeat, without major increases in any of the other repeat categories. Thus, in agreement with the findings for EMCV infection, we observed that during SARS-CoV-2 infection, MDA5 binds host, rather than viral, RNA. This host RNA is mostly intronic and enriched in *Alu* repeats close to the MDA5 binding site.

We next analyzed the same 101-nt regions containing MDA5 binding sites using motif-finding algorithms. Poly(U) and Poly(A) motifs were enriched in SARS-CoV-2-infected samples (Fig. 4d–f and Extended Data Fig. 4d–f). These motifs were approximately 10 nt away from MDA5 binding sites (Fig. 4e). Motifs containing AACC or GGUU sequences were identified in both uninfected and SARS-CoV-2-infected samples at a distance of approximately 10 nt from MDA5 binding sites (Fig. 4f–h and Extended Data Fig. 4d–f); however, these were not enriched in infected cells when compared to uninfected cells. Given that the rate of infection was ~20% (Extended Data Fig. 7a), uninfected cells possibly contributed to the presence of the AACC/GGUU motifs in the infected sample. This suggests that MDA5 binds AACC/GGUU motifs in uninfected cells. In sum, similar sequence motifs were enriched near MDA5 binding sites in both the SARS-CoV-2 and EMCV datasets.

As base-pairing (resulting in the formation of ‘dsRNA-like’ stretches) can occur between Poly(A) and Poly(U) motifs, we analyzed the proximity between identified motifs, to determine whether inter- or intra-strand base-pairing was possible. We found that of the 4,579 Poly(A) and Poly(U) motifs identified by MEME-ChIP in the SARS-CoV-2-infected samples, 259 (5.7%) occurred within 200 nt of another complementary motif, either on the same or opposite strand. Of 869 AACC/GGUU motifs found in the uninfected samples, 154 (18%) occurred within 200 nt of another complementary motif on the opposite strand, whereas only two were close to a complementary motif on the same strand, suggesting inter-strand complementarity. We also investigated the potential for bidirectional transcription that may have occurred at MDA5 binding sites, potentially generating dsRNA species. We defined ‘overlapping’ binding sites as any two binding sites within 100 nt of each other on opposite strands and compared this to randomly generated sites across the genome. There was no difference in the number of overlapping binding sites in uninfected cells compared to random control, but 0.4% of binding sites from SARS-CoV-2-infected cells were within 100 nt of another site on the opposite strand, compared to 0.1% in randomly generated sites, suggesting a small proportion of MDA5 binding sites in infected cells may have been generated from bidirectional transcription (Fig. 4i). Of note, when we further investigated these closely occurring MDA5 binding sites, we found that 41 of 90 (45%) occurred between 10–15 nt from each other (Fig. 4j). Given the ‘footprint’ of MDA5 on dsRNA is estimated to be 14–15 nt (ref. ^56^), we speculate that these binding sites could represent nucleotides crosslinked at both edges of a single MDA5 protein bound to a segment of dsRNA. Combined, our analysis of the RNAs bound by MDA5 during SARS-CoV-2 infection found enrichment in sequences with the potential to form intra- and inter-strand dsRNA structures, which may constitute cellular ligands for MDA5.

### Aberrant RNAs are increased in the cytoplasm of virus-infected cells

**Fig. 5:**
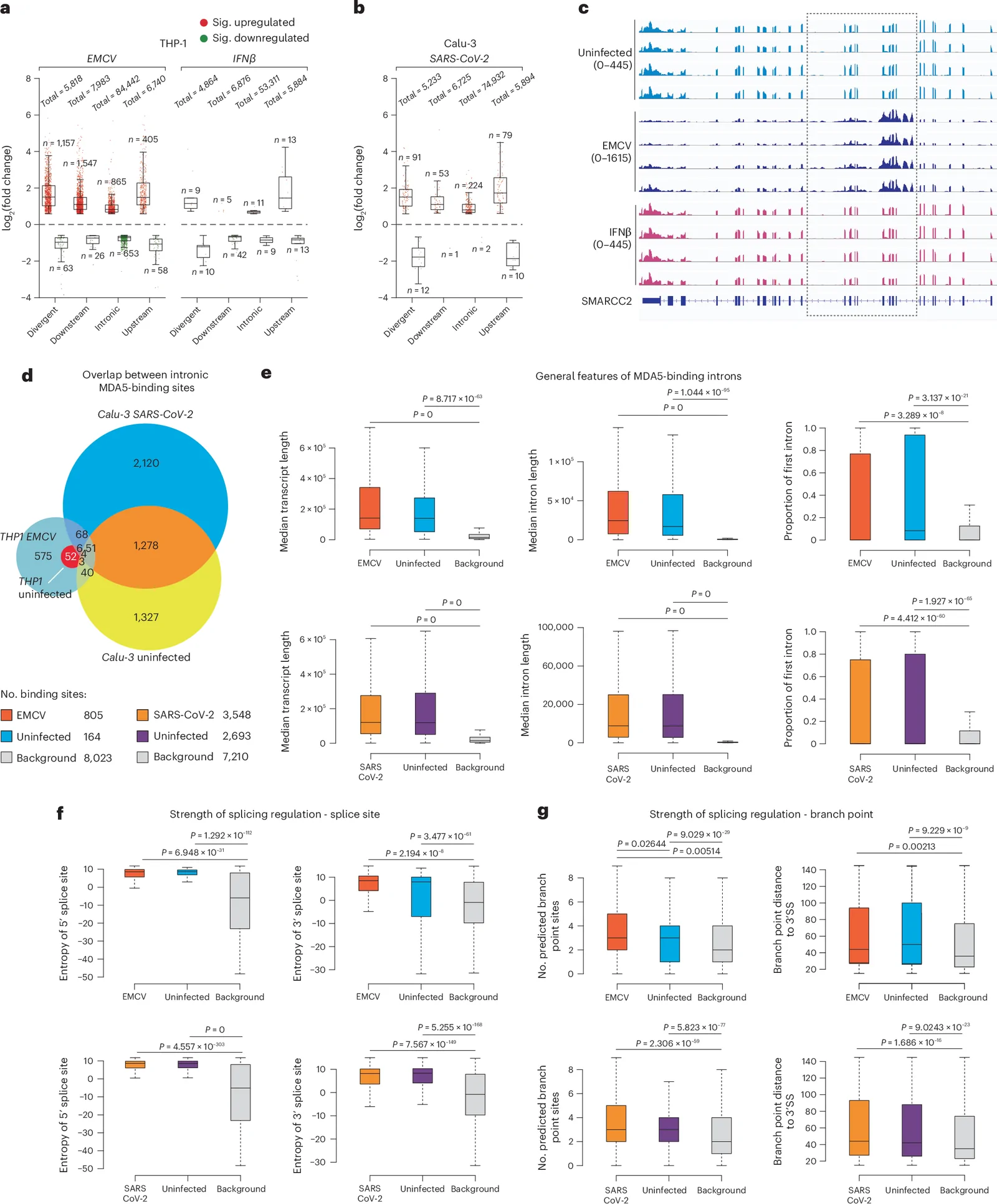
Virus infection induces aberrant transcription, and MDA5-bound intronic RNAs are predisposed to inappropriate splicing. **a**, MDA5-WT THP1 cells were left untreated, infected with EMCV (MOI = 2) for 20 h, or treated with IFNβ (100 U ml^−1^) for 6 h (EMCV and untreated, *n* = 4 independent biological repeats; IFNβ, *n* = 3 independent biological repeats). RNA was extracted from the cytoplasmic fractions, rRNA-depleted and sequenced. Transcription levels in the indicated regions were normalized to matched constitutive exons and compared to mock controls. Only significant changes (log_2_(fold change) ≥ 0.585, *P*adj ≤ 0.01) are shown, with the total number of tested regions indicated at the top. The plot central line shows the median, and the box represents the 25th to 75th percentile range. Whiskers extend to the lowest and highest values within 1.5× the interquartile range. **b**, Calu-3 cells were left untreated or infected with SARS-CoV-2 (MOI = 0.1) for 48 h (*n* = 4 independent biological repeats) and analyzed as in **a**. **c**, Exemplar RNA sequencing tracks from **a**, with reads from *SMARCC2* in individual biological replicates shown. Region showing increased intronic reads in EMCV-infected cells are highlighted in dashed-line box. **d–g**, Intronic MDA5 binding sites were extracted from iCLIP datasets (THP1 cells ± EMCV (*n* = 4 independent biological repeats) and Calu-3 cells ± SARS-CoV-2 (*n* = 2 independent biological repeats)) and analyzed for: (**d**) introns in common between conditions; (**e**) strength of splice site (entropy at 5’ and 3’ sites); (**f**) strength of splicing regulation at the branch point (number of branch points and distance to 3’ splice site); and (**g**) general features (transcript length, intron length, and proportion of sites in first introns). Data in **d–g** are compared to an equivalent background control. In **e**–**g**, the center line represents the median, the box spans the interquartile range (25th to 75th percentile) and the whiskers extend to the minimum and maximum values. Statistical analysis performed using Mann–Whitney *U*-test. Source data

As we found that many MDA5 binding sites were in introns and intergenic regions (Figs. 2b and 4b), we next tested whether virus infection itself affected the presence of introns and other non-canonical RNA species in the cytoplasm, where MDA5 is localized. We extracted and sequenced cytoplasmic RNA from THP1 and Calu-3 cells infected with EMCV or SARS-CoV-2, respectively, or uninfected controls. As an additional control, we treated cells with 100 U ml^−1^ IFNβ to assess the effects of transcriptional changes induced by the T1-IFN response. We assessed aberrant transcription events, defined as intronic transcripts, transcripts upstream or downstream of known genes, and divergent transcription^58^. Downstream transcription can include defective transcription termination, where the transcription continues beyond the transcript end due to failed 3′ end processing^59^. We found that both EMCV infection in THP1 cells and SARS-CoV-2 infection in Calu-3 cells induced significant occurrence of aberrant transcription events (Fig. 5a,b). In comparison, treatment of cells with IFNβ caused very few such events in THP1 cells (Fig. 5a). An example is provided in Fig. 5c, where EMCV infection but not IFNβ treatment led to accumulation of intronic RNA from the *SMARCC2* gene.

**Extended Data Fig. 8:**
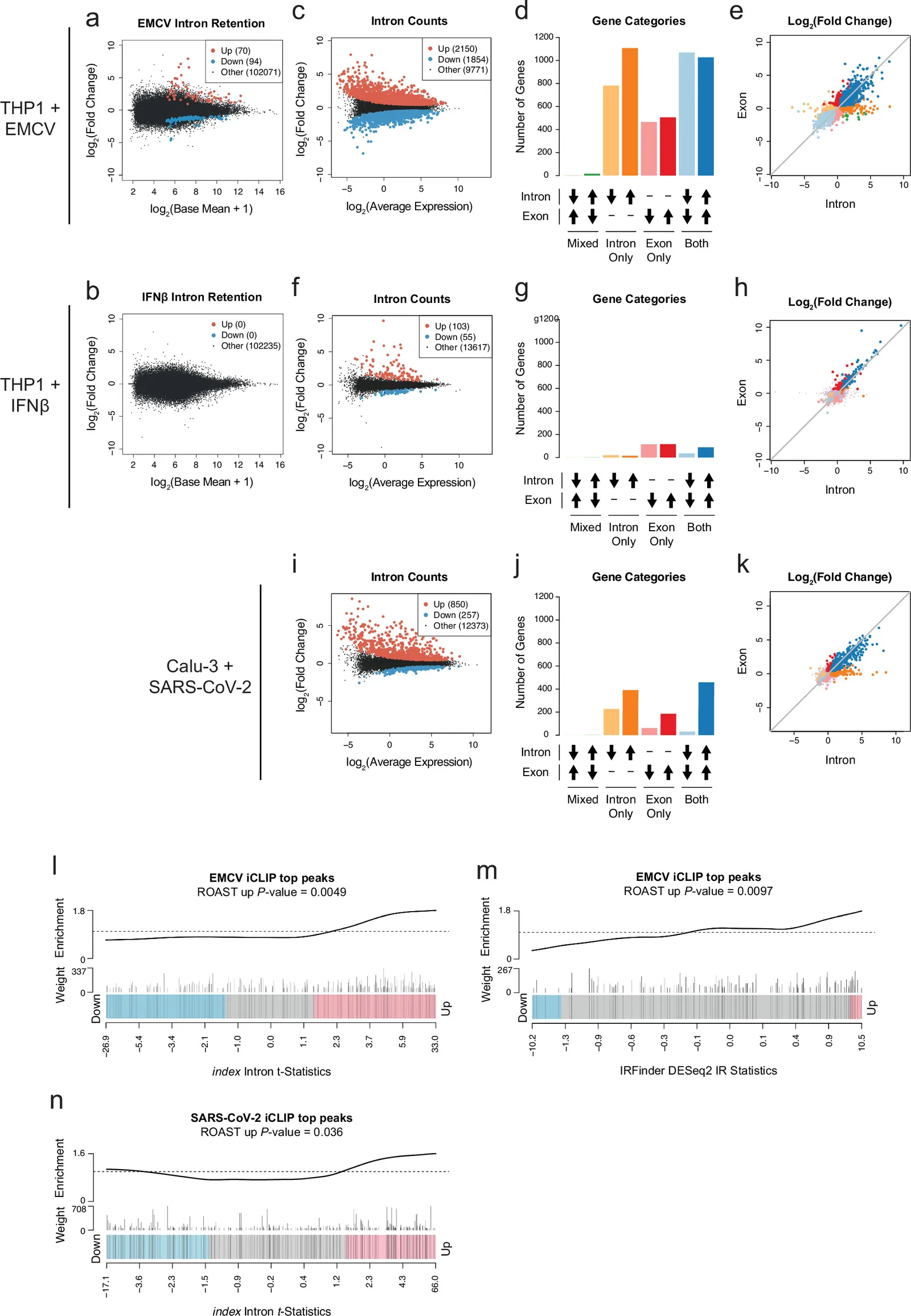
Virus infection upregulates introns in the cytoplasm, which are enriched for MDA5 binding sites. **a**-**h**, THP1 MDA5-WT cells were left untreated, infected with EMCV (MOI = 2) for 20 h, or treated with IFNβ (100 U ml^−1^) for 6 h (EMCV and untreated, *n* = 4 independent biological repeats; IFNβ, *n* = 3). RNA was extracted from the cytoplasmic fractions, rRNA-depleted and sequenced. Intron retention determined by IRFinder analysis on EMCV-infected cells (**a**) and IFNβ-treated cells (**b**) compared to untreated cells, with upregulated (red) and downregulated (blue) intron regions compared to surrounding exon regions highlighted. Differential expression of genes based on intronic read counts determined by index analysis in EMCV-infected cells (**c**) and IFNβ-treated cells (**f**) compared to untreated cells, with upregulated (red) and downregulated (blue) genes highlighted. Comparison of differential gene expression based on intronic and exonic read counts in EMCV-infected cells (**d**, **e**) and IFNβ-treated cells (**g**, **h**) compared to untreated cells. The number of genes up- or downregulated on an intronic level, an exonic level or both are indicated. **i-k**, Calu-3 cells were left untreated or infected with SARS-CoV-2 (MOI = 0.1) for 48 h (*n* = 4 independent biological repeats) and the analysis shown in (**a**-**h**) was repeated. **l-n**, Barcode plots showing the association of EMCV iCLIP peaks with intron retention by IRFinder analysis (**l**) or index analysis (**m**), and association of SARS-CoV-2 iCLIP peaks with index analysis (**n**). Vertical lines indicate introns that overlapped with the most highly scoring intronic iCLIP peaks (score > 95th percentile), weighted by iCLIP score and arranged according to differential expression statistics (significantly downregulated and upregulated genes are shaded in blue and red, respectively). The overall enrichment is indicated by the enrichment line above, and significance was calculated using ROAST. All sequencing data is from three or four independent biological repeats. Source data

In an alternative approach focusing on cytoplasmic introns specifically, we analyzed changes in the abundance of intronic sequences in the cytoplasm using two methods: IRFinder^60^ and index^61^. IRFinder identifies introns retained within otherwise canonically spliced messenger RNA (most of the introns are normally spliced, but one or more introns may have been retained), whereas index examines the overall level of intron reads on a per-gene basis, which can cover both intron retention and unspliced pre-mRNA^62^. Analysis with IRFinder showed a modest amount of intron retention after EMCV infection, but not after IFNβ treatment, compared to untreated cells (Extended Data Fig. 8a,b). Index showed that both EMCV and SARS-CoV-2 infection had a significant effect on intron expression in the cytoplasm, which was much greater than the effect of IFNβ treatment (Extended Data Fig. 8c–k). Indeed, compared to untreated cells, virus infection specifically induced an upregulation in introns in the cytoplasm (Extended Data Fig. 8d,j), which was not evident in cells treated with IFNβ (Extended Data Fig. 8g). Of note, there was a significant positive correlation between the introns upregulated/retained in the cytoplasm of virus-infected cells with introns that contained MDA5 binding sites detected by iCLIP (Extended Data Fig. 8l–n), where introns that were upregulated in the cytoplasm were more likely to also overlap with introns bound by MDA5 in our iCLIP data. Thus, we found that viral infection causes significant changes to the levels of introns in the cytoplasm. These upregulated introns were also associated with MDA5 binding during virus infection, suggesting that they stimulate MDA5.

We further analyzed the nature of introns bound by MDA5 in our iCLIP datasets. Although there was minimum overlap between the introns bound by MDA5 in THP1 cells and Calu-3 cells (Fig. 5d) these shared similar features. A high proportion of intronic MDA5 binding sites were in the first intron of a gene and tended to be longer introns (Fig. 5e). It has been demonstrated that long introns have a higher predisposition to induce premature transcription termination, which generates incomplete pre-mRNAs harboring introns, and these have been suggested to form stimulatory dsRNAs^63^. Moreover, introns bound by MDA5 had weaker splice sites (denoted by higher splice site entropy; Fig. 5f) and sub-optimal branch point sites (increased branch point number and distance to 3’ splice site; Fig. 5g). These intronic features can result in reduced splicing, as both weaker splice sites and suboptimal branch points reduce recognition by the spliceosome machinery at the *cis*-regulatory sequences within the pre-mRNA^62,64^. Together, these findings support the concept that infection, at least with the viruses tested here, induces aberrant RNA transcription and/or processing, particularly affecting splicing of pre-mRNAs that are prone to reduced splicing due to for example weak splice sites. The resulting aberrant long intron-containing RNAs are then bound by MDA5.

### Reduction of aberrant transcripts during infection abrogates MDA5 activation

**Fig. 6:**
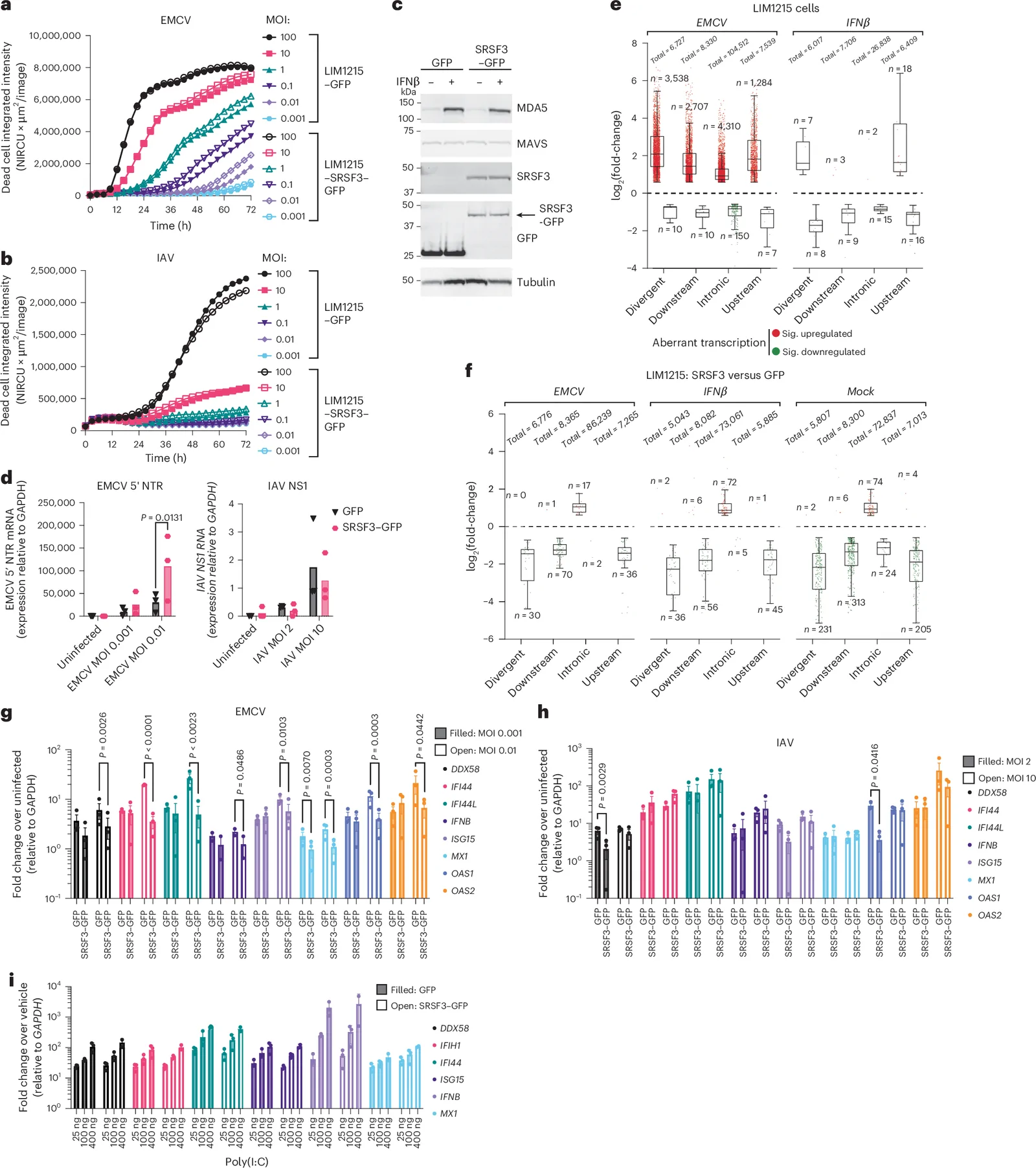
Overexpression of the splicing factor SRSF3 prevents MDA5 activation during EMCV infection. **a**,**b**, LIM1215 cells stably overexpressing either GFP or SRSF3–GFP (LIM1215–GFP and LIM1215–SRSF3–GFP) were infected with (**a**) EMCV or (**b**) IAV with the indicated MOIs for 72 h, and cell viability was assessed by Incucyte. NIRCU, near infrared calibration units. Data in **a**,**b** are representative of three independent biological repeats. **c**, MDA5, MAVS and GFP protein levels in LIM1215–GFP and LIM1215–SRSF3–GFP, untreated or treated with IFNβ (100 U ml^−1^) for 24 h, were analyzed by western blot. Tubulin served as a loading control. Data in **c** is from a single experiment. **d**, LIM1215–GFP or LIM1215–SRSF3–GFP were infected with EMCV (MOI = 0.001 and 0.01) or IAV (MOI = 2 and 10) for 48 h. RNA was extracted and RT–qPCR was performed for the EMCV 5’ nontranslated region (NTR) and IAV NS1. Data are shown relative to *GAPDH*, and are representative of three independent biological repeats. **e,f**, LIM1215–GFP or LIM1215–SRSF3–GFP were infected with EMCV (MOI = 0.01) for 48 h, RNA was extracted from the cytoplasmic fractions, rRNA-depleted and sequenced (*n* = 4 independent biological repeats. Aberrant transcription levels in the indicated regions were normalized to matched constitutive exons and compared to mock controls. Only significant changes (log_2_(fold change) ≥ 0.585, *P*adj ≤ 0.01) are displayed, with the total number of tested regions indicated at the top. The plot central line shows the median, and the box represents the 25th to 75th percentile range. Whiskers extend to the lowest and highest values within 1.5× the interquartile range. **g**,**h**, RNA from **d** was assessed for expression of indicated mRNAs by RT–qPCR. Data shown is fold change relative to *GAPDH*, and is from three independent biological repeats. Data are presented as mean ± s.e.m. **i**, LIM1215–GFP or LIM1215–SRSF3–GFP were transfected with 25, 100 or 400 ng ml^−1^ of Poly(I:C) for 16 hr. RNA was extracted and RT–qPCR performed for the indicated mRNAs. Data are shown are fold change relative to *GAPDH*, and is from three independent biological repeats. Data are presented as mean ± s.e.m. Statistical analysis in **d** and **g–i** was by two-way ANOVA with Šídák multiple comparisons test. Source data

Several viruses that activate MDA5 also modulate the subcellular distribution of splicing factors, such as SRSF3 (refs. ^65,66^), and nuclear ribonucleoproteins, such as hnRNPC^67,68^, thus significantly affecting host RNA metabolism^69^. Moreover, SARS-CoV-2 infection disrupts host RNA splicing, resulting in intron retention^70^. We therefore hypothesized that some viral infections lead to an imbalance in host RNA processing and metabolism, resulting in an increase of aberrant RNAs in the cytoplasm that activate MDA5. To test this idea, we aimed to restore RNA homeostasis by overexpressing SRSF3 (also known as SRp20), a global regulator of pre-mRNA splicing and mRNA export^71^, which was previously shown to be targeted by poliovirus, a picornavirus that activates MDA5 (ref. ^65^). IAV is known to activate a T1-IFN response via RIG-I, and not MDA5, providing a control for MDA5-specific effects^7^. SRSF3–GFP-overexpressing LIM1215 cells and GFP-overexpressing control cells^72^ (respectively termed LIM1215–SRSF3–GFP and LIM1215–GFP) were equally susceptible to EMCV- and IAV-induced cell death (Fig. 6a,b), and expressed equivalent levels of MDA5 and MAVS (Fig. 6c). Viral replication was not hindered in LIM1215–SRSF3–GFP cells compared to control (Fig. 6d). Of note, we measured IP-10 in cell culture supernatant as a readout of cytokine responses but found that LIM1215 cells did not release significant IP-10 in response to viral infections.

Similar to what was observed with THP1 cells, EMCV infection of LIM1215 cells induced significant aberrant transcription (Fig. 6e). Overexpression of SRSF3–GFP in LIM1215 cells reduced aberrant RNA transcription and processing in cells (Fig. 6f). At viral doses that did not result in cell death, the T1-IFN response to EMCV was blunted in LIM1215–SRSF3–GFP cells compared to control cells (Fig. 6g), but this effect was not seen with IAV infection (Fig. 6h) or in response to Poly(I:C) (Fig. 6i). This lower response to EMCV was not simply due to lower infection levels in LIM1215–SRSF3–GFP cells, as equivalent or increased viral RNA levels were present in LIM1215–SRSF3–GFP cells compared to control cells (Fig. 6d). These observations suggest that when RNA homeostasis is rebalanced despite virus infection, then MDA5 activation is compromized. Taken together, our data indicate that improperly processed cellular RNAs, including cytoplasmic introns, accumulate during virus infections and activate MDA5, rather than viral dsRNAs.

## Discussion

**Extended Data Fig. 9:**
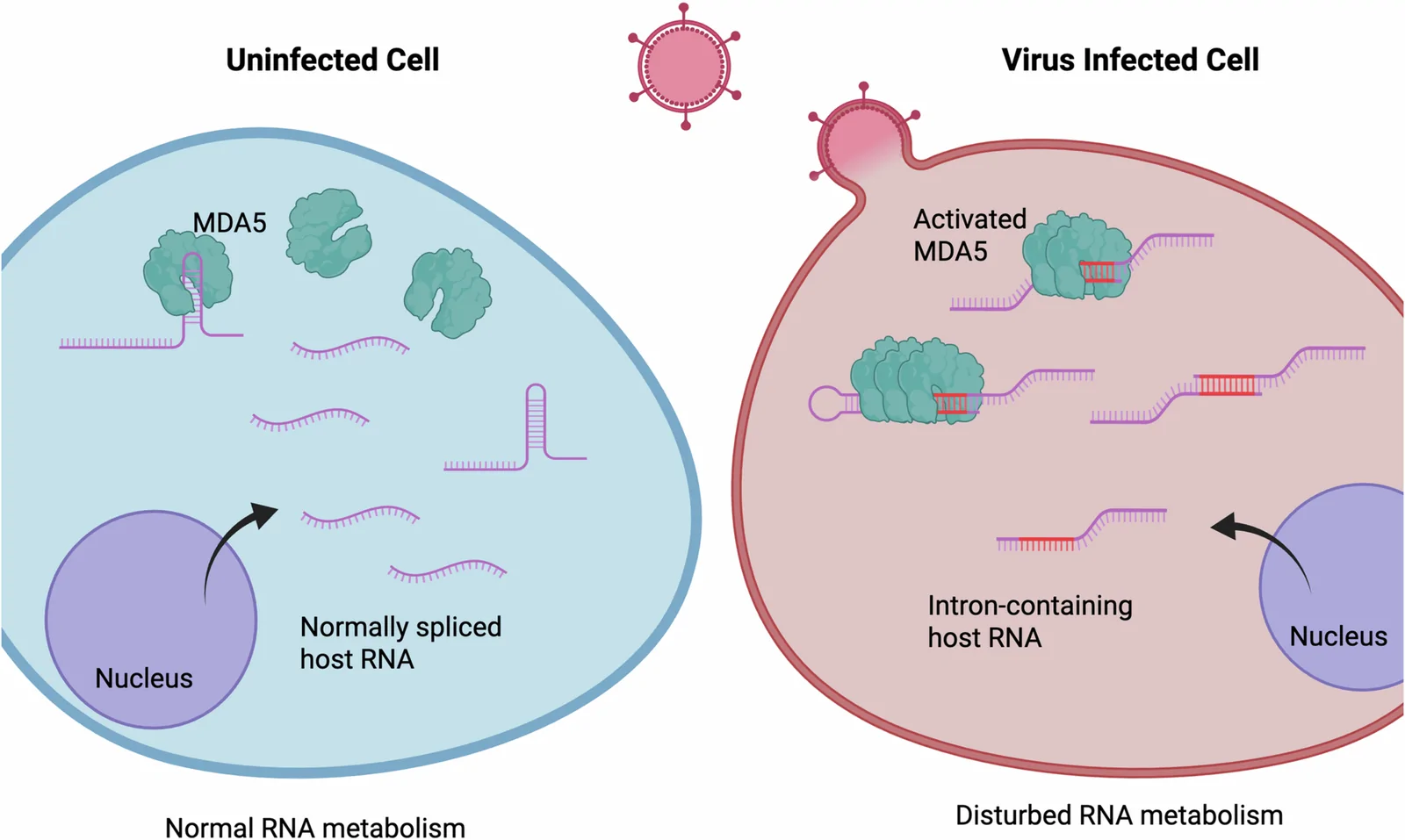
Graphical abstract. Created in BioRender. Hertzog, P. (2026) https://BioRender.com/a54mmeh.

MDA5 is a notable PRR^8^. Despite its discovery as an RNA sensor of virus infection over 20 years ago^34^, the PAMP detected by MDA5 during infection has remained obscure and controversial, rendering MDA5 one of the few remaining ‘orphan’ PRRs. Here, we propose that MDA5 monitors the homeostasis of cellular RNA processing and can be activated upon perturbations caused by infections (Extended Data Fig. 9). Conceptually, this finding can be described as ‘guarding’, which involves the detection of pathogen-induced perturbations rather than PAMPs. Several plant immune receptors and the mammalian Pyrin inflammasome employ this mechanism; for example, the latter detects bacterial toxin-induced Rho guanosine triphosphatase inactivation^73^. We found that defects in RNA processing in virally infected cells triggered MDA5 via recognition of cellular intronic sequences accumulating in the cytoplasm. Our work thus proposes innate immune guarding as a potential mechanism underpinning cytoplasmic RNA sensing during virus infection.

This finding was unexpected as MDA5 is often described as a PAMP sensor of viral dsRNA accumulating in infected cells. However, our observations were consistent between two different cell types, monocytic THP1 cells and lung adenocarcinoma Calu-3 cells, and two virus infections, EMCV and SARS-CoV-2. We cannot exclude the possibility that MDA5 detects viral dsRNAs in different settings such as in cells infected with other or defective viruses, in other cell types or at different time points during infection. Nonetheless, our interpretation (in which MDA5 guards posttranscriptional control) provides an attractive explanation as to why MDA5 can detect many different infections that are unlikely to all generate the same PAMP. Moreover, viral dsRNAs are often associated with viral RNA binding proteins and, for many viruses, replication takes place in replication factories. For example, SARS-CoV-2 establishes double membrane vesicles, in which its polymerase produces new viral RNA^74^. As such, how MDA5 could gain access to viral dsRNA is questionable, in particular given that in vitro data suggest multiple MDA5 molecules need to bind the same RNA for activation^36^. Moreover, even if MDA5 were to gain access to viral dsRNA in replication factories, it is difficult to envisage how MDA5 would then be able to interact with mitochondrially localized MAVS^75^. Our findings resolve these conundrums: detection of aberrant host RNAs explains how MDA5 can sense viral infections when viral RNAs are shielded by viral proteins or within replication factories.

How do viruses perturb the processing of cellular RNAs? Some viruses, including SARS-CoV-2 (ref. ^70^), encode antagonists that directly inhibit splicing and induce intron retention, presumably to re-program the cell toward viral gene expression^76^. Dengue virus^77,78^, poliovirus^65,66,68^ and rhinovirus^79,80^ also affect host RNA homeostasis by causing cytoplasmic relocalization of splicing and nuclear RNA binding factors. Furthermore, MDA5 is activated by DNA virus infections, including by herpes simplex virus-1, vaccinia virus and hepatitis B virus^13,14,39^. DNA viruses do not generate a replicative form viral dsRNA but interfere with splicing^76^. We posit that activation of MDA5 occurs when the fidelity of RNA processing is impacted upon virus infection, either through direct viral antagonists or perhaps also indirectly through cellular stress^76^. It will be important to test this in future work for different virus families, as will be the analysis of MDA5’s RNA agonists in non-viral infections^16–19^. Of note, it was recently suggested that unspliced RNA derived from integrated HIV-1 proviral DNA activates MDA5 (refs. ^81–83^). Although formally of viral origin, unspliced HIV-1 transcripts are generated by the cell’s transcription machinery; thus, this finding mirrors our observation that MDA5 is activated by intron-containing cellular RNAs.

We found specific sequence motifs in host RNA that were associated with MDA5, including Poly(A)/(U)-rich sequences. Structurally, MDA5 binds dsRNA in a sequence-independent manner, due to interactions with the phosphodiester backbone^36^. It is possible that the motifs discovered here are first bound by other RNA binding proteins that are then replaced by MDA5. Indeed, several RNA binding proteins specifically bind to Poly(U) tracts^84^, including some known to be relocalized to the cytoplasm during virus infection such as hnRNPC^67,85^. Thus, when viruses usurp host RNA factors for their own replication and induce cytoplasmic relocalization of nuclear factors, this may in fact result in these nuclear factors bringing with them to the cytoplasm unspliced RNAs that then activate MDA5.

Although MDA5 was first discovered as a sensor of virus infection, it is also activated in autoinflammatory disease. In the absence of ADAR1, which catalyzes the deamination of adenosines to inosines in dsRNA to break base-pairing, MDA5 detects cellular dsRNA species^86^. This leads to sterile induction of T1-IFN. Accordingly, inactivating mutations in *ADAR1* cause the T1-IFN-mediated Aicardi–Goutières syndrome, a neurodevelopmental disorder^87^. Host repetitive RNA, in particular inverted *Alu* repeats, which can form base-paired structures, have been proposed as RNA ligands of MDA5 in this condition^43^, consistent with our observation that many MDA5 binding sites are in proximity of *Alu* elements. Moreover, MDA5 was also found to be stimulated by RNA transcripts from endogenous retroviruses (ERVs) when cancer cells were treated with demethylating agents (for example azacytidine derivatives). These compounds derepress ERVs, resulting in the formation of dsRNAs that stimulate MDA5 (refs. ^88,89^). Interestingly, treatment of cancer cells with inhibitors of the spliceosome results in intron retention in cytoplasmic RNA and concomitant activation of T1-IFN via MAVS, potentially explaining the therapeutic efficacy of drugs targeting splicing^90^. Moreover, depletion of the splicing regulators hnRNPC and hnRNPM induces MDA5 activation^91–93^. These observations provide important parallel lines of evidence to the work presented here and suggest that sensing of aberrant RNA processing is a universal mechanism explaining MDA5 activation during both infections and in sterile settings. It is also noteworthy that in addition to intronic RNA other types of noncoding RNA may contribute to MDA5 activation. For example, in neuronal cell types, 3′ UTRs are extended and have been suggested to activate MDA5 due to the presence of inverted repeat *Alu* elements^94^.

A noteworthy methodological advance of our work is the use of MDA5 monoclonal antibodies that allow identification of the RNAs that bind endogenous MDA5 in situ in cells. While here applied to virus infections, our protocol can be used in any human cell type that expresses MDA5, including primary cells and patient samples. The MDA5 antibodies and iCLIP protocol are available as an open resource to the research community. We hope they will be applied in future to discover MDA5 agonists in autoinflammation and cancer treatment.

Like MDA5, other nucleic acid sensors have also been described to detect viral dsRNA. Z-DNA/RNA binding protein 1 (ZBP1), protein kinase R (PKR) and members of 2′-5′-oligoadenylate synthetase (OAS) family bind dsRNA and are activated by virus infections^95^, many of which also activate MDA5, and have been associated with non-infectious pathologies^96–100^. These receptors, rather than directly inducing a T1-IFN response, largely act to restrict virus spread by triggering cell death, global RNA degradation or shutting down mRNA translation. Interestingly, recent work showed that inhibition of splicing activates ZBP1 (refs. ^101,102^). Moreover, disruption of transcription termination caused by viruses including IAV and herpes simplex virus-1 results in abnormally long 3′ extensions of host cell mRNAs that activate ZBP1 (ref. ^58^). OAS1 was recently found to bind host intronic and repetitive RNA, in addition to viral RNA, during SARS-CoV-2 infection^103^. Whether PKR and OASs are (like MDA5) activated by host intronic RNAs in the context of different virus infections is worth investigating.

Taken together, our work shifts the understanding of MDA5 activation during infection: rather than exclusively detecting viral RNA as a PAMP, as is the case with other PRRs, we propose that MDA5 can also function as a sensor of cellular RNA homeostasis. When disturbances in this homeostasis occur as a result of virus infection, MDA5 is triggered by improperly processed cellular RNAs. These findings have important bearings on treatment approaches for both infectious and noninfectious diseases that involve MDA5 activation.

## Methods

### Cell culture and virus infection

Cells were cultured at 37 °C and 5% CO_2_ and routinely screened for mycoplasma contamination. THP1 (gift from Vincenzo Cerundolo) and LIM1215 cells were maintained in RPMI (Sigma Aldrich) supplemented with 10% v/v fetal calf serum (FCS) and 2 mM L-glutamine (Gibco). Huh7 (gift from J. McKeating), A549 (gift from G. Kochs), BHK-21 (gift from A. Townsend), p125HEK^47^ and MEF (gift from Shizou Akira) cells were maintained in DMEM supplemented with 10% v/v fetal calf serum (FCS) and 2 mM L-glutamine (Gibco). Calu-3 cells (gift from C. Goujon) were maintained in MEM (Sigma Aldrich) supplemented with 10% v/v fetal calf serum (FCS), 2 mM L-glutamine (Gibco), 1× sodium pyruvate (Gibco) and 1× non-essential amino acids (Gibco). For generation of MDA5 and RIG-I KO cells using CRISPR/Cas9 technology, sgRNAs cloned into pX458-Ruby (Addgene 110164, deposited by Philip Hublitz) and described earlier^47^ were used. LTX Transfection kit (Life Technologies) was used to transfect 2 × 10^6^ cells THP1 cells with 4 μg of plasmid, or 2 × 10^5^ Huh7 and p125HEK cells using 2.5 μg of plasmid according to manufacturer’s instructions. After 24 h, mRuby-positive cells were single-cell-sorted into 96-well plates containing fresh medium for Huh7 and p125HEK cells; or 50% fresh medium and 50% filtered conditioned medium from THP1 parental cells for THP1 cells. Surviving clones were expanded and screened by immunoblotting for ablation of target protein, and Sanger sequenced to ascertain mutation at the gene locus. IRF3 KO THP1 cells have been previously described^47^. RIG-I KO MEFs were a gift from S. Akira. LIM1215 cells stably overexpressing SFSR3–GFP or GFP were described previously^72^. EMCV (gift from C. Reis e Sousa) was produced in BHK-21 cells, and harvested from supernatant by centrifugation at 10,000*g* and 0.2-μm filtration before single-aliquot freezing at −80 °C. Viral content was quantified by plaque assay on BHK-21 cells. SARS-CoV-2 Victoria/02/2020 (passage 5) was produced in Vero E6 and titrated by plaque assay as described previously^9^. For cytoplasmic RNA extraction, the BetaCoV/France/IDF0372/2020 isolate was supplied by S. van der Werf and the National Reference Center for Respiratory Viruses hosted by Institut Pasteur (Paris, France). The patient sample from which strain BetaCoV/France/IDF0372/2020 was isolated was provided by X. Lescure and P. Yazdanpanah from the Bichat Hospital, Paris, France. Moreover, the strain BetaCoV/France/IDF0372/2020 was supplied through the European Virus Archives goes Global (Evag) platform, a project that has received funding from the European Union’s Horizon 2020 research and innovation program under grant agreement no. 653316. Experiments using this strain were performed at the CEMIPAI BSL3 facility (UAR 3725 CNRS Montpellier University). The IAV strain HKx31 (H3N2) was grown in 10-day embryonated chicken eggs (kindly provided by P. Reading, University of Melbourne) and titrated on MDCK cells.

### Antibodies and reagents

All chemicals and reagents used were from Merck unless otherwise indicated. MDA5 antibodies were generated by C. Song and B. Jin. After screening, clone 16 (also referred to as Antibody A) and clone 22 (also referred to as Antibody B) were selected for native and denatured IP, respectively, and clone 17 was chosen for immunoblotting. Hybridoma cells expressing clone 16 and clone 17 were grown in bioreactors (CELLline) as per manufacturer’s instructions, and stored at −80 °C. For clone 22, the hybridoma cells were non-recoverable, therefore the antibody was de novo sequenced by protein mass spectrometry and synthesized using recombinant protein expression in mammalian cells (Rapid Novor and Absolute Antibody). In some radiolabeling experiments, as indicated in the figure legends, pooled ascites fluids from a selection of hybridoma clones including Antibody B were used for the denatured MDA5 IP step. The J2 antibody against dsRNA was from Scicons, the SRSF3 antibody was from Sigma (WH0006428M8), the MAVS (PA5-17256) and GFP (A21311) antibodies were from Thermo Fisher Scientific, the SARS-CoV-2 Spike antibody (GTX632604) was from GeneTex and the MxA antibody (clone M143, MABF938) was from Merck. The ISG15 antibody was a gift from K. P. Knobeloch. HRP-coupled secondary antibodies were sheep-anti-mouse and donkey-anti-rabbit (both GE Healthcare, 1:3,000 dilution). The anti-MDA5 antibodies protein sequences are provided in Supplementary Table 3.

### Confocal microscopy

THP1 cells were grown on sterile poly-L-lysine (0.01%, Merck)-treated glass coverslips (5 × 10^5^ cells per well on 24-well plates) with phorbol 12-myristate 13-acetate (PMA; 10 ng ml^−1^; Invivogen) for 24 h. Cells were infected with EMCV at MOI = 10 for 16 h, washed with PBS, and fixed for 15 min with 4% formaldehyde in cytoskeleton-stabilizing buffer (CSB; 5 mM KCl, 137 mM NaCl, 4 mM NaHCO_3_, 0.4 mM KH_2_PO_4_, 1.1 mM Na_2_HPO_4_, 2 mM MgCl_2_, 5 mM PIPES, 2 mM EGTA and 5.5 mM glucose in water). Cells were washed with PBS, permeabilized with 0.1% Triton-X 100 in CSB for 20 min, and incubated in 0.1 M glycine in CSB for 10 min. Cells were washed four times between all subsequent steps with PBS for 5 min each wash. Cells were incubated in blocking buffer (1% BSA and 10% normal goat serum (Abcam) in PBS) for 1 h, then for 2 h with anti-dsRNA antibody (J2, 1:200 dilution in blocking buffer) and 1 h with goat anti-mouse AlexaFluor 488 (1:500 dilution in blocking buffer; Life Technologies, A11029) and AlexaFluor 633 Phalloidin (1:40 dilution in blocking buffer; Thermo Fisher Scientific A22284). Slides were mounted using ProLong Gold antifade mountant with 4,6-diamidino-2-phenylindole (DAPI; Thermo Fisher Scientific, P36931), and imaged using a Zeiss 780 inverted confocal microscope.

### Immunoblotting

Cells were lysed with lysis buffer (10 mM Tris, 50 mM NaCl, 30 mM sodium pyrophosphate, 50 mM NaF, 5 uM ZnCl_2_, 0.5% IGEPAL and complete protease inhibitor cocktail (Roche)) and protein was quantified by BCA assay (Pierce). For LIM1215 analysis of cytoplasmic and nuclear protein, cells were lysed in RIPA buffer (50 mM Tris-HCl, pH 8, 150 mM NaCl, 1% IGEPAL, 0.5% sodium deoxycholate and 0.1% SDS). Samples were denatured using NuPAGE LDS sample loading buffer (Life Technologies) and 10% 2-mercaptoethanol, and heating at 95 °C for 5 min. Samples were resolved by electrophoresis on 4–12% Bis-Tris gels with MOPS Running Buffer (Life Technologies NuPAGE system) and transferred to nitrocellulose membrane by electrophoresis at 100 V for 2 h in cold transfer buffer (Life Technologies) with 10% methanol. Membranes were blocked with 0.05% IGEPAL in Tris-buffered saline (TBS-N; 50 mM NaCl and 50 mM Tris-HCl, pH 7.6) containing 5% non-fat milk (5% milk TBS-N) for 1 h, and probed with primary and HRP-conjugated secondary antibodies diluted in 5% milk TBS-N for 1 h at room temperature or overnight at 4 °C, with rotation. Membranes were washed four times in TBS-N for 5 min each wash after each antibody incubation. Proteins were visualized on iBright (Thermo Fisher Scientific) after exposure to Western LightningPlus-ECL chemiluminescent reagent (PerkinElmer).

### Flow cytometry

THP1 cells were grown in 12-well plates (1 × 10^6^ cells per well) and stimulated for 24 h with PMA (10 ng ml^−1^). Cells were infected with EMCV using MOIs and incubation times specified in figure legends, before addition of cold PBS containing 1% FCS (FACS Buffer). After 10 min incubation at 4 °C, cells were lifted by pipetting, and washed with FACS buffer. For all subsequent steps, reagents were diluted in FACS buffer unless stated otherwise, and washed twice with FACS buffer between steps. Cells were stained with Live/Dead Fixable Aqua Cell Stain (1:200 dilution in PBS, Life Technologies, L34957) combined with FcR block (1:200 dilution in PBS, eBioscience), fixed in 4% formaldehyde (10 min), and permeabilized in 0.1% Triton-X (20 min). Cells were stained with J2 dsRNA antibody (1:200, dilution 30 min) and goat anti-mouse AlexaFluor 488 (1:500 dilution, 30 min; Life Technologies, A11029), and resuspended in CellFix (1:10 dilution in water; BD, 340181). Cells were analyzed by flow cytometry on an Attune NxT Flow Cytometer (Thermo Fisher Scientific) and FlowJo software (BD).

Calu-3 cells were grown in six-well plates (1 × 10^6^ cells per well) and infected with SARS-CoV-2 at an MOI of 0.1 for 48 h. Cells were washed with PBS, lifted using Trypsin for 15 min at 37 °C and fixed using formalin for 20 min at room temperature (RT). Cells were stained in separate wells with anti-SARS-CoV-2 Spike (1:200 dilution) and anti-MxA (1:300 dilution) antibodies for 30 min at 4 °C, then with goat anti-mouse IgG AlexaFluor-647 (1:500 dilution; Life Technologies) and goat anti-mouse IgG2a AlexaFluor-647 (1:500 dilution, BioLegend) for 30 min at 4 °C using Saponin buffer (PBS, 1% BSA and 0.1% saponin) for all antibody incubations, and washed twice in the same buffer between steps. Cells were resuspended in FACS buffer and analyzed by flow cytometry using a NovoCyte flow cytometer (ACEA Biosciences) and FlowJo software (BD).

### MDA5 iCLIP

Conditions were maintained RNase-free throughout the experiment, conducted as described in Huppertz et al.^48^, with some modifications. IRF3-KO or MDA5-KO THP1 cells (2 × 10^7^ cells in 20-cm plates, 10–20 plates per condition) were incubated with PMA overnight, then infected with EMCV (MOI = 5) for 22 h, or left uninfected (IRF3-KO only). Cells were washed twice with PBS and UV-crosslinked using 150 mJ cm^−2^ in a Spectrolinker XL-1500 (Spectronics Corp). Cells were scraped, centrifuged at 400*g* for 5 min, and PBS supernatant was discarded. Cell pellets were flash frozen and stored at −80 °C until ready for use. Cell pellets were lysed in lysis buffer (10 mM Tris, 50 mM NaCl, 30 mM sodium pyrophosphate, 50 mM NaF, 5 μM ZnCl_2_, 1% IGEPAL and complete protease inhibitor cocktail (Roche)) using equivalent of 400 μl of buffer per plate of cells. Samples were incubated on ice for 10 min and clarified by centrifugation at 10,000*g* for 10 min. Protein concentration was determined by BCA assay. Then, 5,000 μg of clarified cell lysate was used per condition. RNase A (Affymetrix, 70194Y; 20 U/μl) was added at 1:10,000 dilution to samples and incubated for 5 min, followed by cooling and addition of 1:80 dilution of RNAsin Plus RNase inhibitor (Promega; N2611). Anti-MDA5 monoclonal antibody 16 was covalently coupled to Dynabeads (Invitrogen, 14311D) according to manufacturer’s instructions, using 9 mg Dynabeads and 180 ug of monoclonal antibody 16 per sample. Monoclonal antibody 16 antibody-coupled Dynabeads were washed with high salt buffer (50 mM Tris, pH 7.4, 1 M NaCl, 1% IGEPAL, 0.1% SDS and 0.5% deoxycholate) and lysis buffer, and incubated with protein samples for 2 h at 4 °C with rotation. Then, 1% of sample was collected and stored at 4 °C, for use as size-matched input (SMI). Samples were washed twice for 5 min with high-salt buffer, and once with PNK buffer (20 mM Tris pH 7.4, 10 mM MgCl_2_ and 0.2% Tween-20). Supernatant was removed, 300 μl of urea cracking buffer (50 mM Tris pH 7.4, 6 M urea, 1% SDS and 25% PBS) was added, and samples incubated with shaking for 3 min at 65 °C, before neutralization with 3 ml of T-20 IP buffer (50 mM Tris, pH 7.4, 150 mM NaCl, 0.5% Tween-20 and 0.1 mM EDTA) and addition of RNase and protease inhibitors. A second round of immunoprecipitation was done using anti-MDA5 monoclonal antibody 22 antibody preincubated with Protein G Dynabeads (Life Technologies; 900 μl beads and 90 μg of antibody) according to manufacturer’s instructions. Samples were incubated with beads for 2 h at 4 °C with rotation, and washed twice for 5 min with high-salt buffer, and twice with PNK buffer. RNA in samples was 3’ end-dephosphorylated using T4 PNK (New England Biolabs, M0201L) for 20 min, with addition of RNase inhibitor and Turbo DNase I (Life Technologies AM2238, 2 U μl^−1^). Samples were washed twice with RNA ligase buffer (5 mM Tris-HCl, pH 7.5 and 1 mM MgCl_2_), and iCLIP 3’ linker (Trilink, O-30050-03) was ligated to the 3′ end of RNA molecules using T4 RNA Ligase 1 High Concentration (New England Biolabs, M0437M) with the addition of 2.6% dimethylsulfoxide, 40% PEG-8000 and 2% RNase inhibitor, for 2 h at 25 °C with shaking. Samples were washed twice with high salt buffer, changing tubes, and twice with PNK buffer. Then, 10% of the sample was set aside for radiolabeling using T4 PNK and γ-32P-ATP (Hartmann Analytic; FP-301; 3,000 Ci mmol^−1^; 10 mCi ml^−1^) according to manufacturer’s instructions. IP and SMI samples were denatured using sample loading buffer and dithiothreitol (DTT) and incubated for 10 min at 70 °C. Samples were resolved on 4–12% Bis-Tris gels (Life Technologies; NP0322BOX) using MOPS SDS running buffer (Life technologies; NP0001) for 2 h at 150 V. Samples were transferred to nitrocellulose for 4 h at 100 V using cold Transfer Buffer (Life technologies; NP0006) with 10% methanol. Membrane was exposed to film for signal visualization, and the radiograph was used as a guide to excise samples from membrane.

Samples of MW > 135 kDa were excised, and membrane was cut into small pieces and placed in a microcentrifuge tube. RNA was released from membrane by protein digestion using Proteinase K (Roche, 03115844001) for 1 h at 50 °C, according to manufacturer’s instructions. RNA was purified by phenol:chloroform:iaa (P3803-100ML) with phaselock gel tubes (5 Prime), and ethanol precipitation. For SMI samples, protocol was done as described^52^. IP and SMI samples were reverse transcribed with primers shown in Supplementary Table 1, using TGIRT enzyme (InGex; CM0101-50) according to manufacturer’s instructions. Samples were ethanol precipitated, resuspended in TBE urea sample buffer (Life Technologies; LC8676) and resolved on 6% TBE urea gels (Life Technologies; EC6865BOX) for 40 min at 180 V. cDNA sized between 80 and 200 nt was excised, fragmented, and incubated in 400 μl diffusion buffer (0.5 M ammonium acetate, 10 mM magnesium acetate, 1 mM EDTA and 0.1% SDS) for 30 min at 50 °C, shaking. Sample was clarified by centrifugation in SpinX columns (Costar) containing two 10-mm glass pre-filters (Whatman, 1823-010), and purified by phenol:chloroform:iaa and ethanol precipitation. cDNA samples were circularized using CircLigase II (Cambio, CL9021K) and BamHI digested (New England Biolabs) as described^48^. Samples were PCR amplified using the P3 and P5 Solexa primers and Accuprime PCR Master Mix (Thermo Fisher Scientific), starting at 20 cycles and increasing in 1–2 cycle steps, until a signal was detectable by Tapestation D1000 High sensitivity kit. Libraries were concentrated using the MiniElute PCR Purification kit (QIAGEN) and resolved on a 6% TBE gel (Life Technologies, EC6265BOX) for 30 min at 180 V. Gel was stained with SYBRGold (Thermo Fisher Scientific), and fragments between 100 and 250 bp were excised, extracted from gel as above, and purified using QIAQuick gel extraction kit (QIAGEN). Samples were quantified by Qubit High Sensitivity DNA kit (Thermo Fisher Scientific, Q32851) and KAPA Library quantification kit (Illumina, 07960093001). Samples were equalized to 4 nM, and combined at a ratio of 10:1 IP samples to SMI samples, in order to increase sequencing from IP samples. Libraries were sequenced on Illumina NextSeq500 using 75 cycle High Output kit v2 (TG-160-2105). Each biological repeat was sequenced on a separate chip.

For MDA5 iCLIP from SARS-CoV-2-infected and uninfected Calu-3 cells, the protocol above was followed, with some modifications as follows^104,105^. A total of 2.5 × 10^7^ cells in 20-cm plates (two plates per condition) were left uninfected or infected with SARS-CoV-2 at an MOI of 0.1 for 48 h. After dual IP and adaptor ligation, samples were directly treated with proteinase K, without SDS–PAGE size selection. Samples were reverse transcribed with primers shown in Supplementary Table 2 (ref. ^105^), which contained longer UMI and barcode sequences, and carbon spacers to stop the progression of PCR during amplification, removing the need for linearization after circularization of libraries. A pre-amplification PCR step of six cycles before library size selection was performed to increase retention of unique sequences^104^. Libraries were then size-selected using ProNex Chemistry (Promega; NG2001), PCR amplified, and size-selected again as described^104^. Libraries were sequenced on Illumina NextSeq 2000 using 100 cycle P3 Reagents (20040559). All samples were sequenced on a single chip.

### iCLIP data processing and analysis

A diagram of iCLIP data processing is shown in Extended Data Fig. 3a, and largely followed recommendations outlined elsewhere^106^. Fastx-trimmer (http://hannonlab.cshl.edu/fastx_toolkit/) was used to remove low-quality barcode regions as described elsewhere^106^. Flexbar (https://github.com/seqan/flexbar) was used to simultaneously 3’ end trim, demultiplex, and extract UMIs. FastQC was used to determine quality of sequencing reads pre- and post-processing (https://www.bioinformatics.babraham.ac.uk/projects/fastqc/). A custom genome and gtf file containing both the human genome (hg38 assembly) and the viral genome (EMCV DQ288856.1; SARS-CoV-2 NC_045512) were prepared. Samples were mapped against the combined human-viral genome using STAR^107^ with the following parameters: STAR–runMode alignReads–runThreadN 8–limitBAMsortRAM 10000000000–outSAMattributes All–outStd BAM_SortedByCoordinate–outSAMtype BAM SortedByCoordinate–outFilterType BySJout–outReadsUnmapped Fastx–outSAMattrRGline ID:foo–alignEndsType Extend5pOfRead1–outFilterMismatchNoverReadLmax 0.04–outFilterMismatchNmax 999–outFilterMultimapNmax 1–sjdbOverhang maxReadLength-1–outSJfilterReads Unique. Samples were indexed using SAMtools and deduplicated based on UMI using umi_tools dedup^108^. MultiQC was used to quantify rates of mapping^109^. For the EMCV-infected iCLIP experiment, the STAR parameter–outFilterMultimapNmax 10 was used. This permited reads multimapping in up to ten locations, with retention of only one read in the output, to enable mapping in repetitive sites. For the SARS-CoV-2 infection iCLIP experiment, reads had lower multimapping rates and enabled retention of only uniquely mapped reads. A custom computational pipeline was written using ruffus^110^ to automate the sample processing described above. This is available for download at https://github.com/natsampaio/iCLIP_pipeline.

To determine crosslinking sites, the PureCLIP peak-calling algorithm was applied^53^. Chromosomes 1–6 were used for the algorithm parameter learning, and CL motifs and SMI controls were incorporated. For motif analysis, FASTA sequences containing 50 bp either side of crosslinking site were obtained using bedtools^111^ commands slop and getfasta. Sequences present in the negative control samples (MDA5-KO cells or uninfected cells) and present in rRNA and tRNA (database from SILVA; https://www.arb-silva.de/) were subtracted from the sequences from EMCV-infected cells using bedtools slop. The resulting sequences were analyzed using the MEME suite (v.5.0.1) meme-chip algorithm, with -norc and -centrimo-local parameters included^55^. The same sequences were also analyzed using HOMER^54^ function findMotifsGenome.pl, with -rna. Basic analysis compared test sequences to automatically generated random genetic background. Test sequences were also compared to either MDA5-KO or uninfected sequences using the -bg option. Genomic context of sequences was determined using the bedtools command intersect to quantify number of sequences overlapping with annotated introns, exons, 5’ UTR or 3’ UTR in the genome. Sequences that contained crosslink sites were analyzed using RepeatMasker^112^ to quantify repetitive regions and bedtools intersect using a database of annotated repetitive regions in the genome. To investigate overlapping binding sites in opposite strands, a random control dataset bed file was generated using the bedtools command random, inputting the combined human and SARS-CoV-2 genome used for iCLIP read mapping.

PCA plots of iCLIP data were generated in R (v.4.5.0) using functions from the DiffBind package^113^ (v.3.20.0). A DBA object was created from the deduplicated IP and SMI BAM files, corresponding BAM index files and crosslinking sites for each sample using the dba function. Background normalization of IP reads was performed with the dba.normalize function using both SMI control reads and TMM normalization (normalize = DBA_NORM_TMM, spikein = TRUE), and PCA plots generated from normalized data using dba.plotPCA.

### In vitro RNA transcription and purification

For production of in vitro transcribed RNA (IVT-RNA), genomic DNA was extracted from THP1 cells using DNeasy Blood & Tissue kit (QIAGEN, 69504) and used as PCR template. Herculase II PCR kit (Agilent, 600677) was used to PCR amplify desired regions with addition of T7 polymerase promoter sequence. MegaScript T7 kit (Invitrogen, AM1334) was used for production of IVT-RNA according to manufacturer’s instructions. RNA was purified using RNeasy Mini kit (QIAGEN, 74104), and quantified by Nanodrop (Thermo Fisher Scientific) and stored at −80 °C in single-use aliquots. RNA assessed for size and purity using the Tapestation (Agilent). To produce dsRNA, forward and reverse complementary IVT-RNA strands were heated to 90 °C and cooled at 1 °C every 30 s until 25 °C, then used immediately for cell transfection.

For generation of motif-deleted or MIR deleted IVT-RNA sequences, overlap extension PCR was designed using primers that anneal upstream of the specific feature, with overhang sequences that anneal downstream of the feature, and vice versa. The two-part PCR products were then annealed and used to generate a single DNA length that skipped the specific feature. These were used as the template for IVT-RNA generation as described above. Neo^1–99^ IVT-RNA represents single-stranded IVT-RNA generated from base 1–99 of the Neomycin gene, as described^29^.

### Cell transfection of IVT-RNA

Cells were grown in 12-well plates (1.75 × 10^6^ cells per well) for 24 h, and transfected with 175 ng of IVT-RNA, 87.5 ng of high MW Poly(I:C) (Invivogen, tlrl-pic) or 35 ng of Neo^1–98^ (ref. ^114^) IVT-RNA using 0.7 μl of Lipofectamine 2000 (Thermo Fisher Scientific, 11668027) according to the manufacturer’s instructions. Huh7 cells were treated with 30 U ml^−1^ IFN-A/D (R&D Systems) for 24 h before transfection. RNA was extracted after 24 h using RNeasy Plus Mini kit (QIAGEN, 74136) according to manufacturer’s instructions, quantified by Nanodrop, and stored at −80 °C.

### RT–qPCR

RNA (1 μg) was converted into cDNA using SuperScript III Reverse Transcriptase (Thermo Fisher Scientific) and Oligo-dT primers (Invitrogen) according to manufacturer’s instructions. cDNA was diluted to 100 ng μl^−1^ and quantitative PCR was performed using TaqMan Real-Time PCR Assays for designated genes and TaqMan Fast Advanced Master Mix (Thermo Fisher Scientific) according to manufacturer’s instructions. Alternatively, quantitative PCR was performed using SYBR Green PCR Master Mix (Thermo Fisher Scientific) with gene specific probes detailed in Supplementary Table 2. Assays were performed on QuantStudio 6 Flex Real-Time PCR machines (Thermo Fisher Scientific). Two-way ANOVA was performed with Sidak’s multiple comparisons test using GraphPad Prism software.

### *IFNB1* promoter luciferase assay

p125-HEK293 RIG-I-KO^47^ cells and p125-HEK293 RIG-I-KO/MDA5-KO cells (Extended Data Fig. 6a,b) stably expressing a Renilla luciferase downstream of an *IFNB1* promoter were plated in 96-well plates (2.75 ×10^4^ cells per well) together with 30 U ml^−1^ IFN-A/D. On the following day, cells were transfected with 20 ng of either IVT-RNA, EMCV CIP RNA, high MW Poly(I:C) (Invivogen, tlrl-pic) or Neo^1–99^ IVT-RNA using 0.4 μl of Lipofectamine 2000 (Thermo Fisher Scientific, 11668027) according to manufacturer’s instructions. After 20–24 h, half the culture medium was removed and replaced with an equal volume of ONE-Glo luciferase assay reagent (Promega, E6120). After 3 min, 85 μl of sample was transferred to a white 96-well plate and luminescence was measured on a GloMax Luminometer (Promega).

### Cytoplasmic RNA extraction and RNA sequencing

To investigate aberrant RNA expression in cells upon virus infection, IRF3-KO THP1 cells were incubated with 10 ng ml^−1^ PMA overnight, then left untreated, infected with EMCV (MOI = 2) for 20 h, or treated with human IFNβ (Rebif) for 6 hr. Calu-3 cells were seeded in six-well plates (1 × 10^6^ cells per well) overnight, then left untreated, infected with SARS-CoV-2 (MOI = 0.1) for 48 h or treated with human IFNβ (PBL assay science) for 6 h. Cytoplasmic RNA was extracted using Cytoplasmic & Nuclear RNA Purification kit (Norgen) according to manufacturer’s instructions. Total RNA libraries were prepared, starting with 500 ng of total RNA, according to the Illumina Tru-Seq Stranded Total RNA-Seq protocol (protocol 1000000040499) with RiboZero depletion, using unique dual indexes. Then, 150-bp-end sequencing was performed on a NextSeq 2000 P3 chip, loading pooled libraries at a concentration of 1,000 pM (protocol 1000000109376). Approximately 100 million reads were obtained per sample. Base calling was performed using Dragen BCLConvert (v.3.7.4).

### Analysis of aberrant RNA transcription

Cytoplasmic RNA sequencing data were processed as described previously^58^ with minor modifications. In brief, reads were trimmed using fastp^115^ and subjected to an additional in silico rRNA depletion step before alignment with STAR^107^ against the T2T-CHM13v2 (ref. ^116^) assembly (GENCODE (v.48)^117^ lifted via Liftoff^118^). For the analysis of aberrant host transcription, downstream, divergent and read-through regions of expressed coding genes were standardized to a fixed length of 10 kb; regions shorter than this cutoff or associated with lowly expressed genes (reference exon FPKM < 1) were excluded. Differential aberrant expression was quantified using DEXSeq^119^ by comparing the ratio of reads in aberrant regions to reference exons across conditions (a two-sided test with Benjamini–Hochberg correction); changes with a log_2_(fold change) ≥ 0.585 (*P*adj ≤ 0.01) were considered significant.

### Intron retention and upregulation analysis

Cytoplasmic RNA sequencing read alignment was performed in R (v.4.1.2)^120^ using the Rsubread package (v.2.6.4)^121^. A genome index was built using the custom combined human and EMCV genome using the buildindex function and alignment was performed using sunjunc, with default settings. Exon- and intron-level differential gene expression analyses followed the methods of the index package (v.1.0)^121^. A custom GTF file consisting of human and EMCV data was used for mapping. This GTF file was processed using the Annotations scripts from the Intron-reads repository (https://github.com/charitylaw/Intron-reads) to define exon and gene body regions. Counts were obtained using featureCounts from Rsubread (v.2.8.1), with isPairedEnd and useMetaFeatures set to TRUE, allowMultiOverlap set to FALSE and strandSpecific 2. Intron counts were obtained by subtracting exon counts from gene body counts.

Two DGEList objects were created using the edgeR package (v.3.36.0)^122^ using the exon and intron counts respectively, as well as sample annotation and gene annotation, with additional annotation obtained from the biomaRt package (v.2.50.1)^123,124^. Sample 1C_IFNb, a technical replicate of sample 3C_IFNb, was excluded from further analysis. A design matrix was created incorporating the treatment group, so that the treatments could be compared, as well as the experimental replicate, to control for the experimental batch effect. Total library sizes were obtained by summing the exon and intron counts. Lowly expressed genes, on either an exonic or intronic level, were removed according to the filterByExpr function, incorporating the design matrix. As such, single-exon genes were also excluded from further analysis. Normalization factors were calculated using the calcNormFactors function with the TMM method^125^.

Counts were processed using the voom method^126^ and a linear model was fit using the edgeR voomLmFit function, including the design matrix. Comparisons between EMCV-treated, IFNβ-treated and untreated groups were made using the contrasts.fit function from the limma package (v.3.50.0)^127^. Empirical Bayes moderated *t*-tests were performed against a 1.2-fold change threshold and *P* values were obtained using the treat^128^ function for differential expression analysis or without a fold change threshold using the eBayes function^129^ for subsequent comparison to iCLIP data.

Results of the intron-level analysis were visualized as mean-difference plots, showing the log_2_ average expression and log_2_ fold changes, highlighting genes significantly differentially expressed at the intron level, with Benjamini–Hochberg adjusted *P* values < 0.05. Differential expression results were visualized by plotting exon- versus intron-level log_2_ fold changes on a per gene basis, quantifying the number of genes belonging to different differential expression categories, following the plot_index function from the index package.

IRFinder (v.1.30)^60^ was used to examine differential intron retention. Intron retention was quantified in BAM mode using the BAM files obtained from the Rsubread subjunc alignment, utilizing a genome reference built using IRFinder BuildRefProcess on the combined human and EMCV genome and GTF files. Differential intron retention was performed in R using the DESeq2 package (v.1.34.0)^130^, following the DESeq2Constructor.R script provided by IRFinder. The resulting DESeqDataSet object was filtered to only include introns that had been designated as clean by IRFinder (excluding known-exon regions). Furthermore, introns were only included if they either had no warnings or were only flagged as NonUniformIntronCover in at least three samples (thereby excluding introns that had been categorized as LowCover, LowSplicing or MinorIsoform in most samples). The design matrix was defined as ~Group + Group:IRFinder + Replicate and differential expression performed using the DESeq function. The Group:IRFinder interaction term was used for finding differences in intron retention, by comparing GroupEMCV.IRFinderIR and GroupIFNb.IRFinderIR to GroupUnif.IRFinderIR with the DESeq2 results function.

Results of the intron-retention analysis were visualized as mean-difference plots, showing log_2_ (base mean expression + 1) and log_2_ fold changes, highlighting significantly differentially retained introns with Benjamini–Hochberg adjusted *P* values < 0.05. To compare the cytoplasmic RNA-seq to the iCLIP results, bedtools intersect was used in stranded mode to find genes from the index analysis and introns from the IRFinder analysis that overlapped with intronic EMCV iCLIP peaks. Results were visualized using the barcodeplot function from the limma package, either using the index EMCV intron-level empirical Bayes moderated *t*-statistics or IRFinder DESeq2 EMCV intron retention test statistics, showing all genes or introns that overlapped with iCLIP sites with scores higher than the 95% percentile. The iCLIP scores were used for the barcodeplot gene.weights argument; if more than one iCLIP site were overlapping a gene or intron, the higher score was used. In addition, *P* values were obtained using the roast^131^ function from the edgeR package, using DGEList objects created from the counts from the index and IRFinder analyses. Dispersions were estimated using the estimateDisp function^132^ with robust set to TRUE^129^, then the roast tests were performed using the iCLIP score weights with 19,999 rotations and up-direction *P* values were reported. Integrative Genomics Viewer (IGV v.2.8.9)^133^ was used to visualize data.

### Feature analysis of MDA5-bound introns

iCLIP peak coordinates for MDA5 in Calu-3 and THP1 cells (infected or uninfected) were extracted from BED files and converted to GRanges. Intron coordinates were derived from Ensembl (GRCh38) exon annotations obtained via biomaRt: exons were grouped by gene, ordered, reduced and introns defined as the gaps between adjacent exons. The resulting intron set was intersected with iCLIP peaks; only peaks mapping within annotated introns were retained.

To define the background repertoire of introns, splicing events (ASEs) were identified using rMATS Turbo (v.4.3.0)^134^, with STAR-aligned BAM files and GENCODE v.M34 as input. Sequence feature analysis was performed with Matt^135^, comparing introns containing MDA5 binding to the rMATS-defined background (NO group).

### Incucyte cell viability assay

LIM1215–GFP and LIM1215–SRSF3–GFP cells were plated at 1 × 10^5^ cells per well in 96-well plates, incubated for 24 h, and medium exchanged to fresh medium containing Incucyte Cytotox NIR Dye (Sartorius) at a 1:2,000 dilution. Cells were infected with EMCV or IAV at the indicated MOIs and monitored on the Incucyte SX5 live-cell imaging system (Sartorius) for 72 h. Cell death was assessed using the Incucyte software.

### Reporting summary

Further information on research design is available in the Nature Portfolio Reporting Summary linked to this article.

## Online content

Any methods, additional references, Nature Portfolio reporting summaries, source data, extended data, supplementary information, acknowledgements, peer review information; details of author contributions and competing interests; and statements of data and code availability are available at 10.1038/s41590-026-02614-3.

## Supplementary information

Supplementary Information Supplementary Tables 1–3.

Reporting Summary

Peer Review File

## Source data

Source Data Fig. 1 Unprocessed blots.

Source Data Fig. 2 Statistical source data.

Source Data Fig. 3 Statistical source data.

Source Data Fig. 4 Statistical source data.

Source Data Fig. 5 Statistical source data.

Source Data Fig. 6 Statistical source data and unprocessed blots.

Source Data Extended Data Fig. 1 Statistical source data and unprocessed blots.

Source Data Extended Data Fig. 2 Unprocessed blots.

Source Data Extended Data Fig. 4 Statistical source data.

Source Data Extended Data Fig. 5 Unprocessed blots.

Source Data Extended Data Fig. 6 Statistical source data and unprocessed blots.

Source Data Extended Data Fig. 7 Statistical source data.

Source Data Extended Data Fig. 8 Statistical source data.

## Data Availability

Cytoplasmic RNA sequencing data were uploaded to the NCBI Gene Expression Omnibus under the SuperSeries accession GSE214664. Source data are provided with this paper.
