# Supplementary material for: Loss of cellular RNA homeostasis contributes to MDA5 activation during virus infection
Source: Nat Immunol. 2026 Aug 11;27(9):1815–28. doi: 10.1038/s41590-026-02614-3 (PMC13506345; doi:10.1038/s41590-026-02614-3)
Supplement: Supplementary file 1 [file 41590_2026_2614_MOESM1_ESM.pdf]

nature immunology

Supplementary information https://doi.org/10.1038/s41590-026-02614-3

Loss of cellular RNA homeostasis contributes to MDA5 activation during virus infection

In the format provided by the authors and unedited

Supplementary tables

Table S1 – *in vitro* transcribed RNA regions

IVT-RNA	Genome location	Genomic feature	Repeat type	poly(A/T)	AACC
EMCV-A	chr1:178479528-178480529	exon	-	yes	no
EMCV-C	chr4:39018551-39019552	repeat	Alu	yes	no
EMCV-D	chr4:87455177-87456178	intron	Alu	yes	no
EMCV-F	chr6:105763342-105764343	intergenic	-	no	yes
EMCV-G	chr7:12265267-12266268	intergenic	-	yes	no
EMCV-H	chr8:120427649-120428650	repeat	L1	no	yes
EMCV-I	chr8:127351876-127352877	intron	L2	yes	no
EMCV-J	chr9:101873671-101874672	repeat	L1	yes	no
EMCV-L	chr10:45753353-45754354	intron	-	yes	no
EMCV-M	chr14:52620126-52621127	repeat	L1	no	no
EMCV-N	chr14:62409858-62410859	repeat	MIR	yes	no
EMCV-O	chr15:42176691-42177692	repeat	Alu	no	yes
EMCV-P	chr18:43170031-43171032	repeat	ERV1	yes	no
mock-3	chr1:148388122-148389123	intergenic	Alu	no	no
mock-8	chr2:30714060-30714661	intergenic	MIR	yes	yes
mock-17	chr4:81593996-81594597	intergenic	L2 (partial)	yes	no
mock-18	chr5:91121116-91122117	intron	-	no	no
mock-23	chr5:122007732-122008733	intron	L1	no	yes
mock-24	chr6:33112397-33113398	intron	-	no	yes
mock-26	chr11:113786882-113787883	intergenic	-	no	yes
mock-33	chr12:123487347-123488348	intron	Alu	no	yes
mock-35	chr13:29507172-29508173	intergenic	-	no	yes
mock-37	chr14:96697011-96698012	intergenic	MIR	no	yes
mock-42	chr15:86322525-86323526	intron	-	no	yes
mock-44	chr17:52675234-52676235	intergenic	hAT-Charlie	no	yes
mock-46	chr17:65598620-65599621	intergenic	MIR	no	yes
mock-49	chr19:22694358-22695359	intergenic	ERV1	no	no

Table S2 – DNA primer/probe sequences

Name	Method	Sequence
Rt1clip	iCLIP reverse transcription	/5Phos/NN AACC NNN AGATCGGAAGAGCGTCGTGgatcCTGAACCGC
Rt2clip	iCLIP reverse transcription	/5Phos/NN ACAA NNN AGATCGGAAGAGCGTCGTGgatcCTGAACCGC
Rt3clip	iCLIP reverse transcription	/5Phos/NN ATTG NNN AGATCGGAAGAGCGTCGTGgatcCTGAACCGC
Rt4clip	iCLIP reverse transcription	/5Phos/NN AGGT NNN AGATCGGAAGAGCGTCGTGgatcCTGAACCGC
Rt8clip	iCLIP reverse transcription	/5Phos/NN CATT NNN AGATCGGAAGAGCGTCGTGgatcCTGAACCGC
Rt9clip	iCLIP reverse transcription	/5Phos/NN GCCA NNN AGATCGGAAGAGCGTCGTGgatcCTGAACCGC
iiCLIP_ddRT_12	iCLIP reverse transcription	/5Phos/ WWW GTGGA NNNN AGATCGGAAGAGCGTCGTGAT /iSp18/ GGATCC /iSp18/ TACTGAACCGC
iiCLIP_ddRT_13	iCLIP reverse transcription	/5Phos/ WWW TCCGG NNNN AGATCGGAAGAGCGTCGTGAT /iSp18/ GGATCC /iSp18/ TACTGAACCGC
iiCLIP_ddRT_14	iCLIP reverse transcription	/5Phos/ WWW TGCCT NNNN AGATCGGAAGAGCGTCGTGAT /iSp18/ GGATCC /iSp18/ TACTGAACCGC
iiCLIP_ddRT_15	iCLIP reverse transcription	/5Phos/ WWW TATTC NNNN AGATCGGAAGAGCGTCGTGAT /iSp18/ GGATCC /iSp18/ TACTGAACCGC
iiCLIP_ddRT_16	iCLIP reverse transcription	/5Phos/ WWW TTAAA NNNN AGATCGGAAGAGCGTCGTGAT /iSp18/ GGATCC /iSp18/ TACTGAACCGC
iiCLIP_ddRT_17	iCLIP reverse transcription	/5Phos/ WWW AAATG NNNN AGATCGGAAGAGCGTCGTGAT /iSp18/ GGATCC /iSp18/ TACTGAACCGC
iiCLIP_ddRT_18	iCLIP reverse transcription	/5Phos/ WWW AAGGT NNNN AGATCGGAAGAGCGTCGTGAT /iSp18/ GGATCC /iSp18/ TACTGAACCGC
iiCLIP_ddRT_19	iCLIP reverse transcription	/5Phos/ WWW AATAC NNNN AGATCGGAAGAGCGTCGTGAT /iSp18/ GGATCC /iSp18/ TACTGAACCGC
iiCLIP_ddRT_20	iCLIP reverse transcription	/5Phos/ WWW ACGCA NNNN AGATCGGAAGAGCGTCGTGAT /iSp18/ GGATCC /iSp18/ TACTGAACCGC
iiCLIP_ddRT_21	iCLIP reverse transcription	/5Phos/ WWW ACTTG NNNN AGATCGGAAGAGCGTCGTGAT /iSp18/ GGATCC /iSp18/ TACTGAACCGC
iiCLIP_ddRT_22	iCLIP reverse transcription	/5Phos/ WWW AGAGC NNNN AGATCGGAAGAGCGTCGTGAT /iSp18/ GGATCC /iSp18/ TACTGAACCGC
iiCLIP_ddRT_23	iCLIP reverse transcription	/5Phos/ WWW AGTCT NNNN AGATCGGAAGAGCGTCGTGAT /iSp18/ GGATCC /iSp18/ TACTGAACCGC
EMCV 5NTR_F	SYBR Green qPCR	GTCTGTAGCGACCCTTTG
EMCV 5NTR_R	SYBR Green qPCR	CCTTGTTGAATACGCTTGAG
IAV NS1_F	SYBR Green qPCR	AAGACCAATCTTGTCACCTCTGA
IAV NS1_R	SYBR Green qPCR	TCCTCGCTCACTGGGCA
hCXCL10_F	SYBR Green qPCR	TTCCTGCAAGCCAATTTTGT
hCXCL10_R	SYBR Green qPCR	TTCTTGATGGCCTTCGATTC

hDDX58_F	SYBR Green qPCR	AAAAACAACAAGGGCCCAAT
hDDX58_R	SYBR Green qPCR	TCCCAACTTTCAATGGCTTC
hGAPDH_F	SYBR Green qPCR	CCTCCTGTTCGACAGTCAGC
hGAPDH_R	SYBR Green qPCR	CCTAGCCTCCCGGGTTTCTC
hIFI44_F	SYBR Green qPCR	CAGACAGAGCAGCTACCCTCA
hIFI44_R	SYBR Green qPCR	GTGCAACCATGTCAAACGAG
hIFI44L_F	SYBR Green qPCR	ACCGTGGCTGCTCGATAAA
hIFI44L_R	SYBR Green qPCR	AGCTTGCGCAGATGATTTTC
hIFIT1_F	SYBR Green qPCR	AGCTTACACCATTGGCTGCT
hIFIT1_R	SYBR Green qPCR	CCATTTGTACTCATGGTTGCTGT
hIFNA2_F	SYBR Green qPCR	TGGGCTGTGATCTGCCTCAAAC
hIFNA2_R	SYBR Green qPCR	CAGCCTTTTGGAACTGGTTGCC
hIFNB1F	SYBR Green qPCR	GAGCTGTAGTGGAGAAGCACA
hIFNB1_R	SYBR Green qPCR	GCGACACTGTTCGTGTTGTC
hISG15_F	SYBR Green qPCR	GCGAACTCATCTTTGCCAGT
hISG15_R	SYBR Green qPCR	AGCATCTTCACCGTCAGGTC
hMX1_F	SYBR Green qPCR	GGTGGTGGTCCCCAGTAATG
hMX1_R	SYBR Green qPCR	ACCACGTCCACAACCTTGTCT
hOAS1_F	SYBR Green qPCR	CCGTGAAGTTTGAGGTCCAG
hOAS1_R	SYBR Green qPCR	GGTTTATAGCCGCCAGTCAA
hOAS2_F	SYBR Green qPCR	GAAGCCCTACGAAGAATGTCAGA
hOAS2_R	SYBR Green qPCR	TCGGAGTTGCCTCTTAAGACTGT

Table S3 – Protein sequences of MDA5 monoclonal antibodies

Antibody clone	Heavy chain	Light chain
mAb 16	QVQLQQSGPDLVRPGTSVKMSCKAAGYTFTNSWIGWL KQRPGHGLEWIGDIYPGGGDTNSNENFKGKATLTADTS SSTAYMQLSSLTSEDSAVYYCARSTVIMRGYFDVWGA GTMVTVSSAKTTPPSVYPLAPGSAAQTNSMVTLGCLV KGYFPEPVTVTWNSGSLSSGVHTFPAVLQSDLYTLSSS VTVPSSTWPSETVTCNVAHPASSTKVDKKIVPRDCGCK PCICTVPEVSSVFIFPPKPKDVLTITLTPKVTCVVVDISK DDPEVQFSWFVDDVEVHTAQTQPREEQFNSTFRSVSEL PIMHQDWLNGKEFKCRVNSAAFPAPIEKTISKTKGRPK	DIQMTQSPASLSASVGETV TITCRASGNIHNFLAWYQQ KQGKSPQLLVYNAKTLAD GVPSRFSGSGSGTQYSLKIN SLQPEDFGSYYCQHFWSTP LTFGAGTKLELKRADAAPT VSIFPPSSEQLTSGGASVVC FLNNFYPKDINVKWKIDGS ERQNGVLNSWTDQDSKDS

	APQVYTIPPPKEQMAKDKVSLTCMITDFFPEDITVEWQ WNGQPAENYKNTQPIMDTDGSYFVYSKLNVQKSNWE AGNTFTCSVLHEGLHNHHTEKSLSHSPGK	TYSMSSTLTLTKDEYERHN SYTCEATHKTSTSPIVKSFN RNEC
mAb 17	QVQLQQSGAELVRPGASVTLSCKASGYTFADYEIHWV RQTPVHGLEWIGAIGPETGNTAYNQKFKGKATLTVDKS SSTVYMDLRSLTSEDSAVYYCTRGRILDWGQGTLVTVS AAKTTPPSVYPLAPGSAAQTNSMVTLGCLVKGYFPEPV TVTWNSGSLSSGVHTFPAVLQSDLYTLSSSVTVPSSTW PSETVTCNVAHPASSTKVDKKIVPRDCGCKPCICTVPEV SSVFIFPPKPKDVLTITLTPKVTCVVVDISKDDPEVQFSW FVDDVEVHTAQTQPREEQFNSTFRSVSELPIMHQDWLN GKEFKCRVNSAAFPAPIEKTISKTKGRPKAPQVYTIPPP KEQMAKDKVSLTCMITDFFPEDITVEWQWNGQPAENY KNTQPIMDTDGSYFVYSKLNVQKSNWEAGNTFTCSVL HEGLHNHHTEKSLSHSPGK	DVVMTQTPLSLPVSLGDQA SISCRSSQSLVHSNGNTYLH WYLQKPGQSPKLLIYKVSK RFSGVPDRFSGSGSGTDFTL KISRVEAEDLGVYFCSQST HIPYTFGGGTKLEIKRADA APTVSIFPPSSEQLTSGGAS VVCFLNNFYPKDINVKWKI DGSERQNGVLNSWTDQDS KDSTYSMSSTLTLTKDEYE RHNSYTCEATHKTSTSPIVK SFNRNEC
mAb 22	QVQLQQSGPELVKPGASVKMSCKASGYTFTDYVISWV KQRTGQGLEWIGEIYPGSGNTFYNEKFKGKATLTADKS SNTAYMQLSSLTSEDSAVYFCARSGDYDLDYWGQGTS VTVSSAKTTPPSVYPLAPGCGDTTGSSVTLGCLVKGYF PESVTVTWNSGSLSSSVHTFPALLQSGLYTMSSSVTVPS STWPSQTVTCSVAHPASSTTVDKKLEPSGPISTINPCPPC KECHKCPAPNLEGGPSVFIFPPNIKDVLMISLTPKVTCV VVDVSEDDPDVQISWFVNNVEVHTAQTQTHREDYNST IRVVSTLPIQHQDWMSGKEFKCKVNNKDLPSPIERTISK IKGLVRAPQVYILPPPAEQLSRKDVSLTCLVVGFNPGDI SVEWTSNGHTEENYKDTAPVLDSDGSYFIYSKLNMKTS KWEKTDSFSCNVRHEGLKNYYLKKTISRSPG	EIVLTQSPTTMAASPGEKITI TCSASSSISSNYLHWYQQK PGFSPKLLIYRTSNLASGVP ARFSGSGSGTSYSLTIGTME AEDVATYYCQQGGSIPLTF GAGTKLELKRADAAPTVSI FPPSSEQLTSGGASVVCFLN NFYPKDINVKWKIDGSERQ NGVLNSWTDQDSKDSTYS MSSTLTLTKDEYERHNSYT CEATHKTSTSPIVKSFNRNE C
