# Supplementary material for: Loss of cellular RNA homeostasis contributes to MDA5 activation during virus infection
Source: Nat Immunol. 2026 Aug 11;27(9):1815–28. doi: 10.1038/s41590-026-02614-3 (PMC13506345; doi:10.1038/s41590-026-02614-3)
Supplement: Supplementary file 2 [file 41590_2026_2614_MOESM2_ESM.pdf]

nature portfolio

Corresponding author(s): Natalia Sampaio

Last updated by author(s): Jul 9, 2026

Reporting Summary

Nature Portfolio wishes to improve the reproducibility of the work that we publish. This form provides structure for consistency and transparency in reporting. For further information on Nature Portfolio policies, see our Editorial Policies and the Editorial Policy Checklist.

Statistics

For all statistical analyses, confirm that the following items are present in the figure legend, table legend, main text, or Methods section.

n/a	Confirmed	
☐	☒	The exact sample size (*n*) for each experimental group/condition, given as a discrete number and unit of measurement
☐	☒	A statement on whether measurements were taken from distinct samples or whether the same sample was measured repeatedly
☐	☒	The statistical test(s) used AND whether they are one- or two-sided<br>*Only common tests should be described solely by name; describe more complex techniques in the Methods section.*
☒	☐	A description of all covariates tested
☐	☒	A description of any assumptions or corrections, such as tests of normality and adjustment for multiple comparisons
☐	☒	A full description of the statistical parameters including central tendency (e.g. means) or other basic estimates (e.g. regression coefficient) AND variation (e.g. standard deviation) or associated estimates of uncertainty (e.g. confidence intervals)
☐	☒	For null hypothesis testing, the test statistic (e.g. F, t, r) with confidence intervals, effect sizes, degrees of freedom and P value noted<br>*Give P values as exact values whenever suitable.*
☒	☐	For Bayesian analysis, information on the choice of priors and Markov chain Monte Carlo settings
☒	☐	For hierarchical and complex designs, identification of the appropriate level for tests and full reporting of outcomes
☒	☐	Estimates of effect sizes (e.g. Cohen's d, Pearson's r), indicating how they were calculated

Our web collection on statistics for biologists contains articles on many of the points above.

Software and code

Policy information about availability of computer code

Data collection	please see methods section of the manuscript
Data analysis	please see methods section of the manuscript

For manuscripts utilizing custom algorithms or software that are central to the research but not yet described in published literature, software must be made available to editors and reviewers. We strongly encourage code deposition in a community repository (e.g. GitHub). See the Nature Portfolio guidelines for submitting code & software for further information.

Data

Policy information about availability of data

All manuscripts must include a data availability statement. This statement should provide the following information, where applicable:

- Accession codes, unique identifiers, or web links for publicly available datasets
- A description of any restrictions on data availability
- For clinical datasets or third party data, please ensure that the statement adheres to our policy

All data are available in the manuscript and associated supplementary files. Any additional information required to reanalyse the data reported in this paper is available from the lead contact upon request.

Research involving human participants, their data, or biological material

Policy information about studies with human participants or human data. See also policy information about sex, gender (identity/presentation), and sexual orientation and race, ethnicity and racism.

Reporting on sex and gender	n/a
Reporting on race, ethnicity, or other socially relevant groupings	n/a
Population characteristics	n/a
Recruitment	n/a
Ethics oversight	n/a

Note that full information on the approval of the study protocol must also be provided in the manuscript.

Field-specific reporting

Please select the one below that is the best fit for your research. If you are not sure, read the appropriate sections before making your selection.

☒ Life sciences ☐ Behavioural & social sciences ☐ Ecological, evolutionary & environmental sciences

For a reference copy of the document with all sections, see nature.com/documents/nr-reporting-summary-flat.pdf

Life sciences study design

All studies must disclose on these points even when the disclosure is negative.

Sample size	Sample sizes are described in the figure legends and were based on previous experience.
Data exclusions	No data were excluded.
Replication	All attempts at replication were successful.
Randomization	Randomization was not relevant.
Blinding	Blinding was not possible as the majority of the work was carried out by a single author, and the collected data was quantifiable.

Reporting for specific materials, systems and methods

We require information from authors about some types of materials, experimental systems and methods used in many studies. Here, indicate whether each material, system or method listed is relevant to your study. If you are not sure if a list item applies to your research, read the appropriate section before selecting a response.

Materials & experimental systems

n/a	Involved in the study	
☐	☒	Antibodies
☐	☒	Eukaryotic cell lines
☒	☐	Palaeontology and archaeology
☒	☐	Animals and other organisms
☒	☐	Clinical data
☒	☐	Dual use research of concern
☒	☐	Plants

Methods

n/a	Involved in the study	
☒	☐	ChIP-seq
☐	☒	Flow cytometry
☒	☐	MRI-based neuroimaging

Antibodies

Antibodies used	please see key resources table
Validation	Antibodies were tested using either target protein knock-out cells or target protein overexpression. Otherwise, commercially

Validation	available antibodies validated by the manufacturers were used. For immunoblotting, all antibodies were diluted at 1:1000, with the exception of MDA5 mAb clone 17, which was diluted at 1:500.

Eukaryotic cell lines

Policy information about cell lines and Sex and Gender in Research

Cell line source(s)	All cell line sources are reported in the main text. All human cell lines used were of male sex.
Authentication	The cells lines were not authenticated.
Mycoplasma contamination	All cell lines were regularly tested for mycoplasma.
Commonly misidentified lines (See ICLAC register)	n/a

Flow Cytometry

Plots

Confirm that:

☒ The axis labels state the marker and fluorochrome used (e.g. CD4-FITC).

☒ The axis scales are clearly visible. Include numbers along axes only for bottom left plot of group (a 'group' is an analysis of identical markers).

☒ All plots are contour plots with outliers or pseudocolor plots.

☒ A numerical value for number of cells or percentage (with statistics) is provided.

Methodology

Sample preparation	please see methods section of the manuscript
Instrument	Attune NxT Flow Cytometer (Thermo Fisher Scientific)
Software	FlowJo software (BD)
Cell population abundance	>80%
Gating strategy	please see methods section of the manuscript

☒ Tick this box to confirm that a figure exemplifying the gating strategy is provided in the Supplementary Information.
