# Supplementary material for: Loss of cellular RNA homeostasis contributes to MDA5 activation during virus infection
Source: Nat Immunol. 2026 Aug 11;27(9):1815–28. doi: 10.1038/s41590-026-02614-3 (PMC13506345; doi:10.1038/s41590-026-02614-3)
Supplement: Supplementary file 3 [file 41590_2026_2614_MOESM3_ESM.pdf]

nature portfolio

Peer Review File

Loss of cellular RNA homeostasis contributes to MDA5 activation during virus infection

Corresponding Author: Dr Natalia Sampaio

This file contains all editorial decision letters in order by version, followed by all author rebuttals in order by version.

Version 0:

Reviewer comments:

Reviewer #1

(Remarks to the Author)
Sampaio et al have developed a tandem-affinity purification approach, based on iCLIP, to identify the RNA substrates of MDA5. This method enables the purification of endogenous MDA5-RNA complexes under stringent conditions as well as provides information of the specific region of the RNA bound to MDA5. Previously, MDA5 RNA substrates had been identified through overexpression of the MDA5 protein, low-stringency conditions or by in vitro RNAse protection assay, leading to the possibility that they may not represent true in vivo RNA targets.

By employing iCLIP, this study reveals that MDA5 predominantly binds to unprocessed endogenous cellular RNAs during infection. This unexpected and exciting discovery fills a critical gap in our understanding of the function of this sensor, and helps reconcile its role in sensing different types of pathogens, including viruses whose genome replication occurs outside MDA5 reach. Therefore, the findings in this manuscript represent a clear advance in our understanding of MDA5 function, while providing a method for purifying MDA5 substrates.

Given the challenges that the community has encountered to successfully identify MDA5 substrates, I strongly encourage authors to share all of the controls used on the samples analysed, as well as commenting on potential challenges. Additionally, providing further details to make this method generalisable to other settings or cell lines would greatly benefit the broader scientific community.

Additional valuable information on iCLIP methodology:

1.Could authors include a representative radioactive gel for the actual iCLIP samples analysed in the manuscript (WT uninfected, vs infected vs MDA5KO)? It would also be interesting to see how the radioactive signal changes in WT vs MDA5-KO cells. These images should also be used to depict the exact region that was excised for purification and sequencing. In addition, including side-by-side western blots is also beneficial to compare the levels of immunoprecipitated MDA5 in the different conditions. For instance, it is unclear if MDA5 levels change during infection.

2. Have authors compared which type of RNAs are obtained in the low vs high MW complex? (Figure 1d). Which of the complexes was finally used for the analysis?

3. The benefit of iCLIP is that this protocol uses high-stringency conditions, allowing to purify the RNA-protein complex of interest. However, the MDA5 IPs seem to contain some Actin protein (Fig 1b). Can authors comment on this and the stringency of the IP conditions? This is important to demonstrate that the RNA targets obtained are truly MDA5 substrates.

4. Why is the number of cells between iCLIP experiments so different (Calu-3 vs THP-1)?

5. Why are rRNA and tRNA excluded from the analyses? Considering that the other RLR, RIG-I can recognise rRNA during infections, it seems a bit premature to exclude them without a clear justification

6. Can the authors specify the amount of iCLIP binding sites in sense and antisense strands during EMCV and SARS-CoV-2 infection? It would provide critical information about the intra or interstranded mode of binding.

7. Why there are reads mapping to EMCV in the uninfected samples? (Figure 2A)

Additional questions about the model:

1.If MDA5 is required to activate the innate immune response against EMCV, why are the levels of EMCV RNA similar in WT vs MDA5-KO cells? (Figure S1C)

2. Authors state that ‘of the 16 regions tested, ten contained repetitive sequences and the corresponding IVT dsRNAs tended to induce stronger T1-IFN responses that the remaining IVT-dsRNAs’ (Figure S5C and S5D). Although some correlation can be drawn, there are some examples that contradict this conclusion. For instance, region ‘B’ induces IFNs as much as ‘M’ and ‘N’ but it does not contain a TE-derived sequence. Have authors tried to delete the TE-derived sequence and compare the ability to trigger IFN?

3. It is also unclear why authors decided to generate dsRNAs in vitro from the regions found to be bound by MDA5. Are both strands of those regions bound to MDA5 by iCLIP? Or, alternatively, is the antisense strand expressed. If this is not the case, how is the use of dsRNA justified?

4. MDA5 also seems to bind endogenous cellular RNA in the absence of infection. Can authors speculate why is this binding not resulting in signalling? Or is it? Have these regions also been in vitro synthesised to test if they signal?

5. The authors explore the origin of MDA5 substrates during infection, and test if these originate from intrastrand or interstrand base-pairing. I find this part confusing. If understood correctly, both intra and interstrand base-pairing explain only a very small proportion of the iCLIP sites during SARS-CoV-2 infection. If so, it still remains unclear which are the characteristics of cellular RNA that drive MDA5 recognition?

6. Are the same introns retained during EMCV and SARS-CoV-2 infection? Or are the MDA5 iCLIP binding sites virus-specific?

6. Can the authors clarify the differences between Figure 5j-k and Figure S7C? Also, what is the difference between ‘intron retention’ vs ‘unspliced pre-mRNA’ (line 324)?

7. The authors suggest that inhibition of splicing during infection is responsible for generating MDA5 substrates. There seems to be a correlation, although not extremely convincing as both introns that go up or down upon infection have MDA5 binding sites (Figure 5).

8. Authors nicely show that overexpression of SRSF3 dampens the IFN response, presumably by preventing intron accumulation and MDA5 activation. However, did the authors actually test this? (absence of intron retention upon SRSF3 overexpression?). Can authors speculate if similar results could be obtained with other splicing factors, such as hnRNPC or SRSF1, or is it specific to SRSF3?

Minor comments:
Line 93 - Can the authors clarify in the text what did they mean by ‘high molecular weight complex RNA produced during infection’? Not accessible to most readers

What is Neo 1-99 IVT? Clarify for non-expert readers

Correct figure legend S6A-B

I thoroughly enjoyed reading this paper, and I applaud the authors for the work presented. I envision these findings are not restricted to viral infections. For instance, others have also reported intron retention upon poly(I:C) stimulation or activation of the IFN response by impairing the function of important RNA processing factors or chemically inhibiting RNA splicing, supporting the relevance of the findings here reported

Reviewer #2

(Remarks to the Author)
In the manuscript “MDA5 guards against infection by surveying cellular RNA homeostasis”, the authors report that the most common immunostimulatory ligand for the PRR MDA5 is self RNA, rather than viral RNA. This conclusion is very provocative, as the authors are effectively proposing that MDA5 is not a pathogen sensor directly. Such a change in our view of MDA5 requires substantial experimental support. Below I offer suggestions on how to improve the quality of data presented.

1. The immunoprecipitations performed in Figure 1C are concerning, as the data demonstrates a high level of MDA-RNA complexes present in uninfected cells. This finding runs counter to the model proposed, as the authors suggest that MDA binding to self RNA is induced by infection and this interaction is stimulatory. How can this finding be reconciled with the model proposed?

2. Figure 3 demonstrates that depletion of viral RNA from infected cell lysates does not diminish immunostimulatory

activities. A concern with this study is the sensitivity of the innate immune response. It is possible that viral RNA remains, which is responsible for immunostimulation, despite substantial depletion of the majority of viral RNA. This issue is common in the studies of innate immunity, as very few molecules of a given PAMP (LPS or RNA) can be immunostimulatory. If the authors are correct, then they should be able to perform a similar study with a RIG-I agonistic virus, where depletion of viral RNA from infected cell lysates does diminish immunostimulatory activity. This experiment should be performed.

3. The most important functional data presented to support the model proposed is in Figure 6, where overexpression of SRSF3 (a splicing regulator) is shown to diminish interferon expression during EMCV infection. However, there is no demonstration that SRSF3 overexpression diminished the abundance of unspliced RNA in infected cells. This data is critical to provide. Additionally, the model proposed would predict that RIG-I stimulatory viruses would not be affected in the interferon stimulatory activities in SRSF3 expressing cells. This experiment should be performed.

4. Related to point 3, the analysis presented in Figure 6 is not enough to be convincing. ELISA and western analysis should be performed, as should viral replication studies, using RIG-I stimulatory and MDA stimulatory viruses. Kinetic studies are also key here.

Reviewer #3

(Remarks to the Author)
General comments

In this paper, Sampaio et al propose the interesting hypothesis that MDA5's activation is driven by binding to aberrantly spliced cellular RNA, rather than viral RNA. Based on iCLIP data, they find that, in both EMCV and SARS-CoV2 infection, there is no significant binding of MDA5 to viral RNA but that there are extensive binding sites in cellular RNA, particularly in introns. They show that some of these cellular binding sites can induce MDA5 activation. Additionally, they find that both EMCV and SARS-CoV2 infection causes disruption in splicing, which correlates MDA5's binding and that overexpression of a splicing factor can lead to a general reduction in innate immune activation. The paper is very well presented and well written. The introduction and discussion are thoughtful and comprehensive and the methods are well described.

This paper represents a novel and, given challenges in identifying MDA5's ligands, potentially very valuable contribution to the field. However, given the novelty of this finding, it is particularly important that the results are properly controlled and validated. Below, I have made some general and specific suggestions on how confidence in these findings could be strengthened.

Clearer acknowledgement and integration of prior research into MDA5's binding preferences

This paper does not fully address the fact that MDA5 is generally thought to bind to double stranded RNA. RBPs bound to dsRNA generally do not crosslink efficiently to dsRNA. Therefore, there is a possibility that iCLIP is biasing towards a subset of MDA5's RNA targets that are more permissive to crosslinking but might not be involved in MDA5 activation. The authors might want to consider an alternative means of validating that the lack of binding to vRNA is a true negative, rather than an artefact of poor crosslinking efficiency to ds target RNAs. This could be achieved using a method such as HyperTRIBE or STAMP, which relies on editing of RNA, rather than crosslinking. Alternatively, an smFISH experiment that determined whether MDA5 was colocalising with viral RNA or not could provide further evidence to support this hypothesis.

Another aspect of MDA5's binding and activation modality that could be addressed more directly is its filament formation. Given that this is thought to be an important part of its activation, determining whether binding sites result in filament formation could be a way of differentiating those that are involved in activation. Did the authors assess the distribution of crosslink sites around binding sites or identify any clusters of binding sites that might be indicative of multiple MDA5 molecules in close proximity?

Integration of iCLIP results with other available CLIP data

There is limited direct comparison between the two iCLIP datasets generated within this paper. Putting the datasets side-by-side would be a helpful way of identifying aspects of MDA5's behaviour that are consistent across different cell lines and viral models and could, therefore, strengthen many of the arguments. If the behaviour is different between the two experiments, this is also very important to highlight as it suggests that these are not universal aspects of MDA5's function.

There are also several instances where comparison to general trends in CLIP datasets or better comparison to dataset controls (mock or MDA5-KO) would be beneficial. In general, the paper would benefit from more consistent inclusion of all available iCLIP samples in each analysis as it feels somewhat selective at times. This is particularly important as the paper is based on the principle that MDA5 acquires new interaction partners upon infection, making inclusion of mock MDA5 data throughout the analyses particularly necessary for comparison. Instances of this are highlighted in the specific comments below.

Figure-specific comments

Fig1b

It would be helpful to have a silver stain here, in addition to western blot, to confirm that MDA5 is the only protein being pulled down.

Fig2a

The % of reads mapped to EMCV is the same in infected and uninfected in the RNA input (not just the IP). Is there any quality control showing that there was successful infection in this experiment? From Fig S1k, it indicates the % of infected cells but it might also be helpful to have a measure of amount of viral RNA present in the samples. Was there an attempt to select a timepoint where viral RNA levels would be maximised?

Also, I think this should be referred to as MDA5-WT, rather than WT. If I understood correctly, 'WT' here is the IRF3-KO line.

It would be helpful to have a PCA to see how the samples distribute as an additional layer of quality control.

Line 190-193

It is written in the text that there is no enrichment of EMCV RNA relative to the SMI. Perhaps this was already done and yielded no results but it would be informative to see the crosslink distribution across the viral genome. Significantly enriched reads within viral RNA are often hard to detect but visual inspection can help identify differences in patterns between IP and SMI. Given the variation in infection levels between replicates (between 10 and 30%), separating by replicate might also be beneficial in this analysis.

Fig2b

The MDA5 KO sample was not included here, although it is written in the text that the proportion is consistent between all three samples. Could MDA5-KO be included here? It would also be helpful to know how many enriched binding sites were detected in the MDA5 KO sample. This should represent noise level, as none of these can be MDA5-specific. Perhaps an analysis of overlapping binding sites between MDA5-WT and MDA5-KO binding sites would offer additional confidence that these are bona fide MDA5 binding sites. If the pattern of binding sites is the same in the MDA5-KO sample, perhaps it would be better to focus exclusively on MDA5-WT-specific binding sites and determine their distribution to ensure that this a biologically meaningful pattern and not simply a reflection of region abundance in the cell.

It would be easier to observe patterns if this figure was displayed as proportions, rather than number of binding sites.

Fig2c-e

Typically, a gene and gene-region matched control or a random scramble control would be used for motif enrichment analysis to account for overall sequence biases that do not reflect bona fide motifs. The use other samples as background is an interesting strategy to assess altered binding preferences but might be better if complemented with a more conventional analysis to ensure that these motifs are enriched overall in MDA5 binding sites. Alternatively, it would be helpful to add an indication in Fig 2e of the % of sequences containing a motif among true control sequences to have a sense of the motifs' background frequency.

Fig 3a-d

To me, this experiment feels incomplete or needs to be better explained. Firstly, the use of dsRNA is not sufficiently justified. Is there evidence that these sequences exist as dsRNA in the cell? Given that stimulation was not seen from these regions when single stranded, this would be an essential condition for being able to draw any conclusions about these RNAs being immune stimulatory in the cell.

Secondly, I couldn't tell whether these sequences are exclusively identified under infected conditions or whether they were also present in mock. This is very important information as, if sequences that are able to stimulate a response are present in both conditions, this would suggest that there was baseline activation of MDA5 under mock conditions. It might be helpful to clarify this and to add as controls additional sequences that are either present only under mock conditions or that are present

in both states.

Thirdly, the rationale behind using such large regions of RNA should be more clearly explained. Within 500nts, there could be features beyond the known MDA5 binding site that could be immunostimulatory.

Fourthly, if motifs are important for MDA5 binding and activation, were sequences containing motifs including among the sequences tested. If so, did the presence of motifs affect how immunostimulatory the RNAs were?

Fig 3e-j

Although this is acknowledged in the text, the fact that there is still residual, potentially double-stranded EMCV RNA is still concerning to me, given the strength of the claim that MDA5 activation is being exclusively driven by cellular RNA. A helpful control in this experiment would be to test whether the EMCV RNA that was depleted from the input samples was immunostimulatory when transfected on its own.

Fig 4c

This analysis might be more informative with additional frames of reference. Firstly, it might be nice to include EMCV here as well to see whether this is a consistent trend across viruses. Additionally, it would be helpful to have some controls of what the normal distribution of binding sites is among intron-binding RBPs, based on publicly available datasets. This would be important to confirm that this is an MDA5-specific binding pattern, rather than a reflection of the nature of introns and where proteins bind within them.

Fig 4f

This should say SARS CoV2, rather than EMCV I think.

Fig 4j

Why not compare MDA5 binding sites in infected and uninfected cells in this analysis, instead of comparing to random sites? It is also not clear to me how these random sites were chosen, although this might just be that I missed it.

0.4% is still a tiny fraction of binding sites. If this is MDA5's mechanism of activation, do you think that a difference between 0.1 and 0.4% is sufficient to cause MDA5 activation?

Was the same trend observed for EMCV?

Line 322

What was the rationale behind using IFN treatment as a control for the RNAseq experiment? If there are viruses that are not sensed by MDA5, could this have been an alternative control to show that this MDA5 triggering effect is not seen?

Fig 5j/k

I personally find these plots fairly unintuitive. It might be helpful to more clearly explain in the text (in addition to the figure legend) how this analysis works. It also might be helpful to have a plot that highlights more visually what proportion of introns fall within each group, perhaps as a scatter plot highlighting iCLIP hits or even a bar plot showing proportions. What proportion of MDA5's binding sites are among these altered introns?

This figure might also benefit from including mock iCLIP data. Presumably, you would expect the binding sites observed in retained introns to be specific to the infected condition. What proportion of new MDA5 binding sites following infection are among altered introns?

Fig 6

To me, the direct link with MDA5 activation is missing. If this experiment were to be performed in MDA5 KO cells, do you lose this SRSF3-overexpression-driven effect on innate immune activation? Otherwise, the phrasing of the text (lines 352-3) should be softened.

Reviewer #4

(Remarks to the Author)
A. Summary of the key results
In their manuscript 'MDA5 guards against infection by surveying cellular RNA homeostasis' Sampaio et al. describe a potentially new mechanism of MDA5 activation through alterations of cellular RNA processing. Using individual nucleotide resolution ultraviolet crosslinking and immunoprecipitation (iCLIP) of MDA5 after infection of THP-1 cells with EMCV and Calu3 cells with SARS-CoV-2, they detected overwhelmingly host RNA sequences bound to MDA5 with an enrichment for unspliced transcripts. Moreover, intron-containing pre-mRNAs were found to be increased overall in the cytoplasm of these cell lines after infection. Several of these RNA sequences found in the THP-1 cell iCLIP were then used for the basis of IVTs which induced a type-I interferon signature in MEFs and Huh7 cells. Moreover, overexpression of SRSF3 in LIM1215 cells blunted type-I IFN induction downstream of EMCV infection. Altogether, these results are indicative of a role for perturbations of RNA splicing in MDA5 activation after viral infection.

B. Originality and significance: if not novel, please include reference

The specific agonists of MDA5 remain poorly defined, this work describes a novel role of MDA5 in sensing RNA homeostasis through binding to host RNA rather than detecting viral dsRNA directly. MDA5 activation by endogenous RNA has been described in several other contexts, often involving defective or intentionally altered RNA processing or degradation in the cell(1–3) or variants of MDA5(4). However, to our knowledge, this is the first report describing self-RNA recognition as the principal driver of MDA5 activation after viral infection. Within the field, the current paradigm is that MDA5 is activated by dsRNA generated by the virus during viral replication(5, 6), although this has also been consistently questioned by reports of viruses such as paramyxoviruses, which activate MDA5 although they do not produce significant quantities of dsRNA reviewed in (7) and (8).

Indeed, if the current study manages to demonstrate that recognition of self-RNA and, in particular, mis-spliced is the central mechanism of MDA5 activation, this could potentially resolve multiple open questions, including the activation of MDA5 by spliceosome-targeted therapies, dsDNA and -ssRNA viruses and potentially sterile inflammation, which would indeed be an extremely important advance to the field.

Nonetheless, this reviewer feels that there are multiple major points that need to be addressed before such a claim is founded, and the study can be published (see below).

C. Data & methodology: validity of approach, quality of data, quality of presentation

In general, the approach and methodology are interesting and innovative, and the quality of the data and data presentation are very good.

However, there are a number of figures with relatively straightforward in vitro experiments with too few experimental replicates. To allow the authors to draw accurate conclusions, certain data should be repeated to increase n-numbers or reasons should be given as to why more experimental replicates were not possible, including:
Fig 3a-b, d: No statistics have been performed
Fig 3b: Only two experiments are shown
Fig 3d: One experiment is shown with technical duplicates --for an extremely important and rather simple control experiment (see below)!
Fig 3 e-j: only two independent experiments
Fig 6a + j: Only two independent experiments
Fig 6b: Data is representative of only one WB – shown

Beyond this, the methodology suffers somewhat both from having limited numbers of cell lines for each approach (e.g., iCLIP only in two tumor cell lines, THP-1 and Calu-3) and, at the same time, switching cell lines between approaches, e.g. iCLIP in THP-1 after EMCV infection and Calu-3 after SARS-CoV-2, then transfected IVTs in MEFs and Huh7 but reporter HEKs are used to control for MDA5 activation, then SRSF3 overexpression is conducted in LIM1215 cells.

Moreover, some of the methodological choices made in the manuscript, e.g., IRF3KOs, 500mers for the IVTs, are not clearly motivated and should be better explained or revised.

(Please see section (F) for more detailed comments.)

D. Appropriate use of statistics and treatment of uncertainties

Statistics are missing for Fig 3a-b, d and should be included. Otherwise, the use of statistics seems to be appropriate.

E. Conclusions: robustness, validity, reliability

The data presented are intriguing but require further experiments should be performed to substantiate the claims made in the manuscript.

F. Suggested improvements: experiments, data for possible revision

Major Points

1.Comparing an iChIP from IRF3KO and MDA5KO seems problematic since other factors, such as an acute antiviral response, may differentially affect the iCHIP under these conditions.
Please repeat the iCHIP from THP-1 cells using either:
- MAVSKO THP-1 vs MDA5KO/MAVSKO THP1 (which should be possible given that MDA5KO are used in the current version for iCHIP) OR
- cGASKO THP-1 vs MDA5KO/cGASKO THP1 if the more important issue is the IFN levels induced by basal cGAS activation in THP1 cells.
2. In order to substantially strengthen the overall claim of the paper, please expand the iCHIP conditions to include:
- polyI:C stimulation
- treatment with a spliceosome-targeted therapy(9), such as sudemycin D6
- infection with an -ssRNA virus or dsDNA virus that also activates MDA5 (8).
- MDA5 activation in cells that are not of malignant origin, and thus may have aberrant RNA processing per se, e.g. EMCV infection in monocyte-derived macrophages.

Are host RNA the predominant species bound to MDA5 under all of these conditions? Is polyI:C directly bound and/or does it disturb RNA homeostasis? Is there a common denominator between these diverse stimuli?

3. Replicates of experiments described in Section (C) should be completed and statistics added where appropriate for drawing conclusions. In particular, Figure 3d is based on only one experiment with weak reporter induction!

4. The IVTs induce a much, much stronger signal in MEFs and Huh7 cells than in the HEK reporter cells, which are used as a control for MDA5 activation. This difference could also be due to TLR3 activation, which is not controlled for in Figure 3d and perhaps not particular strong in the HEK cells used.

- Please repeat this experiment in TLR3KO/RIGKO Huh7 or other appropriate cell lines, including: TLR3/RIGIKO THP1, TLR3/RIGIKO HT29.
- It would further strengthen the manuscript, if the IVTs were transfected into primary cells. These could be treated with chloroquine to inhibit TLR activation and as well as a RIG-I inhibitor (RIG012), with RIG-I and TLR3 agonists as a controls. Alternatively, IVTs could be pretreated with poly-phosphatase to inhibit RIG-I activation.

5. There are multiple mechanisms through which a virus could potentially alter the spliceosome. Does EMCV target SRSF3? If not, does overexpressing SRSF3 actually restore splicing after EMCV infection? Please provide clear evidence of this.

6. The experiments for SRSF3 overexpression are only performed in LIM1215 cells, and no controls for PRR activation in general are performed.
- Please test whether SRSF3-overexpression inhibits MDA5 activation with polyI:C and RIG-I activation with 5' 3pRNA? Other nucleic-acid sensors should also be tested depending on what signaling pathways are active in these cells.
- Please overexpress SRSF3 in THP-1 and Calu3 to determine the effect on MDA5 activation in these cell lines and include appropriate controls.

7. For many viruses, the NS proteins involved in altering splicing are known, e.g. for example NS5 in dengue- or zikavirus or Nsp16 in SARS-CoV-2, reviewed in (10). Please investigate whether MDA5 activation is inhibited when a mutant virus lacking its dysregulator of RNA splicing (e.g. delNSP16 SARS-CoV-2) is used in infection experiments. This would prove that it is indeed the disturbance of splicing which is responsible for MDA5 activation by the virus investigated and greatly strengthen the manuscript.
Minor points

1. Why were 500mers chosen for the IVTs? I assume this is based on studies by other groups, but please explain.

2. According to the methods, the Illumina Tru-Seq Stranded Total RNA-Seq protocol, but it is unclear to me how this has been incorporated into the data, and when this was used to show detected of dsRNA species from the iCLIP. My apologies if I have missed this, but it would be good to explain the pipeline a bit more clearly in the manuscript.

3. Lines 212-214: I cannot follow the logic of the argumentation here. Could the authors please elaborate what the basis of

the conclusion that this is RNA from non-infected cells? What does this point add to the manuscript? Perhaps it would be better to remove this passage rather than speculate?

4. Is there a correlation between the viruses known to activate MDA5 and those known to alter RNA splicing or is this a general phenomenon of viral infection? Please discuss this point more clearly.

G. References: appropriate credit to previous work?

References and accreditations seem sufficient

H. Clarity and context: lucidity of abstract/summary, appropriateness of abstract, introduction and conclusions

There is not sufficient discussion of MDA5 within the introduction, which should include more recent findings which were first brought up the discussion. Some of these, specifically lines 377 – 384 and 398 – 401, should be moved to the introduction for a more rounded introduction to MDA5

The introduction also focuses too much on RLRs in general and could be made more concise.

The authors should acknowledge more clearly that their 'Guarding' mechanism may be only one way in which MDA5 detects viral infection within cells or provide exceptionally clear evidence that this is the "unifying theory" of viral MDA5 activation.

OTHER QUESTIONS

References for the point-to-point:

1. A. Dhir, S. Dhir, L. S. Borowski, L. Jimenez, M. Teitell, A. Rötig, Y. J. Crow, G. I. Rice, D. Duffy, C. Tamby, T. Nojima, A. Munnich, M. Schiff, C. R. de Almeida, J. Rehwinkel, A. Dziembowski, R. J. Szczesny, N. J. Proudfoot, Mitochondrial double-stranded RNA triggers antiviral signalling in humans. Nature 560, 238–242 (2018).
2. D. Roulois, H. Loo Yau, R. Singhania, Y. Wang, A. Danesh, S. Y. Shen, H. Han, G. Liang, P. A. Jones, T. J. Pugh, C. O'Brien, D. D. De Carvalho, DNA-Demethylating Agents Target Colorectal Cancer Cells by Inducing Viral Mimicry by Endogenous Transcripts. Cell 162, 961–973 (2015).
3. K. B. Chiappinelli, P. L. Strissel, A. Desrichard, H. Li, C. Henke, B. Akman, A. Hein, N. S. Rote, L. M. Cope, A. Snyder, V. Makarov, S. Budhu, S. Buhu, D. J. Slamon, J. D. Wolchok, D. M. Pardoll, M. W. Beckmann, C. A. Zahnow, T. Merghoub, T. Mergoub, T. A. Chan, S. B. Baylin, R. Strick, Inhibiting DNA Methylation Causes an Interferon Response in Cancer via dsRNA Including Endogenous Retroviruses. Cell 162, 974–986 (2015).
4. S. Ahmad, X. Mu, F. Yang, E. Greenwald, J. W. Park, E. Jacob, C.-Z. Zhang, S. Hur, Breaching Self-Tolerance to Alu Duplex RNA Underlies MDA5-Mediated Inflammation. Cell 172, 797-810.e13 (2018).
5. Q. Feng, S. V. Hato, M. A. Langereis, J. Zoll, R. Virgen-Slane, A. Peisley, S. Hur, B. L. Semler, R. P. van Rij, F. J. M. van Kuppeveld, MDA5 Detects the Double-Stranded RNA Replicative Form in Picornavirus-Infected Cells. Cell Rep. 2, 1187–1196 (2012).
6. K. Triantafilou, E. Vakakis, S. Kar, E. Richer, G. L. Evans, M. Triantafilou, Visualisation of direct interaction of MDA5 and the dsRNA replicative intermediate form of positive strand RNA viruses. J. Cell Sci. 125, 4761–4769 (2012).
7. M. Schlee, Master sensors of pathogenic RNA – RIG-I like receptors. Immunobiology 218, 1322–1335 (2013).
8. A. G. D. Junior, N. G. Sampaio, J. Rehwinkel, A Balancing Act: MDA5 in Antiviral Immunity and Autoinflammation. Trends Microbiol 27, 75–85 (2019).
9. E. A. Bowling, J. H. Wang, F. Gong, W. Wu, N. J. Neill, I. S. Kim, S. Tyagi, M. Orellana, S. J. Kurley, R. Dominguez-Vidaña, H.-C. Chung, T. Y.-T. Hsu, J. Dubrulle, A. B. Saltzman, H. Li, J. K. Meena, G. M. Canlas, S. Chamakuri, S. Singh, L. M. Simon, C. M. Olson, L. E. Dobrolecki, M. T. Lewis, B. Zhang, I. Golding, J. M. Rosen, D. W. Young, A. Malovannaya, F. Stossi, G. Miles, M. J. Ellis, L. Yu, S. Buonamici, C. Y. Lin, K. L. Karlin, X. H.-F. Zhang, T. F. Westbrook, Spliceosome-targeted therapies trigger an antiviral immune response in triple-negative breast cancer. Cell 184, 384-403.e21 (2021).
10. R. Li, S. Gao, H. Chen, X. Zhang, X. Yang, J. Zhao, Z. Wang, Virus usurps alternative splicing to clear the decks for infection. Virol. J. 20, 131 (2023).

Decision Letter:

18th Oct 2024

Dear Professor Rehwinkel,

Your Article, "MDA5 guards against infection by surveying cellular RNA homeostasis" has now been seen by 4 referees. You will see from their comments copied below that while they find your work of considerable potential interest, they have

raised quite substantial concerns that must be addressed. In light of these comments, we cannot accept the manuscript for publication, but would be very interested in considering a revised version that addresses these serious concerns.

We hope you will find the referees' comments useful as you decide how to proceed. If you wish to submit a substantially revised manuscript, please bear in mind that we will be reluctant to approach the referees again in the absence of major revisions.

We consider the revision plan that you sent us to be sensible and we would be happy to see a revised version of the manuscript that addresses the reviewers' issues following that plan.

If you choose to revise your manuscript taking into account all reviewer and editor comments, please highlight all changes in the manuscript text file.

We are committed to providing a fair and constructive peer-review process. Do not hesitate to contact us if there are specific requests from the reviewers that you believe are technically impossible or unlikely to yield a meaningful outcome.

If revising your manuscript:

* Include a “Response to referees” document detailing, point-by-point, how you addressed each referee comment. If no action was taken to address a point, you must provide a compelling argument. This response will be sent back to the referees along with the revised manuscript.

* If you have not done so already please begin to revise your manuscript so that it conforms to our Article format instructions at http://www.nature.com/ni/authors/index.html. Refer also to any guidelines provided in this letter.

* Include a revised version of any required reporting checklist. It will be available to referees (and, potentially, statisticians) to aid in their evaluation if the manuscript goes back for peer review. A revised checklist is essential for re-review of the paper.

The Reporting Summary can be found here:
https://www.nature.com/documents/nr-reporting-summary.pdf

When submitting the revised version of your manuscript, please pay close attention to our
href="https://www.nature.com/nature-portfolio/editorial-policies/image-integrity">Digital Image Integrity Guidelines. and to the following points below:

-- that unprocessed scans are clearly labelled and match the gels and western blots presented in figures.
-- that control panels for gels and western blots are appropriately described as loading on sample processing controls
-- all images in the paper are checked for duplication of panels and for splicing of gel lanes.

Extended Data figures and tables are online-only (appearing in the online PDF and full-text HTML version of the paper), peer-reviewed display items that provide essential background to the Article but are not included in the printed version of the paper due to space constraints or being of interest only to a few specialists. A maximum of ten Extended Data display items (figures and tables) is typically permitted. When re-submitting your manuscript, please ensure that any supplementary figures and tables that are more critical to the manuscript’s conclusions are converted to Extended data to increase these data’s visibility.

Finally, please ensure that you retain unprocessed data and metadata files after publication, ideally archiving data in perpetuity, as these may be requested during the peer review and production process or after publication if any issues arise.

You may use the link below to submit your revised manuscript and related files:

Link Redacted

Note: This URL links to your confidential home page and associated information about manuscripts you may have submitted, or that you are reviewing for us. If you wish to forward this email to co-authors, please delete the link to your homepage.

If you wish to submit a suitably revised manuscript we would hope to receive it within 6 months. If you cannot send it within this time, please let us know. We will be happy to consider your revision so long as nothing similar has been accepted for publication at Nature Immunology or published elsewhere.

Nature Immunology is committed to improving transparency in authorship. As part of our efforts in this direction, we are now requesting that all authors identified as ‘corresponding author’ on published papers create and link their Open Researcher and Contributor Identifier (ORCID) with their account on the Manuscript Tracking System (MTS), prior to acceptance. ORCID helps the scientific community achieve unambiguous attribution of all scholarly contributions. You can create and link your ORCID from the home page of the MTS by clicking on ‘Modify my Springer Nature account’. For more information please visit please visit www.springernature.com/orcid.

Please do not hesitate to contact me if you have any questions or would like to discuss the required revisions further.

Thank you for the opportunity to review your work.

Sincerely,
Paula

Paula Jauregui, PhD
Senior Editor
Nature Immunology

Version 1:

Reviewer comments:

Reviewer #1

(Remarks to the Author)
Thanks for addressing all my comments on the revised version of the manuscript. I particularly liked seeing the rescue of intron retention following SRSF3 overexpression.

I only have two very minor comments:
- please check the molecular wight marker in panel S2a (right panel). It may be wrong.
- please correct typo in panel S4f

Reviewer #2

(Remarks to the Author)
In this revised manuscript, the authors have attempted to address the concerns offered. The importance of the concerns offered is based on the potential impact of the discovery. The proposed model is that MDA5 should not be considered a PRR that senses viral RNA, but rather a protein that senses host RNA would have a significant impact on the field. For this reason, it was important for the authors to address the critiques with new data.

Unfortunately, the authors were not able to demonstrate changes in cytokine secretion associated with MDA5 activity. Only mRNA changes can be detected. Additionally, the authors were unable to confirm that their assay for viral RNA depletion is effective. In the absence of this proof, it is not possible for the authors to conclude that viral RNA is not the MDA5 ligand.

While this reviewer commends the authors on an interesting set of studies, it is not yet clear that the title of the manuscript: MDA5 is a guard: should be considered proven.

Reviewer #4

(Remarks to the Author)
I thank the authors for their careful and substantial revision of the manuscript. The revised version now addresses the major methodological and conceptual concerns raised in my initial review.

The iCLIP methodology is now more transparently documented, with appropriate controls including silver stain analysis, radiolabel comparisons across genotypes and infection conditions, and PCA of biological replicates. These additions strengthen confidence in the specificity and robustness of the MDA5–RNA interaction data. The authors have also clarified issues regarding viral RNA representation in size-matched input samples and infection quality control.

The expanded analysis of aberrant transcription during infection provides a more comprehensive framework linking viral infection to disruption of RNA homeostasis. Importantly, the SRSF3 rescue experiments have been substantially strengthened, including quantification of aberrant RNA species and appropriate controls using a RIG-I–activating virus (IAV) and poly(I:C) stimulation. These additions support the specificity of the proposed MDA5-dependent mechanism and reinforce the link between disrupted RNA processing and MDA5 activation.

The authors did not expand the iCLIP analysis to additional MDA5-activating contexts, such as poly(I:C) stimulation, pharmacological splicing disruption, other virus classes, or primary non-malignant cells. While inclusion of at least one such additional condition, for example, iCLIP following poly(I:C) stimulation, would likely have been feasible within the framework of the current study and would have further strengthened the generality of the proposed “guarding” mechanism, the authors now appropriately limit their conclusions to the systems directly examined and explicitly acknowledge that alternative modes of MDA5 activation may occur in other settings. In light of this more cautious framing, I do not consider the absence of these additional experiments essential for acceptance.

Overall, the revised manuscript presents a technically rigorous and conceptually significant advance. The data adequately support the conclusion that, at least in the systems examined, MDA5 predominantly binds host-derived aberrant RNAs generated during infection and that disruption of RNA homeostasis contributes to its activation. I therefore support publication in its current form.

Decision Letter:

18th May 2026

Dear Dr Sampaio,

Your Article, "MDA5 guards against infection by surveying cellular RNA homeostasis" has now been seen by 3 referees. You will see from their comments below that while they find your work of interest, some important points are raised. We are very interested in the possibility of publishing your study in Nature Immunology, but would like to consider your response to these concerns in the form of a revised manuscript before we make a final decision on publication.

Reviewer #2 has some important remaining concerns that should be addressed adding discussion and toning down conclusions. In particular, we think that you should discuss the lack of changes in cytokine secretion associated with MDA5 activity, and tone down statements and change the title to reflect your results regarding the conclusion that viral RNA is not the MDA5 ligand. Please also address the rest of the reviewers issues.

We therefore invite you to revise your manuscript taking into account all reviewer and editor comments. Please highlight all changes in the manuscript text file in Microsoft Word format.

We are committed to providing a fair and constructive peer-review process. Do not hesitate to contact us if there are specific requests from the reviewers that you believe are technically impossible or unlikely to yield a meaningful outcome.

When revising your manuscript:

* Include a “Response to referees” document detailing, point-by-point, how you addressed each referee comment. If no action was taken to address a point, you must provide a compelling argument. This response will be sent back to the referees along with the revised manuscript.

* If you have not done so already please begin to revise your manuscript so that it conforms to our Article format instructions at http://www.nature.com/ni/authors/index.html. Refer also to any guidelines provided in this letter.

* Please include a revised version of any required reporting checklist. It will be available to referees to aid in their evaluation of the manuscript goes back for peer review. They are available here:

Reporting summary:
https://www.nature.com/documents/nr-reporting-summary.pdf

When submitting the revised version of your manuscript, please pay close attention to our href="https://www.nature.com/nature-portfolio/editorial-policies/image-integrity">Digital Image Integrity Guidelines. and to the following points below:

-- that unprocessed scans are clearly labelled and match the gels and western blots presented in figures.
-- that control panels for gels and western blots are appropriately described as loading on sample processing controls
-- all images in the paper are checked for duplication of panels and for splicing of gel lanes.

Please note, Extended Data figures and tables are online-only (appearing in the online PDF and full-text HTML version of the paper), peer-reviewed display items that provide essential background to the Article but are not included in the printed version of the paper due to space constraints or being of interest only to a few specialists. A maximum of ten Extended Data display items (figures and tables) is typically permitted. When re-submitting your manuscript, please ensure that any supplementary figures and tables that are more critical to the manuscript’s conclusions are converted to Extended data to increase these data’s visibility.

Finally, please ensure that you retain unprocessed data and metadata files after publication, ideally archiving data in perpetuity, as these may be requested during the peer review and production process or after publication if any issues arise.

Please use the link below to submit your revised manuscript and related files:

Link Redacted

Note: This URL links to your confidential home page and associated information about manuscripts you may have submitted, or that you are reviewing for us. If you wish to forward this email to co-authors, please delete the link to your homepage.

We hope to receive your revised manuscript within two weeks. If you cannot send it within this time, please let us know. We will be happy to consider your revision so long as nothing similar has been accepted for publication at Nature Immunology or published elsewhere.

Please do not hesitate to contact me if you have any questions or would like to discuss these revisions further.

Nature Immunology is committed to improving transparency in authorship. As part of our efforts in this direction, we are now requesting that all authors identified as ‘corresponding author’ on published papers create and link their Open Researcher and Contributor Identifier (ORCID) with their account on the Manuscript Tracking System (MTS), prior to acceptance. ORCID helps the scientific community achieve unambiguous attribution of all scholarly contributions. You can create and link your ORCID from the home page of the MTS by clicking on ‘Modify my Springer Nature account’. For more information please visit please visit www.springernature.com/orcid.

We look forward to seeing the revised manuscript and thank you for the opportunity to review your work.

Sincerely,
Paula

Paula Jauregui, PhD
Senior Editor
Nature Immunology

Version 2:

Decision Letter:

2nd Jun 2026

Dear Dr Sampaio,

Thank you for submitting the revised version of your Article "Loss of cellular RNA homeostasis contributes to MDA5 activation during virus infection". We are very interested in the possibility of publishing your study in Nature Immunology, but we need you to change the Supplementary Figures to Extended Data Figures to facilitate our pre-acceptance checks.

When revising your manuscript:

* If you have not done so already please begin to revise your manuscript so that it conforms to our Article format instructions at http://www.nature.com/ni/authors/index.html. Refer also to any guidelines provided in this letter.

* Please include a revised version of any required reporting checklist. It will be available to referees to aid in their evaluation of the manuscript goes back for peer review. They are available here:

Reporting summary:
https://www.nature.com/documents/nr-reporting-summary.pdf

When submitting the revised version of your manuscript, please pay close attention to our href="https://www.nature.com/nature-portfolio/editorial-policies/image-integrity">Digital Image Integrity Guidelines. and to the following points below:

-- that unprocessed scans are clearly labelled and match the gels and western blots presented in figures.
-- that control panels for gels and western blots are appropriately described as loading on sample processing controls
-- all images in the paper are checked for duplication of panels and for splicing of gel lanes.

Please note, Extended Data figures and tables are online-only (appearing in the online PDF and full-text HTML version of the paper), peer-reviewed display items that provide essential background to the Article but are not included in the printed version of the paper due to space constraints or being of interest only to a few specialists. A maximum of ten Extended Data display items (figures and tables) is typically permitted. When re-submitting your manuscript, please ensure that any supplementary figures and tables that are more critical to the manuscript’s conclusions are converted to Extended data to increase these data’s visibility.

Finally, please ensure that you retain unprocessed data and metadata files after publication, ideally archiving data in perpetuity, as these may be requested during the peer review and production process or after publication if any issues arise.

Please use the link below to submit your revised manuscript and related files:

Link Redacted

Note: This URL links to your confidential home page and associated information about manuscripts you may have submitted, or that you are reviewing for us. If you wish to forward this email to co-authors, please delete the link to your homepage.

We hope to receive your revised manuscript within two weeks. If you cannot send it within this time, please let us know. We will be happy to consider your revision so long as nothing similar has been accepted for publication at Nature Immunology or published elsewhere.

Please do not hesitate to contact me if you have any questions or would like to discuss these revisions further.

Nature Immunology is committed to improving transparency in authorship. As part of our efforts in this direction, we are now requesting that all authors identified as 'corresponding author' on published papers create and link their Open Researcher and Contributor Identifier (ORCID) with their account on the Manuscript Tracking System (MTS), prior to acceptance. ORCID helps the scientific community achieve unambiguous attribution of all scholarly contributions. You can create and link your ORCID from the home page of the MTS by clicking on 'Modify my Springer Nature account'. For more information please visit please visit www.springernature.com/orcid.

We look forward to seeing the revised manuscript and thank you for the opportunity to review your work.

Sincerely,
Paula

Paula Jauregui, PhD
Senior Editor
Nature Immunology

Version 3:

Decision Letter:

Our ref: NI-A38524C

3rd Jun 2026

Dear Dr. Sampaio,

Thank you for submitting your revised manuscript "Loss of cellular RNA homeostasis contributes to MDA5 activation during virus infection" (NI-A38524C). The reviewers find that the paper has improved in revision, and therefore we'll be happy in principle to publish it in Nature Immunology, pending minor revisions to comply with our editorial and formatting guidelines.

We will now perform detailed checks on your paper and will send you a checklist detailing our editorial and formatting requirements in about a week. Please do not upload the final materials and make any revisions until you receive this additional information from us.

If you had not uploaded a Word file for the current version of the manuscript, we will need one before beginning the editing process; please email that to immunology@us.nature.com at your earliest convenience.

Thank you again for your interest in Nature Immunology Please do not hesitate to contact me if you have any questions.

Sincerely,
Paula

Paula Jauregui, PhD
Senior Editor
Nature Immunology

Open Access This Peer Review File is licensed under a Creative Commons Attribution 4.0 International License, which permits use, sharing, adaptation, distribution and reproduction in any medium or format, as long as you give appropriate credit to the original author(s) and the source, provide a link to the Creative Commons license, and indicate if changes were made.
In cases where reviewers are anonymous, credit should be given to 'Anonymous Referee' and the source.
The images or other third party material in this Peer Review File are included in the article's Creative Commons license, unless indicated otherwise in a credit line to the material. If material is not included in the article's Creative Commons license and your intended use is not permitted by statutory regulation or exceeds the permitted use, you will need to obtain permission directly from the copyright holder.
To view a copy of this license, visit https://creativecommons.org/licenses/by/4.0/

Response to reviewers for:

MDA5 guards against infection by surveying cellular RNA homeostasis

Natália G. Sampaio, Tanja Davis, Linden J. Gearing, Antonio G. Dias Junior, Lise Chauveau, Georgie Wray-McCann, Valerie Odon, Vladyslava Liudkovska, Alexandra L. McAllan, Chiara Cursi, Alice Mayer, Madara Ratnadiwakara, Minna-Liisa Änkö, Maciej Cieśla, Paul J. Hertzog, and Jan Rehwinkel

We thank all four reviewers for their encouraging comments that have helped us improve our study, and for recognising the potential impact this work may have in the field. Key revisions include:

- Expansion of experiments with *in vitro* transcribed (IVT) RNA from identified MDA5 iCLIP binding sites (Fig 3) – based on reviewers' suggestions, we now include IVT-RNAs from MDA5-binding sites from uninfected cells, and with removal of motifs or a repetitive sequence (*MIR*, a type of SINE) from select IVT-RNA. Overall, we find that most IVT-RNAs from host sequences that bind MDA5 have capacity to stimulate an MDA5-dependent interferon response.
- Removal of viral RNA depletion experiments – Based on reviewer feedback, we attempted similar experiments for IAV and EMCV RNAs, and found that depletion efficiencies were variable between experiments. Despite extensive trouble shooting efforts, we were unfortunately unable to resolve this issue; thus, we need to remove these data.
- Analysis of aberrant transcription in virus-infected cells (Fig 5) – we extended our analysis of the effects of viral infection on host RNA and found that infection generated extensive aberrant RNA expression in the cytoplasm. Additional in-depth analyses of our iCLIP data revealed that introns bound by MDA5 are predisposed to inefficient splicing, providing an possible mechanism for the generation of MDA5-stimulatory self RNAs during virus infection.
- SRSF3 overexpression rescues aberrant transcription and specifically inhibits the interferon response to EMCV (Fig 6) – based on reviewers' suggestions, we extended our study in SRSF3 cells to include IAV infection and Poly(I:C), which showed that SRSF3's effect is specific to EMCV infection. Furthermore, we confirmed that SRSF3 overexpression rescues aberrant RNA expression in cells.

Tanja Davis, Georgie Wray-McCann, Vladyslava Liudkovska, Alexandra L. McAllan and Maciej Cieśla are new co-authors of our study; they all performed new experiments or new computational analyses that are included in our revised manuscript. In addition, Natália G. Sampaio is now the sole corresponding author of the manuscript, reflecting this author's role in leading this work since initial submission.

We hope our manuscript is now sufficiently improved for publication in *Nature Immunology*.

Reviewer #1

(Remarks to the Author)
Sampaio et al have developed a tandem-affinity purification approach, based on iCLIP, to identify the RNA substrates of MDA5. This method enables the purification of endogenous MDA5-RNA complexes under stringent conditions as well as provides information of the specific region of the RNA bound to MDA5. Previously, MDA5 RNA substrates had been

identified through overexpression of the MDA5 protein, low-stringency conditions or by in vitro RNAse protection assay, leading to the possibility that they may not represent true in vivo RNA targets.

By employing iCLIP, this study reveals that MDA5 predominantly binds to unprocessed endogenous cellular RNAs during infection. This unexpected and exciting discovery fills a critical gap in our understanding of the function of this sensor, and helps reconcile its role in sensing different types of pathogens, including viruses whose genome replication occurs outside MDA5 reach. Therefore, the findings in this manuscript represent a clear advance in our understanding of MDA5 function, while providing a method for purifying MDA5 substrates.

Given the challenges that the community has encountered to successfully identify MDA5 substrates, I strongly encourage authors to share all of the controls used on the samples analysed, as well as commenting on potential challenges. Additionally, providing further details to make this method generalisable to other settings or cell lines would greatly benefit the broader scientific community.

We thank the reviewer for assessing our work and for providing helpful suggestions.

Additional valuable information on iCLIP methodology:

1.Could authors include a representative radioactive gel for the actual iCLIP samples analysed in the manuscript (WT uninfected, vs infected vs MDA5KO)? It would also be interesting to see how the radioactive signal changes in WT vs MDA5-KO cells. These images should also be used to depict the exact region that was excised for purification and sequencing. In addition, including side-by-side western blots is also beneficial to compare the levels of immunoprecipitated MDA5 in the different conditions. For instance, it is unclear if MDA5 levels change during infection.

We have included this requested data in the new Fig S2. Fig S2c shows the level of MDA5 did not change with EMCV infection, and Fig S2b and S2d show that the radiolabelling signal is strongest with the MDA5-WT EMCV-infected cells. The membrane section excised for RNA extraction is also now depicted in Fig S2d.

2. Have authors compared which type of RNAs are obtained in the low vs high MW complex? (Figure 1d). Which of the complexes was finally used for the analysis?

We have used all material running above the molecular weight of MDA5 using the low RNase A treatment condition. We apologise that this was not clear and have revised the manuscript accordingly. We refer to new data in Fig S2c to highlight the region excised for RNA extraction.

3. The benefit of iCLIP is that this protocol uses high-stringency conditions, allowing to purify the RNA-protein complex of interest. However, the MDA5 IPs seem to contain some Actin protein (Fig 1b). Can authors comment on this and the stringency of the IP conditions? This is important to demonstrate that the RNA targets obtained are truly MDA5 substrates.

We have analysed MDA5 immunoprecipitates by SDS-PAGE followed by silver stain and found that MDA5 was the most abundant protein isolated, with very few additional faint bands other than the antibody heavy and light chains. This is now shown in Fig S2a.

4. Why is the number of cells between iCLIP experiments so different (Calu-3 vs THP-1)?

We used 200-400 million THP1 cells and 50 million Calu-3 cells per condition. Calu-3 cells are significantly larger than THP1 cells and the proportion of infected cells was greater. We could therefore work with fewer.

5. Why are rRNA and tRNA excluded from the analyses? Considering that the other RLR, RIG-I can recognise rRNA during infections, it seems a bit premature to exclude them without a clear justification

Thank you for this suggestion. We have reanalysed the data whilst maintaining any sites that overlap with rRNA and tRNA, and found that the overall results remained the same, i.e. enrichment of PolyA/U motifs in infected cells, and GGUU/AACC in uninfected cells. There was detection of motifs matching rRNA, but this was in a small proportion of binding sites (~3%). Therefore, we believe that the presence or absence of rRNA and tRNA-mapped sequences does not change the findings from the mapping algorithm.

6. Can the authors specify the amount of iCLIP binding sites in sense and antisense strands during EMCV and SARS-CoV-2 infection? It would provide critical information about the intra or interstranded mode of binding.

We find that binding sites within annotated regions of the genome (i.e. in genes) are present on the sense strand for the respective gene, i.e. peaks in RNA expressed in the same direction as the encoded gene. We find no significant enrichment for binding sites in either the + or – strands in the genome. For example, for SARS-CoV-2-infected cells, there were 12,333 binding sites in the '+ strand' and 10,245 sites on the '– strand'. Of note, we did not detect binding sites in the viral genome, but only in the host.

7. Why there are reads mapping to EMCV in the uninfected samples? (Figure 2A)

This is due to mis-mapping of shorter reads. We optimised the mapping algorithm's settings to have high mapping sensitivity with minimum mis-mapping to the EMCV genome, but some mis-mapping can still occur, especially in short sequence fragments.

Additional questions about the model:

8.If MDA5 is required to activate the innate immune response against EMCV, why are the levels of EMCV RNA similar in WT vs MDA5-KO cells? (Figure S1C)

It has been well-established through seminal papers that interferon responses to EMCV infection results from MDA5 activation (e.g. Kato et al Nature 2006). However, other mechanisms also control EMCV infection *in vitro*. THP1 cells are competent in most innate immune pathways including PKR and OAS/RNase L that are known to control picornaviruses (Zhang et al, 2020, European J Immunol, https://doi.org/10.1002/eji.202048785). Thus, at the early timepoints we analysed here, replication of EMCV occurs equally in both MDA5 competent and MDA5-KO cells, though interferon responses are lost in MDA5-KO cells.

9. Authors state that 'of the 16 regions tested, ten contained repetitive sequences and the corresponding IVT dsRNAs tended to induce stronger T1-IFN responses that the remaining IVT-dsRNAs' (Figure S5C and S5D). Although some correlation can be drawn, there are some examples that contradict this conclusion. For instance, region 'B' induces IFNs as much as 'M'

and 'N' but it does not contain a TE-derived sequence. Have authors tried to delete the TE-derived sequence and compare the ability to trigger IFN?

As suggested by the reviewer, we have deleted one repetitive element as well as the discovered 'AACC' motifs from select IVT-dsRNAs. This was possible for region M, where we deleted the motif , and region N where either the motif or the *MIR* and motif were deleted (Fig S5c,d). Please note that region M entirely encompassed a *LINE1* repeat, thus it was not possible to delete this repetitive sequence. We find that deletion of the motif does not affect the stimulatory capacity of the IVT-dsRNA (Fig 3a). However, deletion of the *MIR* appeared to reduce the stimulatory capacity of the IVT-dsRNA, albeit not reaching statistical significance. This suggests that the presence of repetitive elements may enhance stimulatory capacity of endogenous RNA, though it is not entirely dependent on this.

10. It is also unclear why authors decided to generate dsRNAs in vitro from the regions found to be bound by MDA5.re both strands of those regions bound to MDA5 by iCLIP? Or, alternatively, is the antisense strand expressed. If this is not the case, how is the use of dsRNA justified?

iCLIP can only conserve a single strand bound to MDA5 due to the stringency of the purification. However, we can assume that MDA5 always targets duplex RNAs given its well-studied protein structure. Of note, we tested single stranded RNAs in Fig S6g and found them to be inert.

11. MDA5 also seems to bind endogenous cellular RNA in the absence of infection. Can authors speculate why is this binding not resulting in signalling? Or is it? Have these regions also been in vitro synthesised to test if they signal?

As suggested by the reviewer, we selected additional high-scoring MDA5-binding sites from uninfected cells, generated IVT-dsRNAs (Fig S5a), and tested their stimulatory capacity. We find that, like IVT-dsRNAs representing sites from infected cells, these were able to activate an interferon response with variable induction strength (Fig 3e).

It has been suggested in the field that MDA5 is constantly "sampling" RNA structures in the cytoplasm, but releasing these if the structure may not be stable or long enough to support oligomerisation. It is possible that MDA5 binds to host sequences in uninfected cells, but we suspect that these are not present in high enough concentration to tip MDA5 over the threshold necessary for oligomerisation and activation (Ahmad et al, 2018, Cell). Our IVT-dsRNA experiments serve to demonstrate that host RNA sequences can serve as agonists, with variability in the capacity to stimulate MDA5, for which the 'rules of engagement' still remain undefined.

12. The authors explore the origin of MDA5 substrates during infection, and test if these originate from intrastrand or interstrand base-pairing. I find this part confusing. If understood correctly, both intra and interstrand base-pairing explain only a very small proportion of the iCLIP sites during SARS-CoV-2 infection. If so, it still remains unclear which are the characteristics of cellular RNA that drive MDA5 recognition?

We appreciate the reviewer's point that the intra-strand and inter-strand base pairing only accounts for a small proportion of MDA5 binding sites. To better investigate the nature of these RNAs and what might lead to MDA5 binding, we collaborated with Maciej Ciesla (IMol Polish Academy of Sciences) to perform more in-depth analysis of the nature of the RNA sequences, specifically focussing on introns, as a major class of RNAs bound by MDA5. We found that MDA5 preferentially targets long introns with weak splice sites (Fig 5e-g), which are prone to

inappropriate splicing, and often contain repetitive elements that can form dsRNA structures. Together with the in-depth analysis of aberrant transcription shown in Fig 5a-d, we believe that this demonstrates that MDA5 is binding to host RNAs with a tendency to form base-pairing.

13. Are the same introns retained during EMCV and SARS-CoV-2 infection? Or are the MDA5 iCLIP binding sites virus-specific?

The introns bound by MDA5 are virus and/or cell-type specific, most likely due to different genes expressed in myeloid THP1 and lung Calu-3 cells. This data is now reported in Fig. 5e.

14. Can the authors clarify the differences between Figure 5j-k and Figure S7C? Also, what is the difference between 'intron retention' vs 'unspliced pre-mRNA' (line 324)?

We thank the reviewer for highlighting the need for clarity here. Intron retention refers to a mature mRNA molecule that has retained one or more introns within an otherwise canonically spliced mRNA. Unspliced pre-mRNA refers to mRNA molecules that have not been fully processed or spliced, and may represent immature or erroneous RNA (see Rogalska et al 2023, Nat Rev Genet https://doi.org:10.1038/s41576-022-00556-8.) We have clarified these distinctions and included the reference above in the text in lines 331-334:
"IRFinder identifies introns retained within otherwise canonically spliced mRNA (i.e. most of the mRNA is normally spliced, but one or more introns may have been retained), whereas index examines the overall level of intron reads on a per gene basis, which can cover both intron retention and unspliced pre-mRNA."

The enrichment plots depicted in Fig 5 have now been moved to Fig S8l-n. These are either introns measured by IRFinder (l) or index (m,n) and for EMCV-infected cells (l,m) or SARS-CoV-2-infecetd cells (n). Of note, IRFinder did not detect intron-retained RNA in the SARS-CoV-2 dataset.

15. The authors suggest that inhibition of splicing during infection is responsible for generating MDA5 substrates. There seems to be a correlation, although not extremely convincing as both introns that go up or down upon infection have MDA5 binding sites (Figure 5).

The reviewer is correct. However, we find the strongest MDA5 crosslink sites to be enriched amongst introns that accumulate in the cytoplasm (now shown in Fig S8l-n).

16. Authors nicely show that overexpression of SRSF3 dampens the IFN response, presumably by preventing intron accumulation and MDA5 activation. However, did the authors actually test this? (absence of intron retention upon SRSF3 overexpression?). Can authors speculate if similar results could be obtained with other splicing factors, such as hnRNPC or SRSF1, or is it specific to SRSF3?

We thank the reviewer for this insightful suggestion. To address this, we have performed experiments and analyses on the effect of viral infection on global aberrant RNA expression (Fig 5a-c) and find that, in addition to increased intronic RNA in the cytoplasm, there are other aberrant RNA species generated, which combined we refer to as aberrant transcription. We compared the level of aberrant transcription in LIM1215 cells +/- SRSF3 overexpression, and find that overexpression of SRSF3 on its own reduces the level of aberrant transcription in cells, even prior to viral infection (Fig 6e-f). Combined with the fact that T1-IFN response to EMCV is blunted in SRSF3 cells, despite more viral RNA being present, and that SRSF3 has

comparatively minor effects on T1-IFN response to IAV, we believe this demonstrates that aberrant transcription is the driving force behind MDA5 activation in response to EMCV.

We have not tested other splicing factors or RNA-binding proteins. Not all splicing factors can be successfully overexpressed without causing spurious effects in the cells.

Minor comments:
17. Line 93 - Can the authors clarify in the text what did they mean by 'high molecular weight complex RNA produced during infection'? Not accessible to most readers

We have elaborated the description of this RNA, aligning with the cited literature.

18. What is Neo 1-99 IVT? Clarify for non-expert readers

A description has been added to the Material and Methods section, lines 753-755.

19. Correct figure legend S6A-B

This has been corrected, thank you.

I thoroughly enjoyed reading this paper, and I applaud the authors for the work presented. I envision these findings are not restricted to viral infections. For instance, others have also reported intron retention upon poly(I:C) stimulation or activation of the IFN response by impairing the function of important RNA processing factors or chemically inhibiting RNA splicing, supporting the relevance of the findings here reported

We thank the reviewer for the positive feedback!

Reviewer #2

(Remarks to the Author)
In the manuscript "MDA5 guards against infection by surveying cellular RNA homeostasis", the authors report that the most common immunostimulatory ligand for the PRR MDA5 is self RNA, rather than viral RNA. This conclusion is very provocative, as the authors are effectively proposing that MDA5 is not a pathogen sensor directly. Such a change in our view of MDA5 requires substantial experimental support. Below I offer suggestions on how to improve the quality of data presented.

We thank the reviewer for critically evaluating our manuscript and for making constructive suggestions for improvement.

1. The immunoprecipitations performed in Figure 1C are concerning, as the data demonstrates a high level of MDA-RNA complexes present in uninfected cells. This finding runs counter to the model proposed, as the authors suggest that MDA binding to self RNA is induced by infection and this interaction is stimulatory. How can this finding be reconciled with the model proposed?

We thank the reviewer for this insightful comment. For MDA5 to survey the cytoplasm of cells for unusual RNAs, MDA5 must bind to many different types of RNAs, albeit very briefly to RNAs that are not agonists, such as in uninfected cells. These brief interactions will be crosslinked by UV light, explaining MDA5-RNA complexes purified from uninfected cells. Nonetheless, as shown in Fig 1C, and in the data provided above in response to reviewer 1 (point 1), MDA5-RNA complexes are more abundant after infection. We also note that the radiolabelled blot shown in Figure 1C may not be in the linear range. Therefore, we have included an additional result in Fig S2b and d, demonstrating that MDA5 binds RNA at a higher rate in EMCV-infected vs mock or in infected MDA5-KO cells.

2. Figure 3 demonstrates that depletion of viral RNA from infected cell lysates does not diminish immunostimulatory activities. A concern with this study is the sensitivity of the innate immune response. It is possible that viral RNA remains, which is responsible for immunostimulation, despite substantial depletion of the majority of viral RNA. This issue is common in the studies of innate immunity, as very few molecules of a given PAMP (LPS or RNA) can be immunostimulatory. If the authors are correct, then they should be able to perform a similar study with a RIG-I agonistic virus, where depletion of viral RNA from infected cell lysates does diminish immunostimulatory activity. This experiment should be performed.

We thank the reviewer for highlighting this critical point. We have attempted to perform these experiments with RNA from cells infected with either Sendai virus or IAV, two viruses that activate RIG-I. However, we have found viral RNA from these viruses could not be fully depleted. Furthermore, the sensitivity of RIG-I to its ligand is much higher than that of MDA5. It has been proposed by others that MDA5 requires a higher level of agonists present to overcome a "threshold" for activation (Ahmad et al, 2018, Cell). Thus, we believe that, if viral RNA is the true ligand, that x1000 fold depletion should reduce this to below the level required for activation. However, the reviewer is correct that we cannot entirely rule out that trace amounts of viral RNA could be present. Furthermore, we attempted to perform additional experiments for depletion of IAV and EMCV RNAs, and found that depletion efficiencies were variable between experiments. Despite extensive troubleshooting efforts, we were unfortunately unable to resolve this issue. For these reasons, we have decided to remove the viral RNA depletion data in original Fig 3e-j.

3. The most important functional data presented to support the model proposed is in Figure 6, where overexpression of SRSF3 (a splicing regulator) is shown to diminish interferon expression during EMCV infection. However, there is no demonstration that SRSF3 overexpression diminished the abundance of unspliced RNA in infected cells. This data is critical to provide. Additionally, the model proposed would predict that RIG-I stimulatory viruses would not be affected in the interferon stimulatory activities in SRSF3 expressing cells. This experiment should be performed.

Following suggestions from this and other reviewers, we performed additional experiments and undertook a more in-depth analysis of the effects of viral infections in the generation of aberrant RNA species in the cytoplasm. We re-analysed our cytoplasmic RNAseq data from infected THP1 and Calu3 cells, and performed this assay on LIM1215 cells. We found that, not only is there an increase in cytoplasmic introns in response to EMCV and SARS-CoV-2, but we see additional abnormal RNA species such as downstream, upstream, and divergent transcription. These data are now included in in the new Fig 5a-c and Fig 6e-f. From this more in-depth analysis of aberrant RNA generation during viral infection, we observed that SRSF3 overexpression overall reduced the presence of RNA resulting from aberrant transcription events.

Additionally, we have infected LIM1215-SRSF3 and GFP control cells with influenza A virus (IAV), which activates RIG-I and not MDA5. We find that SRSF3 overexpression has largely no effect on interferon/ISG responses to IAV (new Fig 6e), whereas responses to EMCV are significantly blunted across many genes (Fig 6d). Moreover, when we triggered MDA5 by transfection of high-molecular weight Poly(I:C), SRSF3 expression did not impact ISG induction (new Fig 6i), again highlighting that the role of SRSF3 is limited to controlling MDA5 activation during virus infection.

4. Related to point 3, the analysis presented in Figure 6 is not enough to be convincing. ELISA and western analysis should be performed, as should viral replication studies, using RIG-I stimulatory and MDA stimulatory viruses. Kinetic studies are also key here.

We have performed additional experiments, now shown in Fig 6 a, b, d and h-i. We measured cell viability in response to both EMCV infection and IAV infection over time using the Incucyte live cell imaging system. We find there is no significant change in cell survival in SRSF3 overexpressing vs control cells. This is the case for both EMCV and IAV. This more in-depth data has now replaced the MTT data on EMCV-infected cells previously shown in Fig 6. Notably, we measured ISG responses in cells prior to significant cell death, as indicated by the kinetic viability experiments. We attempted to perform analysis of cytokine responses at protein level by ELISA on supernatant samples but found that LIM1215 cells did not have measurable cytokine secretion in response to infection. Rather, measurement of an ISG response by qPCR was the most sensitive and reproducible technique to detect responses in these cells. We have combined the qPCR data, making it easier to visualise the changes in response to EMCV (but not IAV or Poly(I:C)) when SRSF3 is overexpressed (now Fig 6 g-i).

Reviewer #3

(Remarks to the Author)
General comments

In this paper, Sampaio et al propose the interesting hypothesis that MDA5's activation is driven by binding to aberrantly spliced cellular RNA, rather than viral RNA. Based on iCLIP data, they find that, in both EMCV and SARS-CoV2 infection, there is no significant binding of MDA5 to viral RNA but that there are extensive binding sites in cellular RNA, particularly in introns. They show that some of these cellular binding sites can induce MDA5 activation. Additionally, they find that both EMCV and SARS-CoV2 infection causes disruption in splicing, which correlates MDA5's binding and that overexpression of a splicing factor can lead to a general reduction in innate immune activation. The paper is very well presented and well written. The introduction and discussion are thoughtful and comprehensive and the methods are well described.

This paper represents a novel and, given challenges in identifying MDA5's ligands, potentially very valuable contribution to the field. However, given the novelty of this finding, it is particularly important that the results are properly controlled and validated. Below, I have made some general and specific suggestions on how confidence in these findings could be strengthened.

We thank the reviewer for their positive evaluation of our study.

1. Clearer acknowledgement and integration of prior research into MDA5's binding preferences

a) This paper does not fully address the fact that MDA5 is generally thought to bind to double stranded RNA. RBPs bound to dsRNA generally do not crosslink efficiently to dsRNA. Therefore, there is a possibility that iCLIP is biasing towards a subset of MDA5's RNA targets that are more permissive to crosslinking but might not be involved in MDA5 activation. The authors might want to consider an alternative means of validating that the lack of binding to vRNA is a true negative, rather than an artefact of poor crosslinking efficiency to ds target RNAs. This could be achieved using a method such as HyperTRIBE or STAMP, which relies on editing of RNA, rather than crosslinking. Alternatively, an smFISH experiment that determined whether MDA5 was colocalising with viral RNA or not could provide further evidence to support this hypothesis.

We thank the reviewer for these thoughtful suggestions. Unfortunately, none of these complex techniques are set up in our labs and it therefore would take a large investment of time and resources to pursue these. We believe that such studies should be the basis of separate future work. Additionally, we and others have not been successful in imaging of endogenous MDA5, making a smFISH experiment unlikely to be successful with the current antibodies available.

We attempted to perform an RNase protection assay as an alternative approach to identify MDA5 ligands during viral infection (Ahmad et al, 2018, Cell; Mehdipour et al, 2020, *Nature*). However, despite multiple attempts, we found this experimental approach to be inconsistent and provide high variability between repeats. Thus, in our hands, we do not find this to be a reliable approach to identify MDA5 ligands.

b) Another aspect of MDA5's binding and activation modality that could be addressed more directly is its filament formation. Given that this is thought to be an important part of its activation, determining whether binding sites result in filament formation could be a way of differentiating those that are involved in activation. Did the authors assess the distribution of

crosslink sites around binding sites or identify any clusters of binding sites that might be indicative of multiple MDA5 molecules in close proximity?

To identify potential clusters of crosslink sites, as suggested by the reviewer, we employed the PureCLIP algorithm with the option to identify 'binding regions', as defined by crosslink sites within 8 nucleotides of each other. We were not able to identify such clusters of binding sites. This may reflect the low level of UV crosslinking known for dsRNA-binding proteins.

We would like to highlight that MDA5 filaments have only been observed in the test tube using high concentrations of recombinant MDA5 protein and synthetic RNA. MDA5 filaments have not been reported in cells, and an alternative structural activation model has been suggested recently (doi: https://doi.org/10.1101/2024.08.27.609867).

2) Integration of iCLIP results with other available CLIP data

a) There is limited direct comparison between the two iCLIP datasets generated within this paper. Putting the datasets side-by-side would be a helpful way of identifying aspects of MDA5's behaviour that are consistent across different cell lines and viral models and could, therefore, strengthen many of the arguments. If the behaviour is different between the two experiments, this is also very important to highlight as it suggests that these are not universal aspects of MDA5's function.

b) There are also several instances where comparison to general trends in CLIP datasets or better comparison to dataset controls (mock or MDA5-KO) would be beneficial. In general, the paper would benefit from more consistent inclusion of all available iCLIP samples in each analysis as it feels somewhat selective at times. This is particularly important as the paper is based on the principle that MDA5 acquires new interaction partners upon infection, making inclusion of mock MDA5 data throughout the analyses particularly necessary for comparison. Instances of this are highlighted in the specific comments below.

We thank the reviewer for raising these important points. As shown in the new Fig 5d, there was minimal overlap between the introns bound by MDA5 in THP1 cells and Calu-3 cells, likely due to the divergent transcriptional programmes active in myeloid (THP1) and lung (Calu-3) cells. Nonetheless, the RNAs bound by MDA5 in the different iCLIP datasets shared similar features. As shown in the new Fig 5e-g, MDA5 bound to long introns that had weaker splice sites in both datasets. Moreover, the motif analyses shown in Figs 2 and 4 for the EMCV and SARS-CoV-2 datasets, respectively, revealed enrichment of the same motifs. Therefore, we believe we have analysed our datasets in an integrated manner.

Figure-specific comments

3) Fig1b

It would be helpful to have a silver stain here, in addition to western blot, to confirm that MDA5 is the only protein being pulled down.

We have performed the silver stain, and it confirms that MDA5 is the major protein pulled down in the IP (Fig S2a).

4) Fig2a

a) The % of reads mapped to EMCV is the same in infected and uninfected in the RNA input (not just the IP). Is there any quality control showing that there was successful infection in this experiment? From Fig S1k, it indicates the % of infected cells but it might also be helpful to have a measure of amount of viral RNA present in the samples. Was there an attempt to select a timepoint where viral RNA levels would be maximised?

The RNA input we sequenced for EMCV is a size-matched input (SMI) sample, which consists of cell lysate resolved on SDS-PAGE, transferred to nitrocellulose alongside IP samples, and excised from the membrane at the same molecular weight range as the IP sample. Therefore, it does not include the total RNA from a cell lysate, but only a selection that is found within this physical sample. It is quite likely that longer EMCV RNAs (~8,000 nt) would not be represented in this sample, as they would not resolve well on SDS-PAGE. Thus, we expect based on previous experiments that THP1 cells are successfully infected with EMCV at this time point and MOI (viral RNAs measured by qPCR, shown in Fig S1C, and ISG response, shown in Fig S1D), and with further verification that dsRNA is detected in the cells used for iCLIP experiments (Fig S1k) as a proxy for successful infection.

b) Also, I think this should be referred to as MDA5-WT, rather than WT. If I understood correctly, 'WT' here is the IRF3-KO line.

We agree with the reviewer and have changed the labelling of the cells in the manuscript text and figures as appropriate.

c) It would be helpful to have a PCA to see how the samples distribute as an additional layer of quality control.

We have performed the PCA and find that iCLIP replicate samples separate according to both genotype and experimental treatment (Fig. S3d and S7e).

5) Line 190-193

It is written in the text that there is no enrichment of EMCV RNA relative to the SMI. Perhaps this was already done and yielded no results but it would be informative to see the crosslink distribution across the viral genome. Significantly enriched reads within viral RNA are often hard to detect but visual inspection can help identify differences in patterns between IP and SMI. Given the variation in infection levels between replicates (between 10 and 30%), separating by replicate might also be beneficial in this analysis.

We thank the reviewer for this suggestion. We have performed visual inspection of the iCLIP data specifically across the viral genome, and this is shown in the data below. We analysed both independent replicates and combined data (shown for ease of visualisation; please note the data ranges differ between samples to allow visualisation of low abundance reads). However, we do not find instances of specific peaks in the IP samples that differ significantly from the SMI. Critically, the PureCLIP algorithm we used for this analysis uses appropriate statistical models that take into account the background and other bias factors, which is not possible by visual inspection. Thus, we believe the results from formal analysis represent the most robust interpretation of the data.

MDA5-iCLIP EMCV: combined reads

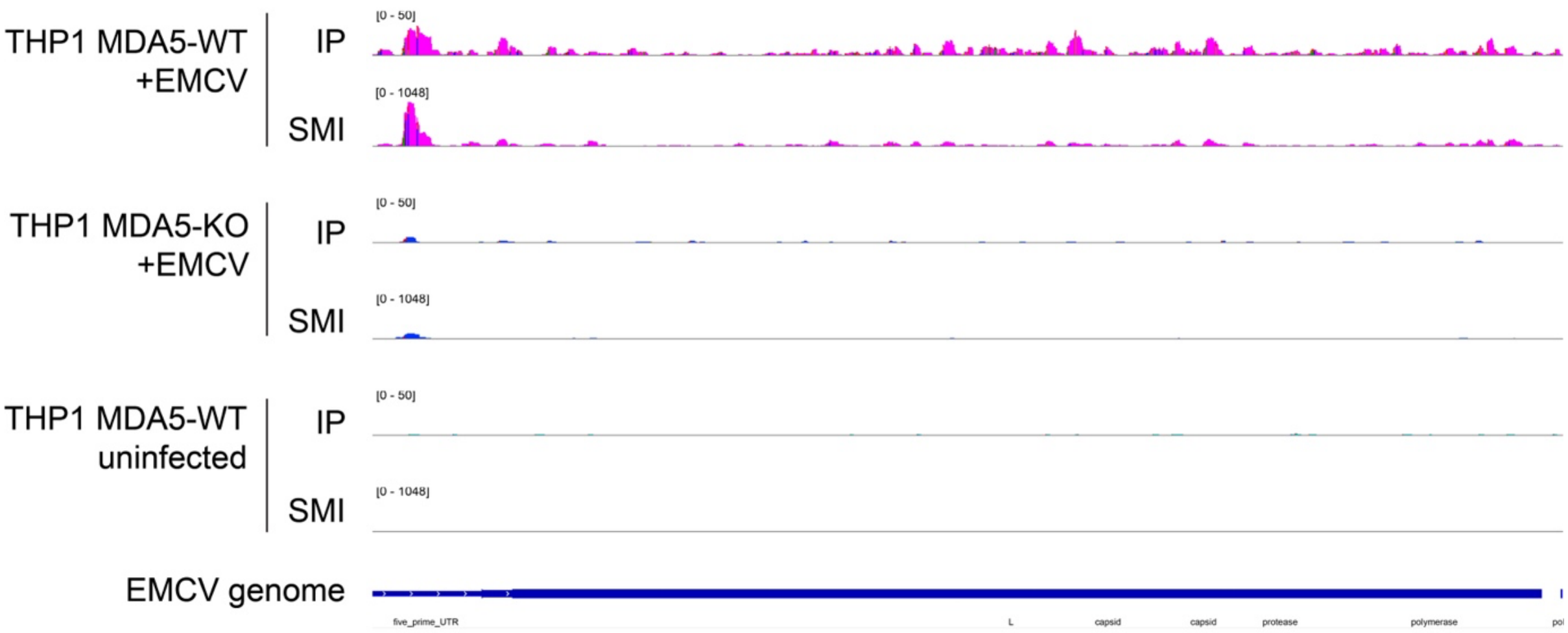

MDA5-iCLIP SARS-CoV-2: combined reads

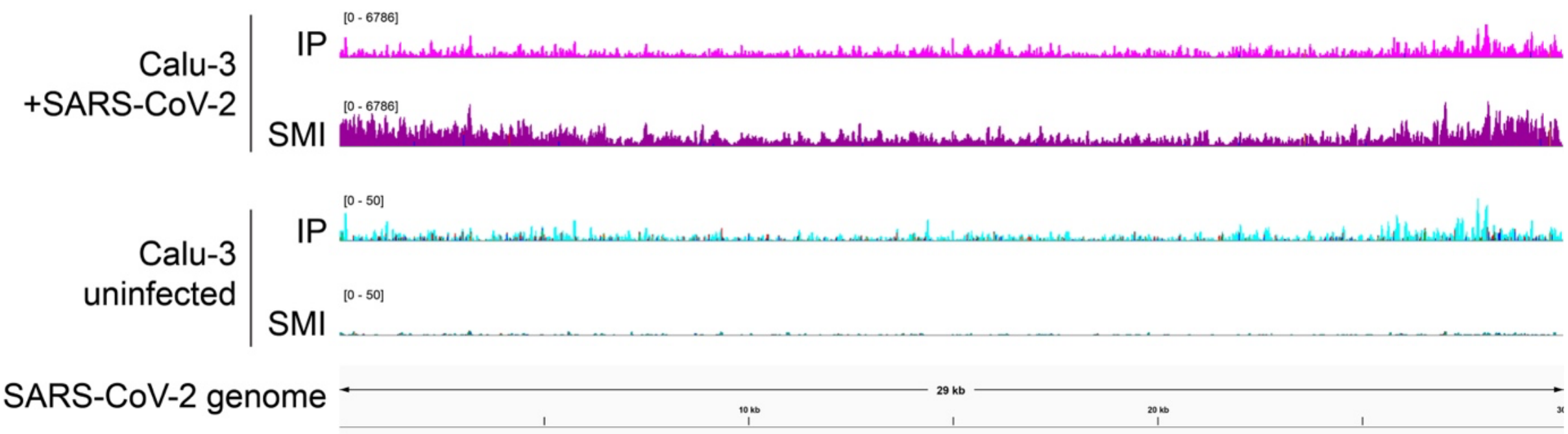

6) Fig2b

a) The MDA5 KO sample was not included here, although it is written in the text that the proportion is consistent between all three samples. Could MDA5-KO be included here? It would also be helpful to know how many enriched binding sites were detected in the MDA5 KO sample. This should represent noise level, as none of these can be MDA5-specific. Perhaps an analysis of overlapping binding sites between MDA5-WT and MDA5-KO binding sites would offer additional confidence that these are bona fide MDA5 binding sites. If the pattern of binding sites is the same in the MDA5-KO sample, perhaps it would be better to focus exclusively on MDA5-WT-specific binding sites and determine their distribution to ensure that this a biologically meaningful pattern and not simply a reflection of region abundance in the cell.

The MDA5-KO data were included in our analyses: when mapping MDA5 binding sites, we use iCLIP data from MDA5 KO control cells to rule out non-specific binding sites by removing overlapping binding sites, as the reviewer has suggested. Furthermore, the algorithm used to generate binding sites (PureCLIP) specifically takes into account the RNA abundance in the sample, by incorporating the size-matched input as a control. Thus, peaks obtained in the MDA5-WT samples represent areas enriched relative to the background expression levels in the cells.

b) It would be easier to observe patterns if this figure was displayed as proportions, rather than number of binding sites.

We can re-plot the data but believe that the number of binding sites is more relevant here. For MDA5 to survey the cytoplasm of cells for unusual RNAs, MDA5 must bind to many different types of RNAs, albeit very briefly to RNAs that are not agonists, such as in uninfected cells. These brief interactions will be crosslinked by UV light, explaining MDA5-RNA complexes purified from uninfected cells. Nonetheless, as shown in Fig 1C, and in the data provided above in response to reviewer 1 (point 1; Fig S2b,d), MDA5-RNA complexes are more abundant after infection.

7) Fig2c-e

Typically, a gene and gene-region matched control or a random scramble control would be used for motif enrichment analysis to account for overall sequence biases that do not reflect bona fide motifs. The use other samples as background is an interesting strategy to assess altered binding preferences but might be better if complemented with a more conventional analysis to ensure that these motifs are enriched overall in MDA5 binding sites. Alternatively, it would be helpful to add an indication in Fig 2e of the % of sequences containing a motif among true control sequences to have a sense of the motifs' background frequency.

The HOMER software can perform motif analysis using a randomly generated background with equal GC content to the input, and compares the test sequences against this "scramble control", as the reviewer has described. It also enables the use of a specific background file, in this case we used the size-matched input control as this file. We have performed both methods of HOMER motif analysis, and find that the discovered PolyA/PolyU and AACC/GGUU motifs remain when using both analysis methods. We note that this is already described in the methods, lines 721-724, as follows:
"The same sequences were also analysed using HOMER 56 function findMotifsGenome.pl, with -rna. Basic analysis compared test sequences to automatically generated random genetic background. Test sequences were also compared to either MDA5-KO or uninfected sequences using the -bg option."
Using both motif analysis methods, we find that the discovered PolyA/PolyU and AACC/GGUU motifs are present in less than 1% of background sequences, regardless of the background sequence employed for analysis.

For the MEME-ChIP analysis, the algorithm specifically requires experimental controls to be provided. The instructions state: "MEME-ChIP will look for motifs enriched in the primary sequences relative to this control set of sequences [...] If the primary sequences are ChIP-seq peak regions from a transcription factor ChIP-seq experiment, similar regions from a knockout cell line or organism are a possible choices for control sequences. The control sequences should be prepared in exactly the same way (e.g., repeat-masking) as the primary sequences." (taken from https://meme-suite.org/meme/doc/meme-chip.html)
Thus, for the MEME-ChIP analysis, the uninfected and KO control backgrounds we have used are most appropriate.

8) Fig 3a-d

a) To me, this experiment feels incomplete or needs to be better explained. Firstly, the use of dsRNA is not sufficiently justified. Is there evidence that these sequences exist as dsRNA in the cell? Given that stimulation was not seen from these regions when single stranded, this would be an essential condition for being able to draw any conclusions about these RNAs being immune stimulatory in the cell.

iCLIP can only conserve a single strand bound to MDA5 due to the stringency of the purification. However, we can assume that MDA5 always targets dsRNA given its well-studied protein structure. Of note, we tested single stranded RNAs in Fig S6c and found them to be inert.

b) Secondly, I couldn't tell whether these sequences are exclusively identified under infected conditions or whether they were also present in mock. This is very important information as, if sequences that are able to stimulate a response are present in both conditions, this would suggest that there was baseline activation of MDA5 under mock conditions. It might be helpful to clarify this and to add as controls additional sequences that are either present only under mock conditions or that are present in both states.

As suggested by the reviewer, we selected additional high-scoring MDA5-binding sites from uninfected cells, generated IVT-dsRNA (Fig S5b), and tested their stimulatory capacity. We find that, like IVT-dsRNA from infected cells, some of these were able to activate an interferon response with variable induction strength (Fig 3f).

Many dsRNAs at high concentrations have the potential to stimulate MDA5, as for example Poly(I:C). Our IVT-dsRNA experiments serve to demonstrate that host RNA sequences can serve as agonists, with variability in the capacity to stimulate MDA5, for which the 'rules of engagement' still remain undefined.

c) Thirdly, the rationale behind using such large regions of RNA should be more clearly explained. Within 500nts, there could be features beyond the known MDA5 binding site that could be immunostimulatory.

We chose to generate dsRNA of approximately 400-500 bp as this is the "consensus" in the field of the minimum length of dsRNA that can reliably activate MDA5 (Peisley et al, PNAS, 2012; Wu et al, Cell, 2013). The reviewer is correct in that other features may be present in the IVT-RNA that could be immunostimulatory.

d) Fourthly, if motifs are important for MDA5 binding and activation, were sequences containing motifs including among the sequences tested. If so, did the presence of motifs affect how immunostimulatory the RNAs were?

We thank the reviewer for this suggestion. To address this point, we tested the IVT RNA generated without the motifs (Fig S5c) and found that removal of the motifs did not affect their stimulatory capacity (Fig 3a). Therefore, we believe that the motifs reflect the types of RNAs being bound, e.g. polyAU stretches are a feature of the splicing signal, and are thus more common in introns. The discovery of these motifs in MDA5-binding RNA may simply be correlated with intronic regions rather than being a causation of MDA5 binding.

9) Fig 3e-j

Although this is acknowledged in the text, the fact that there is still residual, potentially double-stranded EMCV RNA is still concerning to me, given the strength of the claim that MDA5 activation is being exclusively driven by cellular RNA. A helpful control in this experiment would be to test whether the EMCV RNA that was depleted from the input samples was immunostimulatory when transfected on its own.

We thank the reviewer for this important insight. We cannot entirely rule out that trace amounts of viral RNA could be present after the viral RNA depletion. Furthermore, we attempted to

perform additional experiments for depletion of IAV and EMCV RNAs, and found that depletion efficiencies were variable between experiments. Despite extensive troubleshooting efforts, we were unfortunately unable to resolve this issue. Moreover, we found that viral RNA in the bound fractions was not recoverable in sufficient amount for transfection. For these reasons, we have decided to remove the viral RNA depletion data in original Fig 3e-j. Please also see response to reviewer #2, point 2.

10) Fig 4c

This analysis might be more informative with additional frames of reference. Firstly, it might be nice to include EMCV here as well to see whether this is a consistent trend across viruses. Additionally, it would be helpful to have some controls of what the normal distribution of binding sites is among intron-binding RBPs, based on publicly available datasets. This would be important to confirm that this is an MDA5-specific binding pattern, rather than a reflection of the nature of introns and where proteins bind within them.

As suggested by the reviewer, we have quantified the proportion of MDA5 binding sites found within repetitive elements for the iCLIP generated from EMCV-infected cells. We find that, like what we found in SARS-CoV-2-infected cells, the majority of binding sites have a repetitive element within 50 nt either side of the binding site (Fig 2c). There are some differences in composition of the repetitive elements, where for EMCV there is a larger proportion of MIR elements, which may reflect some cell type or virus-specific effects. But overall, a similar result to SARS-CoV-2 was obtained.

We appreciate the suggestion that the reviewer has made to compare the MDA5-binding data to iCLIP data from other RNA-binding proteins. However, the overwhelming majority of the iCLIP data available is from proteins that bind single-stranded RNA, which have very different modes of RNA binding and sensitivity to UV crosslinking. Instead, we revisited two published reports on iCLIP performed for dsRNA-binding proteins: DDX17 (Moy et al 2014, Cell, https://doi.org:10.1016/j.cell.2014.06.023) and Dicer (Rybak-Wolf 2015, Cell, https://doi.org:10.1016/j.cell.2014.10.040). For DDX17, authors reported that binding was enriched in coding exons, 5' UTRs, and 3' UTRs, but not introns as is seen with MDA5. For Dicer, the authors reported only a small proportion of binding sites were in repeats (~1.5%), again suggesting that the binding to repetitive elements is a particular feature of MDA5. This agrees with reported *in vitro* experiments using recombinant MDA5 incubated with cellular RNA (Ahmad et al 2018, Cell, https://doi.org:10.1016/j.cell.2017.12.016).

11) Fig 4f

This should say SARS CoV2, rather than EMCV I think.

Thank you, we have corrected the figure legend.

12) Fig 4j

Why not compare MDA5 binding sites in infected and uninfected cells in this analysis, instead of comparing to random sites? It is also not clear to me how these random sites were chosen, although this might just be that I missed it.

0.4% is still a tiny fraction of binding sites. If this is MDA5’s mechanism of activation, do you think that a difference between 0.1 and 0.4% is sufficient to cause MDA5 activation?

Was the same trend observed for EMCV?

In Fig 4i-j we aimed to determine if closely occurring binding sites could be found in opposite strands, with overlapping RNA sequences that could, in theory, form perfect complementary dsRNA stretches. As suggested by the reviewer, we have now included uninfected cells in the data. This shows that there is an enrichment of more overlapping binding sites in the infected cells compared to either uninfected or random control sites, although the fraction is very small. We were not able to perform a similar analysis for EMCV due to the iCLIP data set having fewer binding sites. Thus, there was not a reliable number of closely occurring binding sites for this analysis to be done.

We thank the reviewer for pointing out that the generation of the random control was not specified in the manuscript. This is now written in the Materials and Methods, lines 728-731: "To investigate overlapping binding sites in opposite strands, a random control dataset bed file was generated using the bedtools command random, inputting the combined human and SARS-CoV-2 genome used for iCLIP read mapping."

13) Line 322

What was the rationale behind using IFN treatment as a control for the RNAseq experiment? If there are viruses that are not sensed by MDA5, could this have been an alternative control to show that this MDA5 triggering effect is not seen?

We used IFN to show that the effect on introns was not just a by-product of the high level of gene expression induced by the ISG response. We have clarified this in the manuscript by stating "As an additional control, we treated cells with 100 U/ml IFNb to assess the effects of transcriptional changes induced by the interferon response." In lines 317-319.

14) Fig 5j/k

I personally find these plots fairly unintuitive. It might be helpful to more clearly explain in the text (in addition to the figure legend) how this analysis works. It also might be helpful to have a plot that highlights more visually what proportion of introns fall within each group, perhaps as a scatter plot highlighting iCLIP hits or even a bar plot showing proportions. What proportion of MDA5's binding sites are among these altered introns?

This figure might also benefit from including mock iCLIP data. Presumably, you would expect the binding sites observed in retained introns to be specific to the infected condition. What proportion of new MDA5 binding sites following infection are among altered introns?

We thank the reviewer for highlighting the need for more clarity here, and have elaborated our description of this data in the main text as follows: "Importantly, there was a significant positive correlation between the introns upregulated/retained in the cytoplasm of virus-infected cells with introns that contained MDA5 binding sites detected by iCLIP (Fig. S8l-n), i.e. introns that were upregulated in the cytoplasm were more likely to also overlap with introns bound by MDA5 in our iCLIP data."; lines 340-344.

We would like to highlight that we have used in this correlative analysis only the top 5% highest scoring MDA5 binding sites from our iCLIP data, thus the proportion of the top iCLIP sites that overlap with introns upregulated (by *index* analysis) were 25%, whereas 10% of these sites

overlapped with downregulated introns, and 65% did not significantly change. Furthermore, we performed the enrichment analysis on MDA5 binding sites from uninfected cells, as suggested. However, there were significantly fewer binding sites in uninfected cells, making this analysis less reliable. Please see response data below.

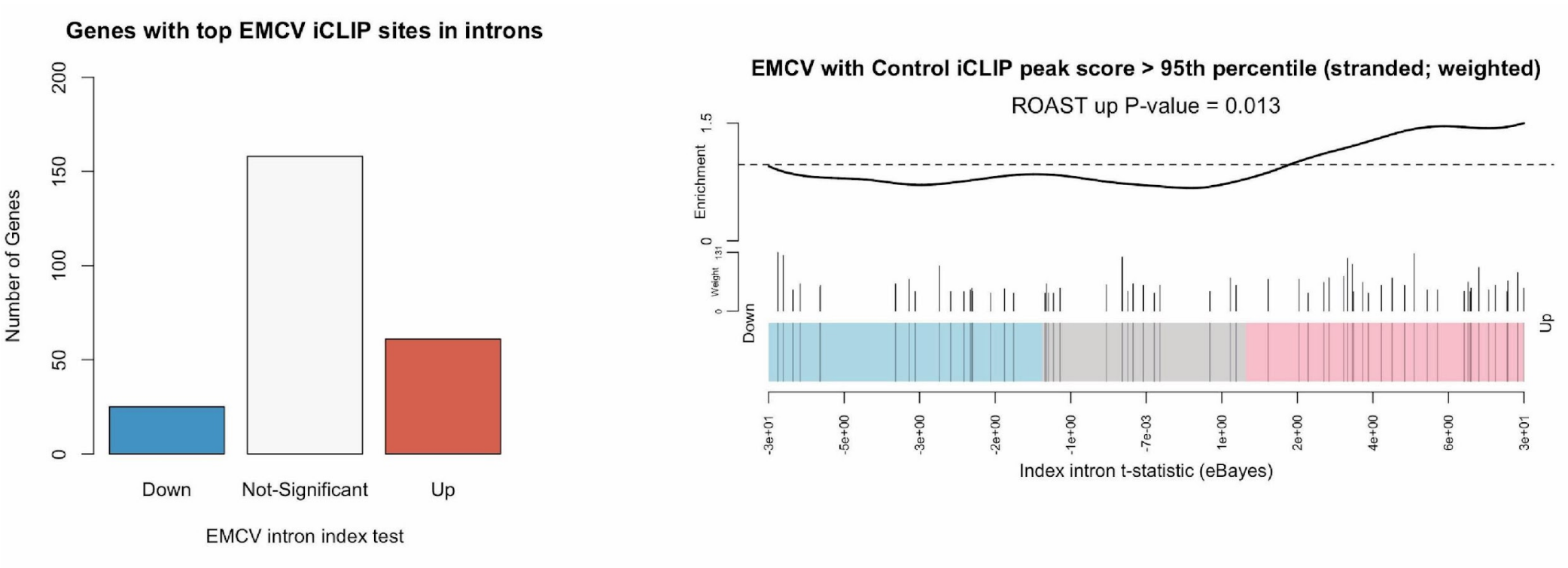

15) Fig 6

To me, the direct link with MDA5 activation is missing. If this experiment were to be performed in MDA5 KO cells, do you lose this SRSF3-overexpression-driven effect on innate immune activation? Otherwise, the phrasing of the text (lines 352-3) should be softened.

We thank the reviewer for this comment. However, given that it is very well established in many cell types and cells lines *in vitro* and in mice that EMCV activates MDA5 and not RIG-I, we believe that MDA5 KO experiments are not required.

Reviewer #4

(Remarks to the Author)
A. Summary of the key results
In their manuscript 'MDA5 guards against infection by surveying cellular RNA homeostasis' Sampaio et al. describe a potentially new mechanism of MDA5 activation through alterations of cellular RNA processing. Using individual nucleotide resolution ultraviolet crosslinking and immunoprecipitation (iCLIP) of MDA5 after infection of THP-1 cells with EMCV and Calu3 cells with SARS-CoV-2, they detected overwhelmingly host RNA sequences bound to MDA5 with an enrichment for unspliced transcripts. Moreover, intron-containing pre-mRNAs were found to be increased overall in the cytoplasm of these cell lines after infection. Several of these RNA sequences found in the THP-1 cell iCLIP were then used for the basis of IVTs which induced a type-I interferon signature in MEFs and Huh7 cells. Moreover, overexpression of SRSF3 in LIM1215 cells blunted type-I IFN induction downstream of EMCV infection. Altogether, these results are indicative of a role for perturbations of RNA splicing in MDA5 activation after viral infection.

B. Originality and significance: if not novel, please include reference

The specific agonists of MDA5 remain poorly defined, this work describes a novel role of MDA5 in sensing RNA homeostasis through binding to host RNA rather than detecting viral dsRNA directly. MDA5 activation by endogenous RNA has been described in several other contexts, often involving defective or intentionally altered RNA processing or degradation in the cell(1–3) or variants of MDA5(4). However, to our knowledge, this is the first report describing self-RNA recognition as the principal driver of MDA5 activation after viral infection. Within the field, the current paradigm is that MDA5 is activated by dsRNA generated by the virus during viral replication(5, 6), although this has also been consistently questioned by reports of viruses such as paramyxoviruses, which activate MDA5 although they do not produce significant quantities of dsRNA reviewed in (7) and (8).

Indeed, if the current study manages to demonstrate that recognition of self-RNA and, in particular, mis-spliced is the central mechanism of MDA5 activation, this could potentially resolve multiple open questions, including the activation of MDA5 by spliceosome-targeted therapies, dsDNA and -ssRNA viruses and potentially sterile inflammation, which would indeed be an extremely important advance to the field.

We thank this reviewer for highlighting the importance of our work to the field.

Nonetheless, this reviewer feels that there are multiple major points that need to be addressed before such a claim is founded, and the study can be published (see below).

C. Data & methodology: validity of approach, quality of data, quality of presentation

In general, the approach and methodology are interesting and innovative, and the quality of the data and data presentation are very good.

However, there are a number of figures with relatively straightforward in vitro experiments with too few experimental replicates. To allow the authors to draw accurate conclusions, certain data should be repeated to increase n-numbers or reasons should be given as to why more experimental replicates were not possible, including:
Fig 3a-b, d: No statistics have been performed

Fig 3b: Only two experiments are shown
Fig 3d: One experiment is shown with technical duplicates --for an extremely important and rather simple control experiment (see below)!
Fig 3 e-j: only two independent experiments
Fig 6a + j: Only two independent experiments
Fig 6b: Data is representative of only one WB – shown

As requested, we have performed additional experimental repeats, where necessary, described in the point-by-point responses below.

Beyond this, the methodology suffers somewhat both from having limited numbers of cell lines for each approach (e.g., iCLIP only in two tumor cell lines, THP-1 and Calu-3) and, at the same time, switching cell lines between approaches, e.g. iCLIP in THP-1 after EMCV infection and Calu-3 after SARS-CoV-2, then transfected IVTs in MEFs and Huh7 but reporter HEKs are used to control for MDA5 activation, then SRSF3 overexpression is conducted in LIM1215 cells.

We believe it is a strength of our study that experiments are performed in different settings, yielding similar results. We utilised THP1 cells as they have good basal MDA5 expression. We utilised Calu-3 cells as they endogenously express ACE2 and thus are susceptible to SARS-CoV-2. We used the MEFs and Huh7 cells as we have these cells with RIG-I-KO available to us. Similarly, we utilised the p125HEK reporter cell line as we had access to double MDA5-RIG-I-KO cells. The LIM1215 cells containing SRSF3 overexpression were developed by our co-author M Anko, and have been thoroughly assessed and already reported in Ratnadiwakara et al, 2018, eLife and Ratnadiwakara et al, 2023, EMBO Reports.

Moreover, some of the methodological choices made in the manuscript, e.g., IRF3KOs, 500mers for the IVTs, are not clearly motivated and should be better explained or revised.

We thank the reviewer for highlighting areas that require increased clarity. These have now been addressed as follows:
Use of IRF3-KO: Lines 137-143 “However, EMCV infectivity was low in wild-type (WT) THP1 cells (Fig. S1h). IRF3 signals downstream of multiple nucleic acid sensors including cGAS that is required for baseline T1-IFN responses in THP1 cells 53. Loss of IRF3 may therefore render cells more susceptible to EMCV without impairing MDA5-RNA interactions. Indeed, infectivity levels increased ten-fold in IRF3-KO THP1 cells 50 (Fig. S1i-k). We therefore used IRF3-KO THP1 cells to investigate MDA5 ligands generated during EMCV infection.”
For 500 nt dsRNA: Lines 230-231 “The ~500 nucleotide length was chosen based on previous reports of ideal RNA lengths for MDA5 activation 36,59.”

(Please see section (F) for more detailed comments.)

D. Appropriate use of statistics and treatment of uncertainties

Statistics are missing for Fig 3a-b, d and should be included. Otherwise, the use of statistics seems to be appropriate.

Appropriate statistical analyses have been performed, please see Fig 3.

E. Conclusions: robustness, validity, reliability

The data presented are intriguing but require further experiments should be performed to substantiate the claims made in the manuscript.

F. Suggested improvements: experiments, data for possible revision

Major Points

1.Comparing an iChIP from IRF3KO and MDA5KO seems problematic since other factors, such as an acute antiviral response, may differentially affect the iCHIP under these conditions. Please repeat the iCHIP from THP-1 cells using either:
- MAVSKO THP-1 vs MDA5KO/MAVSKO THP1 (which should be possible given that MDA5KO are used in the current version for iCHIP) OR
- cGASKO THP-1 vs MDA5KO/cGASKO THP1 if the more important issue is the IFN levels induced by basal cGAS activation in THP1 cells.

We appreciate these suggestions. However, generating clonal THP1 KO cell lines is a time-consuming endeavour, as would be repeating iCLIP that requires large numbers of cells and complex experimental and computational workflows. We therefore think this is not within the scope of revision of our current manuscript.

2. In order to substantially strengthen the overall claim of the paper, please expand the iCHIP conditions to include:
- polyI:C stimulation
- treatment with a spliceosome-targeted therapy(9), such as sudemycin D6
- infection with an -ssRNA virus or dsDNA virus that also activates MDA5 (8).
- MDA5 activation in cells that are not of malignant origin, and thus may have aberrant RNA processing per se, e.g. EMCV infection in monocyte-derived macrophages.

Are host RNA the predominant species bound to MDA5 under all of these conditions? Is polyI:C directly bound and/or does it disturb RNA homeostasis? Is there a common denominator between these diverse stimuli?

These are all great ideas by the reviewer. However, each bullet point, if done properly and thoroughly, would correspond to multiple years of work. In particular, we were limited to using cell lines, rather than primary cells (e.g. monocyte-derived macrophages as suggested) because iCLIP requires very high numbers of cells that were unfeasible using primary cell lines. Furthermore, primary cell lines from human donors have inherent high inter-donor variability, which would add a significant layer of complexity. Addressing these questions will require separate, future studies.

3. Replicates of experiments described in Section (C) should be completed and statistics added where appropriate for drawing conclusions. In particular, Figure 3d is based on only one experiment with weak reporter induction!

We have expanded the data shown in Fig 3 to include additional controls and experimental repeats. The data from Fig 3d has been significantly expanded and is now shown in Fig 3a and represents three biological repeats. Statistical analyses have been performed and are indicated in the figure.

4. The IVTs induce a much, much stronger signal in MEFs and Huh7 cells than in the HEK reporter cells, which are used as a control for MDA5 activation. This difference could also be

due to TLR3 activation, which is not controlled for in Figure 3d and perhaps not particular strong in the HEK cells used.

- Please repeat this experiment in TLR3KO/RIGKO Huh7 or other appropriate cell lines, including: TLR3/RIGIKO THP1, TLR3/RIGIKO HT29.
- It would further strengthen the manuscript, if the IVTs were transfected into primary cells. These could be treated with chloroquine to inhibit TLR activation and as well as a RIG-I inhibitor (RIG012), with RIG-I and TLR3 agonists as a controls. Alternatively, IVTs could be pretreated with poly-phosphatase to inhibit RIG-I activation.

Please note that we already use RIG-I KO cells for these experiments, thus phosphatase treatment is not required. Please also note that we transfect our IVT RNAs to the cytosol, bypassing TLR3 that is localised within endosomes and does not sample the cytoplasm. Moreover, we already demonstrate MDA5 dependency in Fig 3a. We therefore respectfully disagree that these suggested experiments are pertinent to our conclusions.

5. There are multiple mechanisms through which a virus could potentially alter the spliceosome. Does EMCV target SRSF3? If not, does overexpressing SRSF3 actually restore splicing after EMCV infection? Please provide clear evidence of this.

Based on recommendations by multiple reviewers, we have conducted a more in-depth assessment of the effects of viral infections on host RNA homeostasis and found that, in addition to increased cytoplasmic introns, infection also caused aberrant upstream, downstream, and divergent transcription of cellular RNA. We have quantified this in EMCV-infected THP1 cells, SARS-CoV-2-infected Calu-3, and EMCV-infected LIM1215 cells. We find that SRSF3 overexpression reduces the appearance of aberrant RNAs in the cytoplasm, shown in the new Fig 6e-f. In addition to promoting splicing, SRSF3 also has functions in transcription termination, mRNA transport, and microRNA processing; thus, it may rescue the aberrant transcription caused by viral infection via multiple mechanisms. Hence, SRSF3 overexpression is one approach that rescues to some extent disruption of RNA homeostasis by viral infection. Importantly, there is no effect of SRSF3 on viral infection, as viral transcripts are still high in SRSF3-overexpressing cells. Moreover, we tested the effect of SRSF3 on RIG-I activating IAV infection and found that it did not affect RIG-I-mediated responses, indicating that our phenomenon is MDA5-specific.

6. The experiments for SRSF3 overexpression are only performed in LIM1215 cells, and no controls for PRR activation in general are performed.
- Please test whether SRSF3-overexpression inhibits MDA5 activation with polyI:C and RIG-I activation with 5’ 3pRNA? Other nucleic-acid sensors should also be tested depending on what signaling pathways are active in these cells.
- Please overexpress SRSF3 in THP-1 and Calu3 to determine the effect on MDA5 activation in these cell lines and include appropriate controls.

As suggested by the reviewer, we performed stimulation of LIM1215 cells with PolyI:C and find that there is no difference in the response to PolyI:C between LIM1215-GFP and LIM1215-SRSF3-GFP cells (new Fig. 6i). Furthermore, we tested the response to IAV, a RIG-I stimulatory virus, and found the responses to be largely the same between SRSF3-overexpressing and control cells (new Fig. 6h). We attempted to overexpress SRSF3 in THP1 cells, but found this to be toxic to cells, yielding high cell death. The surviving cells did not show high SRSF3 expression levels. It may be that overexpression of SRSF3 is not well-tolerated in all cells.

7. For many viruses, the NS proteins involved in altering splicing are known, e.g. for example NS5 in dengue- or zikavirus or Nsp16 in SARS-CoV-2, reviewed in (10). Please investigate whether MDA5 activation is inhibited when a mutant virus lacking its dysregulator of RNA splicing (e.g. delNSP16 SARS-CoV-2) is used in infection experiments. This would prove that it is indeed the disturbance of splicing which is responsible for MDA5 activation by the virus investigated and greatly strengthen the manuscript.

We appreciate this suggestion by the reviewer. However, we do not have access to such mutant viruses. Moreover, the viral proteins mentioned by the reviewer are multifunctional. For example, SARS-CoV-2 Nsp16 is a component of the viral replication-transcription complex and has methyltransferase activity. Therefore, deletion of this viral protein will have many different effects on the virus and its interaction with host cells, making such experiments difficult to interpret.

Minor points

8. Why were 500mers chosen for the IVTs? I assume this is based on studies by other groups, but please explain.

We chose to generate dsRNA of approximately 400-500 bp as this is the "consensus" in the field of the length of dsRNA that can activate MDA5 (Peisley et al, PNAS, 2012; Wu et al, Cell, 2013). This is now described in the text in lines 230-231 as follows: "The ~500 nucleotide length was chosen based on previous reports of ideal RNA lengths for MDA5 activation 36,59."

9. According to the methods, the Illumina Tru-Seq Stranded Total RNA-Seq protocol, but it is unclear to me how this has been incorporated into the data, and when this was used to show detected of dsRNA species from the iCLIP. My apologies if I have missed this, but it would be good to explain the pipeline a bit more clearly in the manuscript.

We thank the reviewer for highlighting the need for more clarity. We performed Illumina Tru-Seq Stranded Total RNA-Seq on cytoplasmic RNA extracted from cells, shown in Fig. 5a-c and 6e-f. This was not performed for the iCLIP experiments, which were generated using a bespoke library preparation protocol as outlined in methods. We have clarified this in the methods by being more explicit in the section title and description, in lines 788-791:
"Cytoplasmic RNA extraction and RNA sequencing
To investigate aberrant RNA expression in cells upon virus infection, IRF3-KO THP1 cells were incubated with 10 ng/ml PMA overnight, then left untreated, infected with EMCV (MOI=2) for 20 hr, or treated with human IFNB (Rebif) for 6 hr."

10. Lines 212-214: I cannot follow the logic of the argumentation here. Could the authors please elaborate what the basis of the conclusion that this is RNA from non-infected cells? What does this point add to the manuscript? Perhaps it would be better to remove this passage rather than speculate?

We agree that more clarity was needed in this section. We believe that the GGUU/AACC motifs represent binding sites from uninfected cells because these motifs are found in higher abundance in the binding sites from uninfected cells. They are also found in binding sites from infected cells, but this sample contains a mix of infected and uninfected cells. Hence, it is likely that this motif stems from MDA5 binding sites in uninfected cells. We have revised the text to be clearer and to remove unwarranted conclusions.

11. Is there a correlation between the viruses known to activate MDA5 and those known to alter RNA splicing or is this a general phenomenon of viral infection? Please discuss this point more clearly.

We have discussed this point specifically in lines 422-431 in the manuscript, as follows: "How do viruses perturb the processing of cellular RNAs? Some viruses, including SARS-CoV-2 71, encode antagonists that directly inhibit splicing and induce intron retention, presumably to re-programme the cell towards viral gene expression 78. Dengue virus 79,80, poliovirus 66,67,69 and rhinovirus 81,82 also affect host RNA homeostasis by causing cytoplasmic relocalisation of splicing and nuclear RNA binding factors. Furthermore, MDA5 is activated by DNA virus infections, including by herpes simplex virus-1, vaccinia virus and hepatitis B virus 13,14,39. DNA viruses do not generate a replicative form viral dsRNA but interfere with splicing 78. We posit that activation of MDA5 occurs when the fidelity of RNA processing is impacted upon virus infection, either through direct viral antagonists or perhaps also indirectly through cellular stress 78."

G. References: appropriate credit to previous work?

References and accreditations seem sufficient

H. Clarity and context: lucidity of abstract/summary, appropriateness of abstract, introduction and conclusions

12. There is not sufficient discussion of MDA5 within the introduction, which should include more recent findings which were first brought up the discussion. Some of these, specifically lines 377 – 384 and 398 – 401, should be moved to the introduction for a more rounded introduction to MDA5

We thank the reviewer for this suggestion; however, we believe that the manuscript flows better with these specific sections remaining in the discussion. Thus, we have chosen to maintain these text sections as they are.

13. The introduction also focuses too much on RLRs in general and could be made more concise.

We thank the reviewer for these suggested changes. However, we believe that it is important to explain the differences in ligand identification by RIG-I compared to MDA5, as this is relevant to the shift in paradigm we are proposing in with our study. Thus, we have maintained this section in the introduction.

14. The authors should acknowledge more clearly that their 'Guarding' mechanism may be only one way in which MDA5 detects viral infection within cells or provide exceptionally clear evidence that this is the "unifying theory" of viral MDA5 activation.

We agree with the reviewer that our proposed 'guarding' mechanism may not exclude the possibility that MDA5 could be activated by viral RNAs in other infections or in other conditions. This has been acknowledged in lines 407-409: "We cannot exclude the possibility that MDA5 detects viral dsRNAs in different settings such as in cells infected with other or defective viruses, in other cell types or at different time points during infection."

OTHER QUESTIONS

References for the point-to-point:

1. A. Dhir, S. Dhir, L. S. Borowski, L. Jimenez, M. Teitell, A. Rötig, Y. J. Crow, G. I. Rice, D. Duffy, C. Tamby, T. Nojima, A. Munnich, M. Schiff, C. R. de Almeida, J. Rehwinkel, A. Dziembowski, R. J. Szczesny, N. J. Proudfoot, Mitochondrial double-stranded RNA triggers antiviral signalling in humans. Nature 560, 238–242 (2018).
2. D. Roulois, H. Loo Yau, R. Singhania, Y. Wang, A. Danesh, S. Y. Shen, H. Han, G. Liang, P. A. Jones, T. J. Pugh, C. O'Brien, D. D. De Carvalho, DNA-Demethylating Agents Target Colorectal Cancer Cells by Inducing Viral Mimicry by Endogenous Transcripts. Cell 162, 961–973 (2015).
3. K. B. Chiappinelli, P. L. Strissel, A. Desrichard, H. Li, C. Henke, B. Akman, A. Hein, N. S. Rote, L. M. Cope, A. Snyder, V. Makarov, S. Budhu, S. Buhu, D. J. Slamon, J. D. Wolchok, D. M. Pardoll, M. W. Beckmann, C. A. Zahnow, T. Merghoub, T. Mergoub, T. A. Chan, S. B. Baylin, R. Strick, Inhibiting DNA Methylation Causes an Interferon Response in Cancer via dsRNA Including Endogenous Retroviruses. Cell 162, 974–986 (2015).
4. S. Ahmad, X. Mu, F. Yang, E. Greenwald, J. W. Park, E. Jacob, C.-Z. Zhang, S. Hur, Breaching Self-Tolerance to Alu Duplex RNA Underlies MDA5-Mediated Inflammation. Cell 172, 797-810.e13 (2018).
5. Q. Feng, S. V. Hato, M. A. Langereis, J. Zoll, R. Virgen-Slane, A. Peisley, S. Hur, B. L. Semler, R. P. van Rij, F. J. M. van Kuppeveld, MDA5 Detects the Double-Stranded RNA Replicative Form in Picornavirus-Infected Cells. Cell Rep. 2, 1187–1196 (2012).
6. K. Triantafilou, E. Vakakis, S. Kar, E. Richer, G. L. Evans, M. Triantafilou, Visualisation of direct interaction of MDA5 and the dsRNA replicative intermediate form of positive strand RNA viruses. J. Cell Sci. 125, 4761–4769 (2012).
7. M. Schlee, Master sensors of pathogenic RNA – RIG-I like receptors. Immunobiology 218, 1322–1335 (2013).
8. A. G. D. Junior, N. G. Sampaio, J. Rehwinkel, A Balancing Act: MDA5 in Antiviral Immunity and Autoinflammation. Trends Microbiol 27, 75–85 (2019).
9. E. A. Bowling, J. H. Wang, F. Gong, W. Wu, N. J. Neill, I. S. Kim, S. Tyagi, M. Orellana, S. J. Kurley, R. Dominguez-Vidaña, H.-C. Chung, T. Y.-T. Hsu, J. Dubrulle, A. B. Saltzman, H. Li, J. K. Meena, G. M. Canlas, S. Chamakuri, S. Singh, L. M. Simon, C. M. Olson, L. E. Dobrolecki, M. T. Lewis, B. Zhang, I. Golding, J. M. Rosen, D. W. Young, A. Malovannaya, F. Stossi, G. Miles, M. J. Ellis, L. Yu, S. Buonamici, C. Y. Lin, K. L. Karlin, X. H.-F. Zhang, T. F. Westbrook, Spliceosome-targeted therapies trigger an antiviral immune response in triple-negative breast cancer. Cell 184, 384-403.e21 (2021).
10. R. Li, S. Gao, H. Chen, X. Zhang, X. Yang, J. Zhao, Z. Wang, Virus usurps alternative splicing to clear the decks for infection. Virol. J. 20, 131 (2023).

Response to editor's comments:

Your Article, "MDA5 guards against infection by surveying cellular RNA homeostasis" has now been seen by 3 referees. You will see from their comments below that while they find your work of interest, some important points are raised. We are very interested in the possibility of publishing your study in Nature Immunology, but would like to consider your response to these concerns in the form of a revised manuscript before we make a final decision on publication.

Reviewer #2 has some important remaining concerns that should be addressed adding discussion and toning down conclusions. In particular, we think that you should discuss the lack of changes in cytokine secretion associated with MDA5 activity, and tone down statements and change the title to reflect your results regarding the conclusion that viral RNA is not the MDA5 ligand. Please also address the rest of the reviewers issues.

We thank the editor for their continued consideration of our manuscript for publication in *Nature Immunology*. As requested, we have toned down the conclusions within our Abstract and Discussion sections, as well as in the last paragraph of the introduction, described below in the response to Reviewer #2. We have added a statement in the Results section highlighting the lack of detectable cytokine responses from LIM1215 cells. We have also changed the title of the manuscript to more closely reflect the conclusions drawn from our work.

In addition to the required changes, we have made one additional change to Fig 1a. We have come to realise that the data shown in Fig 1a was generated with an earlier version of the anti-MDA5 antibodies (a mix of mouse ascites fluids from selected hybridoma cells, including mAb 22) rather than the recombinant mAb 22 antibody used for the iCLIP experiments. We therefore replaced this panel with comparable data that was generated using the recombinant mAb 22. This is now shown in Fig 1a and validates the dual IP approach we used for subsequent iCLIP experiments. Moreover, some of the radiolabelling experiments (Fig 1c-e) were also performed using the same mouse ascites fluid mix for IP2. We have now stated this in the materials and methods section and the figure legend. We would like to clarify that although these initial radiolabelling experiments were not done using the recombinant antibody, equivalent radiolabelling data can be seen for the iCLIP experiments (Fig S2d) demonstrating that the recombinant mAb22 successfully isolates MDA5 with bound RNA specifically during EMCV infection.

Lastly, we have added to the manuscript the amino acid sequences of our monoclonal antibodies as supplementary information (Table S3). We believe providing this as an open resource to the scientific community will support continued advances in MDA5 research.

Response to reviewers' comments:

Reviewer #1 (Remarks to the Author):

Thanks for addressing all my comments on the revised version of the manuscript. I particularly liked seeing the rescue of intron retention following SRSF3 overexpression.

I only have two very minor comments:
- please check the molecular wight marker in panel S2a (right panel). It may be wrong.
- please correct typo in panel S4f

We thank the reviewer for their insightful comments, which have contributed to improving our final manuscript. We have made the requested corrections in the figures.

Reviewer #2 (Remarks to the Author):

In this revised manuscript, the authors have attempted to address the concerns offered. The importance of the concerns offered is based on the potential impact of the discovery. The proposed model is that MDA5 should not be considered a PRR that senses viral RNA, but rather a protein that senses host RNA would have a significant impact on the field. For this reason, it was important for the authors to address the critiques with new data.

Unfortunately, the authors were not able to demonstrate changes in cytokine secretion associated with MDA5 activity. Only mRNA changes can be detected. Additionally, the authors were unable to confirm that their assay for viral RNA depletion is effective. In the absence of this proof, it is not possible for the authors to conclude that viral RNA is not the MDA5 ligand.

While this reviewer commends the authors on an interesting set of studies, it is not yet clear that the title of the manuscript: MDA5 is a guard: should be considered proven.

We thank the reviewer for highlighting the potential impact our study could have in the field of MDA5 sensing. We appreciate that our data cannot irrevocably conclude that MDA5 is NOT a sensor of viral RNA. Whilst this was already clearly stated in our discussion, we have now changed the manuscript title to reflect this better, and have made the following alterations in the text:

- Removed from the abstract the following text: "establishing a paradigm of innate immune 'guarding' for RNA sensors.", and edited the following sentence "Taken ***together, we propose that*** MDA5 surveys RNA processing fidelity and ***can*** detect infections by sensing perturbations of posttranscriptional events such as splicing." (lines 35-37)
- Reworded the final paragraphs in the Introduction: "This suggests that MDA5 ***can*** detect imbalances in RNA processing occurring in virus-infected cells by binding to endogenous host intronic RNA accumulating in the cytoplasm." (lines 121.124)
- Added the following statement in section Results section 'Reduction of aberrant transcripts during infection abrogates MDA5 activation': "Of note, we measured IP-10 in cell culture supernatant as a readout of cytokine responses but found that LIM1215 cells did not release significant IP-10 in response to viral infections." (lines 388-390)
- Toning down conclusions within the Discussion, for example "Our work thus ***proposes*** innate immune guarding as a ***potential mechanism*** underpinning cytoplasmic RNA sensing during virus infection" (lines 414-415); "Nonetheless, ***our interpretation*** – in which MDA5 guards posttranscriptional control – provides an attractive explanation as to why MDA5 can detect many different infections that are unlikely to all generate the same PAMP" (lines 421-424); and "Taken together, our work shifts the understanding of MDA5 activation ***during infection***: rather than detecting ***exclusively*** viral RNA as a PAMP, as is the case with other PRRs, we propose that MDA5 ***can also function as*** a sensor of cellular RNA homeostasis." (lines 501-503)

We note that we only measured secreted IP-10 from LIM1215 cells in response to viral infections, as in our experience we find that this cytokine can often be detected in supernatants of other cells when T1-IFNs are undetectable. However, we found that LIM1215 cells induce only marginal IP-10 levels upon virus infection. We concede that measuring only IP-10 is limited, and other cytokines could have possibly been detected. However, we no longer have access to additional samples for testing. We believe that repeating these experiments solely to obtain supernatants for additional cytokine testing, which may not yield results, is an unnecessary delay to publishing our work. We do

not believe that such data would substantially add to the paper, or change the conclusions drawn.

Reviewer #4 (Remarks to the Author):

I thank the authors for their careful and substantial revision of the manuscript. The revised version now addresses the major methodological and conceptual concerns raised in my initial review.

The iCLIP methodology is now more transparently documented, with appropriate controls including silver stain analysis, radiolabel comparisons across genotypes and infection conditions, and PCA of biological replicates. These additions strengthen confidence in the specificity and robustness of the MDA5–RNA interaction data. The authors have also clarified issues regarding viral RNA representation in size-matched input samples and infection quality control.

The expanded analysis of aberrant transcription during infection provides a more comprehensive framework linking viral infection to disruption of RNA homeostasis. Importantly, the SRSF3 rescue experiments have been substantially strengthened, including quantification of aberrant RNA species and appropriate controls using a RIG-I–activating virus (IAV) and poly(I:C) stimulation. These additions support the specificity of the proposed MDA5-dependent mechanism and reinforce the link between disrupted RNA processing and MDA5 activation.

The authors did not expand the iCLIP analysis to additional MDA5-activating contexts, such as poly(I:C) stimulation, pharmacological splicing disruption, other virus classes, or primary non-malignant cells. While inclusion of at least one such additional condition, for example, iCLIP following poly(I:C) stimulation, would likely have been feasible within the framework of the current study and would have further strengthened the generality of the proposed “guarding” mechanism, the authors now appropriately limit their conclusions to the systems directly examined and explicitly acknowledge that alternative modes of MDA5 activation may occur in other settings. In light of this more cautious framing, I do not consider the absence of these additional experiments essential for acceptance.

Overall, the revised manuscript presents a technically rigorous and conceptually significant advance. The data adequately support the conclusion that, at least in the systems examined, MDA5 predominantly binds host-derived aberrant RNAs generated during infection and that disruption of RNA homeostasis contributes to its activation. I therefore support publication in its current form.

We sincerely thank the reviewer for their contribution to improving our manuscript by providing critical comments. Based on additional comments from Reviewer #2, we have toned down our conclusions and changed the title of the manuscript to align with the findings of this study more closely (please see these changes described in response to Reviewer #2 above).

Response to editor's comments:

Your Article, "MDA5 guards against infection by surveying cellular RNA homeostasis" has now been seen by 3 referees. You will see from their comments below that while they find your work of interest, some important points are raised. We are very interested in the possibility of publishing your study in Nature Immunology, but would like to consider your response to these concerns in the form of a revised manuscript before we make a final decision on publication.

Reviewer #2 has some important remaining concerns that should be addressed adding discussion and toning down conclusions. In particular, we think that you should discuss the lack of changes in cytokine secretion associated with MDA5 activity, and tone down statements and change the title to reflect your results regarding the conclusion that viral RNA is not the MDA5 ligand. Please also address the rest of the reviewers issues.

We thank the editor for their continued consideration of our manuscript for publication in *Nature Immunology*. As requested, we have toned down the conclusions within our Abstract and Discussion sections, as well as in the last paragraph of the introduction, described below in the response to Reviewer #2. We have added a statement in the Results section highlighting the lack of detectable cytokine responses from LIM1215 cells. We have also changed the title of the manuscript to more closely reflect the conclusions drawn from our work.

In addition to the required changes, we have made one additional change to Fig 1a. We have come to realise that the data shown in Fig 1a was generated with an earlier version of the anti-MDA5 antibodies (a mix of mouse ascites fluids from selected hybridoma cells, including mAb 22) rather than the recombinant mAb 22 antibody used for the iCLIP experiments. We therefore replaced this panel with comparable data that was generated using the recombinant mAb 22. This is now shown in Fig 1a and validates the dual IP approach we used for subsequent iCLIP experiments. Moreover, some of the radiolabelling experiments (Fig 1c-e) were also performed using the same mouse ascites fluid mix for IP2. We have now stated this in the materials and methods section and the figure legend. We would like to clarify that although these initial radiolabelling experiments were not done using the recombinant antibody, equivalent radiolabelling data can be seen for the iCLIP experiments (Fig S2d) demonstrating that the recombinant mAb22 successfully isolates MDA5 with bound RNA specifically during EMCV infection.

Lastly, we have added to the manuscript the amino acid sequences of our monoclonal antibodies as supplementary information (Table S3). We believe providing this as an open resource to the scientific community will support continued advances in MDA5 research.

Response to reviewers' comments:

Reviewer #1 (Remarks to the Author):

Thanks for addressing all my comments on the revised version of the manuscript. I particularly liked seeing the rescue of intron retention following SRSF3 overexpression.

I only have two very minor comments:
- please check the molecular wight marker in panel S2a (right panel). It may be wrong.
- please correct typo in panel S4f

We thank the reviewer for their insightful comments, which have contributed to improving our final manuscript. We have made the requested corrections in the figures.

Reviewer #2 (Remarks to the Author):

In this revised manuscript, the authors have attempted to address the concerns offered. The importance of the concerns offered is based on the potential impact of the discovery. The proposed model is that MDA5 should not be considered a PRR that senses viral RNA, but rather a protein that senses host RNA would have a significant impact on the field. For this reason, it was important for the authors to address the critiques with new data.

Unfortunately, the authors were not able to demonstrate changes in cytokine secretion associated with MDA5 activity. Only mRNA changes can be detected. Additionally, the authors were unable to confirm that their assay for viral RNA depletion is effective. In the absence of this proof, it is not possible for the authors to conclude that viral RNA is not the MDA5 ligand.

While this reviewer commends the authors on an interesting set of studies, it is not yet clear that the title of the manuscript: MDA5 is a guard: should be considered proven.

We thank the reviewer for highlighting the potential impact our study could have in the field of MDA5 sensing. We appreciate that our data cannot irrevocably conclude that MDA5 is NOT a sensor of viral RNA. Whilst this was already clearly stated in our discussion, we have now changed the manuscript title to reflect this better, and have made the following alterations in the text:

- Removed from the abstract the following text: "establishing a paradigm of innate immune 'guarding' for RNA sensors.", and edited the following sentence "Taken ***together, we propose that*** MDA5 surveys RNA processing fidelity and ***can*** detect infections by sensing perturbations of posttranscriptional events such as splicing." (lines 35-37)
- Reworded the final paragraphs in the Introduction: "This suggests that MDA5 ***can*** detect imbalances in RNA processing occurring in virus-infected cells by binding to endogenous host intronic RNA accumulating in the cytoplasm." (lines 121.124)
- Added the following statement in Results section 'Reduction of aberrant transcripts during infection abrogates MDA5 activation': "Of note, we measured IP-10 in cell culture supernatant as a readout of cytokine responses but found that LIM1215 cells did not release significant IP-10 in response to viral infections." (lines 388-390)
- Toning down conclusions within the Discussion, for example "Our work thus ***proposes*** innate immune guarding as a ***potential mechanism*** underpinning cytoplasmic RNA sensing during virus infection" (lines 414-415); "Nonetheless, ***our interpretation*** – in which MDA5 guards posttranscriptional control – provides an attractive explanation as to why MDA5 can detect many different infections that are unlikely to all generate the same PAMP" (lines 421-424); and "Taken together, our work shifts the understanding of MDA5 activation ***during infection***: rather than detecting ***exclusively*** viral RNA as a PAMP, as is the case with other PRRs, we propose that MDA5 ***can also function as*** a sensor of cellular RNA homeostasis." (lines 501-503)

We note that we only measured secreted IP-10 from LIM1215 cells in response to viral infections, as in our experience we find that this cytokine can often be detected in supernatants of other cells when T1-IFNs are undetectable. However, we found that LIM1215 cells induce only marginal IP-10 levels upon virus infection. We concede that measuring only IP-10 is limited, and other cytokines could have possibly been detected. However, we no longer have access to additional samples for testing. We believe that repeating these experiments solely to obtain supernatants for additional cytokine testing, which may not yield results, is an unnecessary delay to publishing our work. We do

not believe that such data would substantially add to the paper, or change the conclusions drawn.

Reviewer #4 (Remarks to the Author):

I thank the authors for their careful and substantial revision of the manuscript. The revised version now addresses the major methodological and conceptual concerns raised in my initial review.

The iCLIP methodology is now more transparently documented, with appropriate controls including silver stain analysis, radiolabel comparisons across genotypes and infection conditions, and PCA of biological replicates. These additions strengthen confidence in the specificity and robustness of the MDA5–RNA interaction data. The authors have also clarified issues regarding viral RNA representation in size-matched input samples and infection quality control.

The expanded analysis of aberrant transcription during infection provides a more comprehensive framework linking viral infection to disruption of RNA homeostasis. Importantly, the SRSF3 rescue experiments have been substantially strengthened, including quantification of aberrant RNA species and appropriate controls using a RIG-I–activating virus (IAV) and poly(I:C) stimulation. These additions support the specificity of the proposed MDA5-dependent mechanism and reinforce the link between disrupted RNA processing and MDA5 activation.

The authors did not expand the iCLIP analysis to additional MDA5-activating contexts, such as poly(I:C) stimulation, pharmacological splicing disruption, other virus classes, or primary non-malignant cells. While inclusion of at least one such additional condition, for example, iCLIP following poly(I:C) stimulation, would likely have been feasible within the framework of the current study and would have further strengthened the generality of the proposed “guarding” mechanism, the authors now appropriately limit their conclusions to the systems directly examined and explicitly acknowledge that alternative modes of MDA5 activation may occur in other settings. In light of this more cautious framing, I do not consider the absence of these additional experiments essential for acceptance.

Overall, the revised manuscript presents a technically rigorous and conceptually significant advance. The data adequately support the conclusion that, at least in the systems examined, MDA5 predominantly binds host-derived aberrant RNAs generated during infection and that disruption of RNA homeostasis contributes to its activation. I therefore support publication in its current form.

We sincerely thank the reviewer for their contribution to improving our manuscript by providing critical comments. Based on additional comments from Reviewer #2, we have toned down our conclusions and changed the title of the manuscript to align with the findings of this study more closely (please see these changes described in response to Reviewer #2 above).
